# Hepatic SEC16B regulates lipid homeostasis by coordinating VLDL secretion and lipid droplet expansion

**DOI:** 10.1172/JCI204602

**Published:** 2026-04-24

**Authors:** Wei Lu, Zhiming Zhao, Donald Molina, Huaxun Fan, Ruicheng Shi, Ye Tian, Raja Gopoju, Tiantian Yang, Xinyuan Zhang, Yanqiao Zhang, Kai Zhang, Jaume Amengual, Bo Wang

**Affiliations:** 1 Department of Comparative Biosciences, College of Veterinary Medicine,; 2 Department of Food Science and Human Nutrition, and; 3 Department of Biochemistry, School of Molecular & Cellular Biology, College of Liberal Arts & Sciences, University of Illinois Urbana-Champaign, Urbana, Illinois, USA.; 4 Department of Internal Medicine, University of Arizona College of Medicine, Phoenix, Arizona, USA.; 5 Cancer Center at Illinois,; 6 Division of Nutritional Sciences, College of Agricultural, Consumer and Environmental Sciences and; 7 Department of Molecular & Integrative Physiology, School of Molecular & Cellular Biology, College of Liberal Arts & Sciences, University of Illinois Urbana-Champaign, Urbana, Illinois, USA.

**Keywords:** Hepatology, Metabolism, Atherosclerosis, Cardiovascular disease, Lipoproteins

## Abstract

The liver plays a critical role in lipid homeostasis, where lipids are either secreted as VLDLs or stored in lipid droplets (LDs). However, the regulatory mechanisms governing these 2 interconnected processes remain poorly understood. Here, we demonstrate that SEC16B functions as a lipid-responsive regulator in the liver, promoting VLDL secretion and LD expansion to handle lipid flux and maintain lipid homeostasis. Genome-wide association studies have identified SNPs in *SEC16B* to be highly associated with serum lipid levels in humans. Hepatic *Sec16b* deficiency decreases serum lipid levels by impairing VLDL secretion via disruption of COPII-mediated intracellular trafficking and through mechanisms partially independent of microsomal triglyceride transfer protein–mediated ApoB lipidation. SEC16B partially localizes at ER-LD contact sites and promotes LD expansion by facilitating the targeting of ER proteins to LDs. More importantly, suppression of *Sec16b* dramatically lowers serum lipid levels and reduces atherosclerotic lesion size in *Ldlr* null mice. These data reveal a mechanism that coordinates VLDL and LD metabolism and suggest SEC16B as a potential therapeutic target for atherosclerosis treatment.

## Abstract 2

SEC16B helps the liver manage lipids by balancing their release and storage, and inhibiting it lowers blood lipids and reduces atherosclerosis, suggesting a promising treatment target.

## Graphical abstract

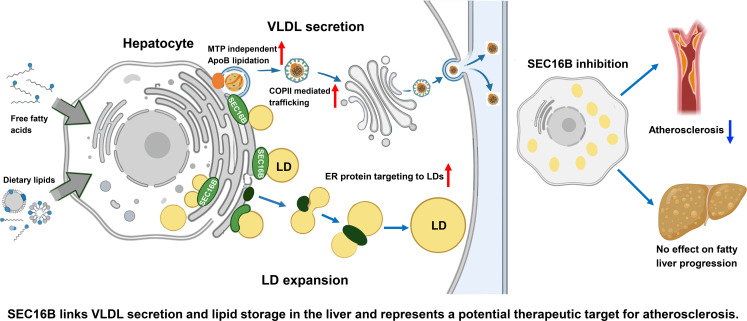

## Introduction

Dysregulation of lipid homeostasis is known to cause various diseases, including atherosclerosis and fatty liver diseases (1–3). Atherosclerosis, a chronic inflammatory disease, is triggered by the subendothelial retention of cholesterol-rich, apolipoprotein B–containing (ApoB-containing) lipoproteins within arterial walls. While elevated LDL cholesterol (LDL-C) is a well-established risk factor for atherosclerosis (4, 5), recent human genetic evidence also supports a causal role for elevated triglyceride-rich lipoproteins, such as VLDLs, in atherogenesis (2, 6). Although drugs targeting VLDL metabolism are available for treating hyperlipidemia, a substantial side effect is the accumulation of lipids in hepatocytes as lipid droplets (LDs), leading to fatty liver diseases (7). Thus, a better understanding of the regulation of VLDL and LD metabolism could open new avenues for the safe treatment of atherosclerosis.

The liver plays a pivotal role in maintaining systemic lipid homeostasis by regulating lipid de novo biosynthesis, lipid uptake from circulation derived from dietary sources or fatty acids released from adipose tissue during fasting, and the subsequent delivery of lipids to peripheral tissues in the form of VLDLs (8). During the early stages of VLDL biogenesis, lipids are incorporated into ApoB within the ER (9). This process, known as lipidation, is facilitated by the microsomal triacylglycerol transfer protein (MTP) (10). Following the initial ApoB lipidation, nascent VLDL undergoes additional bulk lipidation prior to exiting the ER to fuse with *cis*-Golgi. Its exit from the ER is considered to be the rate-limiting step in VLDL secretion (11). Due to its larger size, the egress of VLDL from the ER does not follow the canonical coat protein complex II–mediated (COPII-mediated) protein secretory pathway. Instead, its intracellular trafficking relies on specialized transport machinery known as VLDL transport vesicles, which require components of COPII machinery (12). However, the precise mechanisms controlling the lipidation and transport of VLDLs from the ER to Golgi have not been fully elucidated.

LDs are highly dynamic organelles that function as temporary lipid storage and act as a sink to alleviate cellular lipid stress under physiological conditions in the liver (13). An imbalance in the processes maintaining lipid homeostasis can result in LD formation, particularly when lipid influx or synthesis exceeds the liver’s capacity to secrete them as VLDLs. LD biogenesis is initiated by neutral lipid accumulation in the ER bilayer, leading to oil lens formation. The oil lens buds off and forms nascent LDs (14), which expand through local triglyceride (TG) synthesis and/or fusion with other smaller LDs (15, 16). Furthermore, ER-LD contacts facilitate lipid and protein exchange between ER and LDs, thereby sustaining LD growth (17). While the origins of VLDLs and LDs differ, they share a similar structure characterized by a hydrophobic core containing TGs and cholesterol esters, enclosed by a phospholipid monolayer. Previous studies have demonstrated a close interconnection between VLDL and LD metabolism. Impaired VLDL secretion leads to increased LD formation, whereas LDs serve as potential reservoirs of substrates for VLDL lipidation (9, 18, 19). However, the regulatory mechanisms governing the interplay between VLDL secretion and LD metabolism remain poorly understood.

In this study, we identify SEC16B as a regulator of both VLDL and LD metabolism. By analyzing GWAS datasets, we discovered significant associations between SNPs in the *SEC16B* locus and plasma cholesterol levels in humans. Both whole-body and liver-specific *Sec16b*-knockout (LKO) mice exhibit a substantial reduction in serum TG, total cholesterol, and free cholesterol levels due to impaired COPII-mediated VLDL intracellular trafficking and ApoB lipidation through mechanisms partially distinct from MTP-mediated initial lipidation. Surprisingly, despite higher hepatic lipid content, LKO mice show smaller LDs in hepatocytes. Mechanistically, SEC16B localizes to ER-LD contact sites, facilitating the targeting of LD-associated proteins that promote LD expansion. More importantly, loss of *Sec16b* ameliorates hyperlipidemia and reduces lesion areas in *Ldlr* null mice without causing apparent liver damage. These findings reveal a mechanism connecting VLDL and LD metabolism and demonstrate SEC16B as a potential therapeutic target for atherosclerosis.

## Results

### SNP in SEC16B is associated with plasma cholesterol levels in humans.

**Figure 1:**
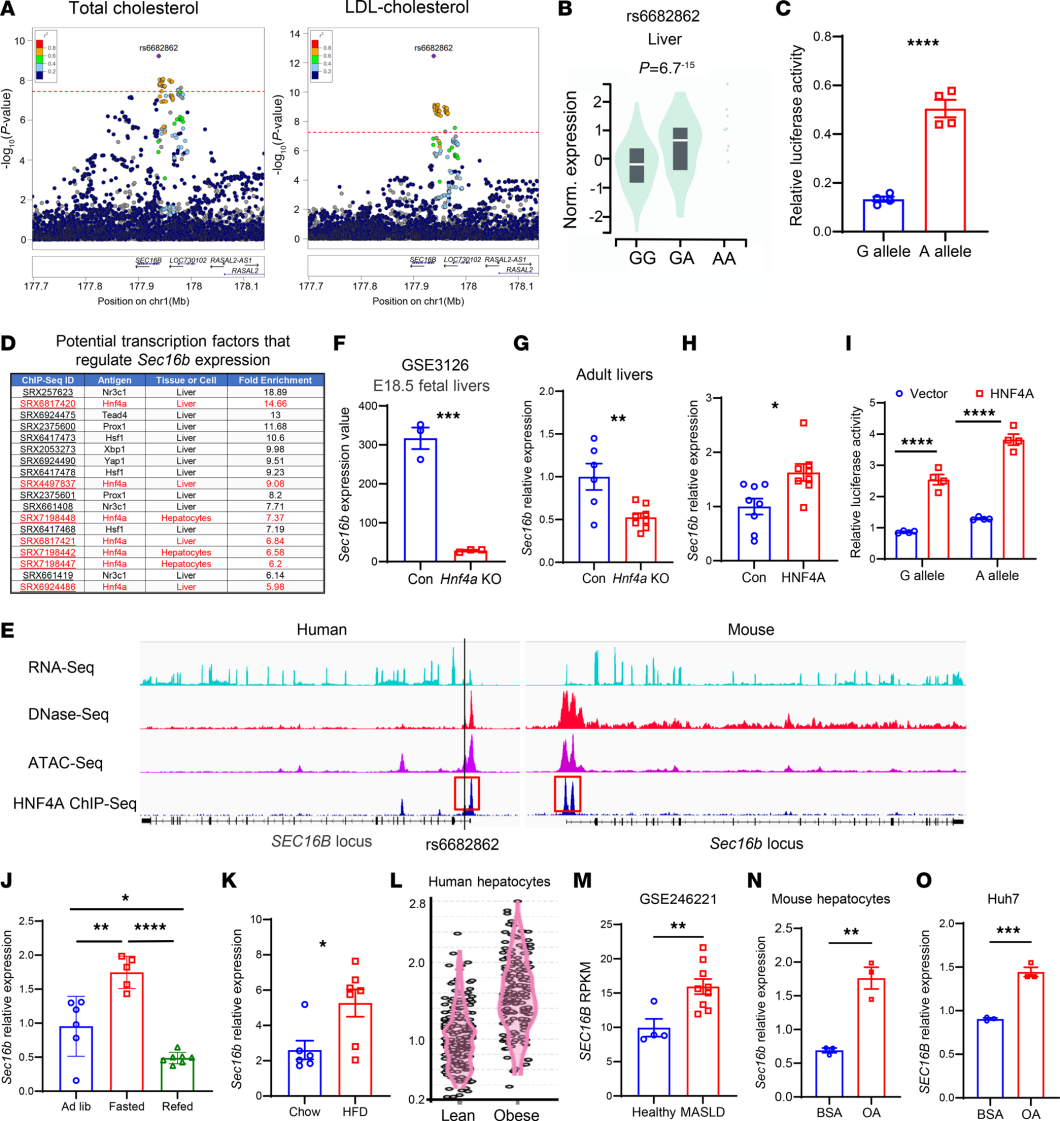
Association between SNPs in *SEC16B* gene and plasma cholesterol in humans and its expression regulation. (**A**) Regional plot of *SEC16B* associated with plasma total cholesterol and LDL-C levels in humans. Red dashed line indicates the threshold of genome-wide significance (*P* = 5 × 10^–8^). (**B**) eQTL studies showing a highly significant correlation between rs6682862 and *SEC16B* expression in the liver. (**C**) Luciferase activity of *SEC16B* promoter carrying G and A alleles in HEK293 cells (*n* = 4). (**D**) Enrichment analysis showing candidate transcription factors that may bind to 1,000 bp up- or downstream of *Sec16b* transcription start sites in mouse livers. Data are from ChIP-Atlas. (**E**) RNA-seq, DNase-seq, ATAC-seq, and HNF4A ChIP-seq reads at the *SEC16B* locus of human and mouse liver. Red boxes indicate HNF4A binding peaks from ChIP-seq data. (**F** and **G**) *Sec16b* mRNA levels in fetal (**F**) and adult male (**G**) *Hnf4a*-KO mouse livers (*n* = 3 in **F**; *n* = 6–8 in **G**). 8-week-old mice were fed an HFCF diet for 20 weeks (**G**). (**H**) *Sec16b* mRNA levels in male *HNF4A* overexpression mouse livers (*n* = 8). 8-week-old mice were fed an HFCF diet for 20 weeks. (**I**) Luciferase activity of *SEC16B* promoter carrying G and A alleles in control and *HNF4A*-overexpressing HEK293 cells (*n* = 4). (**J**) *Sec16b* mRNA levels in the livers of ad lib fed, fasted, and refed male mice (*n* = 5–7). 8-week-old mice were fasted for 12 hours or fasted for 12 hours and refed HCD for 12 hours. (**K**) *Sec16b* mRNA levels in the livers of 8 weeks old male mice fed chow or HFD for 12 weeks (*n* = 6–7). (**L**) *SEC16B* expression in lean and obese hepatocytes. Data are from Liver Cell Atlas. (**M**) *SEC16B* expression in the livers of healthy donors and patients with MASLD. (**N**) *Sec16b* mRNA levels in mouse primary hepatocytes treated with BSA and 200 μM oleic acid (OA). (**O**) *SEC16B* mRNA levels in Huh7 cells treated with BSA and 200 μM OA. Values are presented as mean ± SEM or as violin plots. Statistical analysis was performed with 2-tailed Student’s *t* test (**C**, **F**–**H**, **K**, and **M**–**O**), 1-way ANOVA (**J**), and 2-way ANOVA (**I**). *P < 0.05, **P < 0.01, ***P < 0.001, ****P < 0.0001.

GWAS has identified that 1 of the SNPs (rs6682862, chr1:177969302, alleles: G>A) located in the promoter region of *SEC16B* is significantly associated with plasma cholesterol levels (Figure 1A) (20, 21). An analysis of expression quantitative trait loci (eQTLs) revealed that the minor allele of rs6682862-A is correlated with increased expression of *SEC16B* in the liver (Figure 1B). To directly test whether different alleles affect *SEC16B* expression, we cloned the promoter region of human *SEC16B* genes (both G and A alleles) upstream of a firefly luciferase reporter gene. A luciferase assay demonstrated that the A allele increased luciferase activity by approximately 4-fold compared with the G allele (Figure 1C), which is consistent with eQTL data. Notably, DNase-seq and ATAC-seq analyses showed that rs6682862 resides within the open chromatin promoter region of the *SEC16B* gene on chromosome 1 (Figure 1E), further suggesting its regulatory role in *SEC16B* expression.

### Hepatic SEC16B expression is regulated by HNF4A and feeding conditions.

Using enrichment analysis in ChIP-Atlas (22), we identified HNF4A as a potential transcription factor that binds to the promoter region of *SEC16B* (Figure 1D). An analysis of the ChIP sequencing dataset for HNF4A in the liver revealed 2 binding peaks in the promoter region of the *SEC16B* gene (Figure 1E). It is noteworthy that HNF4A also binds to the promoter of the mouse *Sec16b* gene (Figure 1E). Examination of a published microarray dataset from *Hnf4a*-knockout fetal livers showed a substantial reduction in *Sec16b* expression in the absence of HNF4A (Figure 1F) (23). In line with this, *Sec16b* expression was also downregulated by AAV-TBG-CRE–mediated *Hnf4a* acute knockout in the livers of *Hnf4a-*floxed mice fed a high-fat/cholesterol/fructose (HFCF) diet containing 40% fat, 0.2% cholesterol, and 4.2% fructose (Figure 1G). Conversely, adeno-associated virus–mediated (AAV-mediated) overexpression of *HNF4A* markedly increased *Sec16b* expression in the livers of mice on the HFCF diet (Figure 1H). Furthermore, *HNF4A* overexpression markedly enhanced luciferase activity in both A and G allele constructs (Figure 1I). These findings demonstrate that HNF4A regulates *SEC16B* expression in vitro and in vivo.

Previous studies have shown that HNF4A plays a critical role in maintaining lipid homeostasis in the liver (24, 25). Given the strong association between *SEC16B* polymorphism and plasma cholesterol levels, *SEC16B* may function as a downstream target of HNF4A that mediates its effects on lipid metabolism. Gene expression profiling revealed that *SEC16B* is highly expressed in the livers of both humans and mice (Supplemental Figure 1, A and B; supplemental material available online with this article; https://doi.org/10.1172/JCI204602DS1), the central organ for lipid homeostasis. Furthermore, we found that hepatic *Sec16b* expression was upregulated by fasting and high-fat diet (HFD) feeding (Figure 1, J and K), conditions known to promote lipid accumulation in the liver. Consistently, hepatocytes from obese individuals, who are susceptible to steatosis, expressed higher levels of *SEC16B* (Figure 1L) (26). Similarly, *SEC16B* expression was also elevated in the livers of patients with metabolic dysfunction–associated steatotic liver disease (MASLD) (Figure 1M). Moreover, fatty acid treatment markedly increased *SEC16B* expression in both mouse hepatocytes and Huh7 cells (Figure 1, N and O). Collectively, these data indicate that SEC16B is responsive to lipid accumulation and likely plays a key role in hepatic lipid metabolism.

### Sec16b deficiency decreases serum lipids in mice.

**Figure 2:**
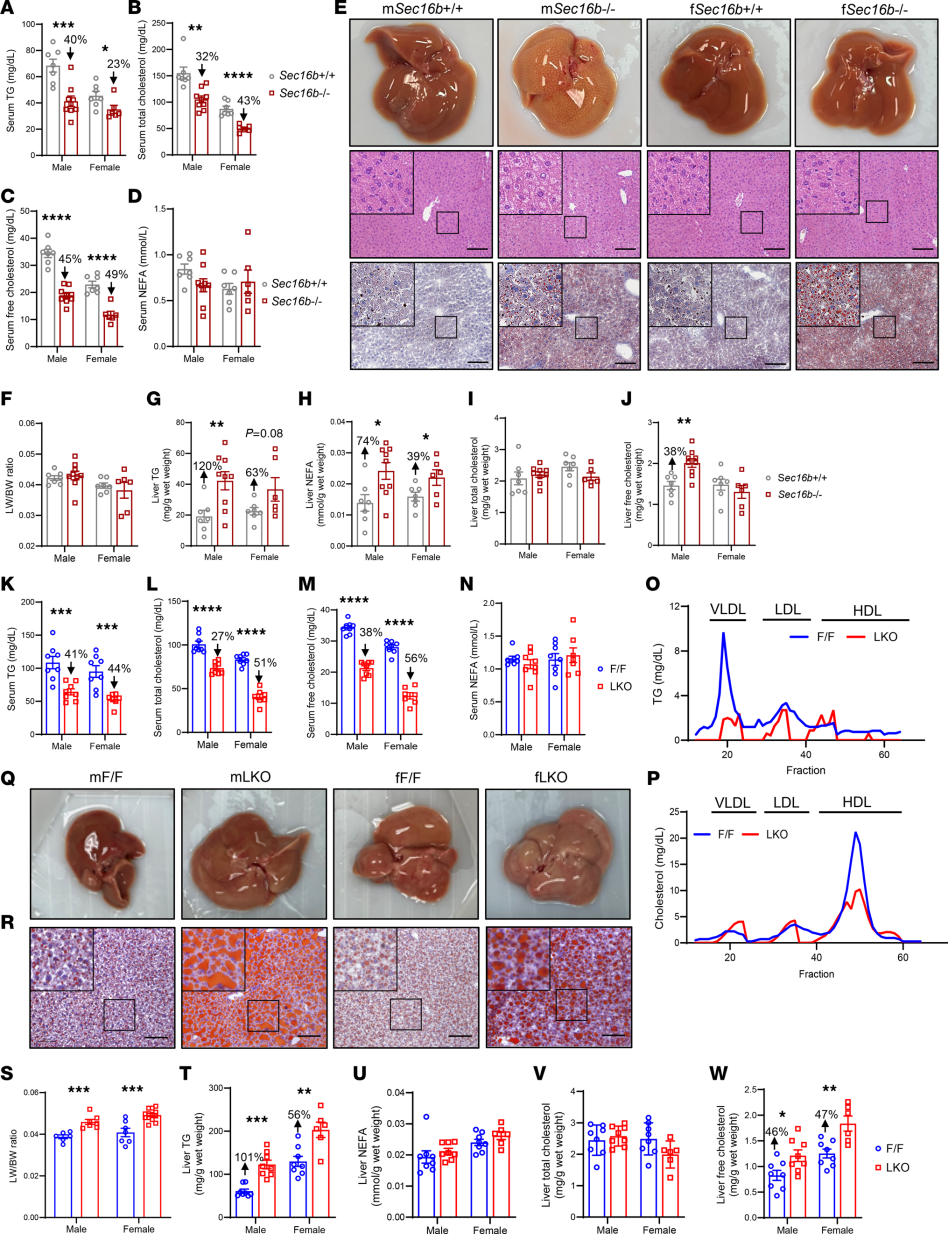
Loss of *Sec16b* reduces serum lipids and leads to hepatic lipid accumulation. (**A**–**D**) Serum lipid levels in *Sec16b*^+/+^ and *Sec16b*^–/–^ mice after 6 hours of fasting (*n* = 6–9). (**E**) Representative gross images and H&E and Oil Red O staining of livers from *Sec16b^+/+^* and *Sec16b*^–/–^ mice fasted for 6 hours (*n* = 3). Scale bars: 100 μm. (**F**) Liver/body weight ratio of *Sec16b^+/+^* and *Sec16b*^–/–^ mice fasted for 6 hours (*n* = 6–9). LW, liver weight. (**G**–**J**) Hepatic lipid levels of *Sec16b^+/+^* and *Sec16b*^–/–^ mice fasted for 6 hours (*n* = 6–9). (**K**–**N**) Serum lipid levels in control (*Sec16b*^fl/fl^ [F/F]) and LKO (*Sec16b*^fl/fl^, *Albumin*-Cre) mice after 16 hours of fasting (*n* = 7–8). (**O** and **P**) FPLC analysis of lipoprotein profiles of serum from male control (F/F) and LKO mice fasted for 16 hours. Serum from 7 mice/group was pooled. (**Q** and **R**) Representative gross images (**Q**) and Oil Red O staining (**R**) of livers from control (F/F) and LKO mice fasted for 16 hours. Scale bars: 100 μm. (**S**–**W**) Liver/body weight ratio and hepatic lipid levels of control (F/F) and LKO mice fasted for 16 hours (*n* = 6–13). Values are presented as mean ± SEM. Statistical analysis was performed with 2-tailed Student’s *t* test. *P < 0.05, **P < 0.01, ***P < 0.001, ****P < 0.0001.

To investigate the function of SEC16B in vivo, we generated *Sec16b* whole-body knockout (*Sec16b*^–/–^) mice by crossing heterozygous *Sec16b*^tm1a(KOMP)Wtsi^ (*Sec16b*^+*/*–^) mice. Real-time RT-PCR analysis confirmed a more than 95% reduction in *Sec16b* mRNA level in the livers, small intestines, subcutaneous white adipose tissues, and muscles of *Sec16b*^–/–^ mice (Supplemental Figure 1C). *Sec16b*^–/–^ mice were viable, fertile, and normal in appearance. Lipid measurement revealed a significant approximately 25%–50% reduction in serum TG, cholesterol, and free cholesterol levels in both male and female *Sec16b*^–/–^ mice compared with controls after 6 hours of fasting (Figure 2, A–D), while serum nonesterified fatty acid (NEFA) levels were comparable between groups. Although there was no difference in liver/body weight ratio (Figure 2F), gross appearance, histological analysis, and Oil Red O staining indicated increased lipid accumulation in *Sec16b*^–/–^ mice (Figure 2E). This was supported by approximately 40%–120% increase in hepatic TG, NEFA, and free cholesterol levels (Figure 2, G–J), likely due to reduced serum lipid levels.

Given that *Sec16b* is highly expressed in the mouse and human livers (Supplemental Figure 1, A and B), we crossed *Sec16b-*floxed mice with *Albumin*-Cre mice to generate liver-specific knockout (*Sec16b*^fl/fl^, *Albumin*-Cre [LKO]) mice. *Sec16b* mRNA was almost completely depleted in the livers of LKO mice, while the expression of its homolog *Sec16a* remained unaffected (Supplemental Figure 1D). Similar to whole-body knockout mice, both male and female LKO mice exhibited much lower serum TG and total and free cholesterol levels (~30%–55%), but not NEFA levels compared with control floxed mice after overnight fasting (Figure 2, K–N). Fast protein liquid chromatography (FPLC) analysis of pooled serum from control and LKO mice showed that hepatic *Sec16b* deletion markedly reduced TG level in the VLDL fraction and cholesterol level in the HDL fraction (Figure 2, O and P). The gross examination revealed changes consistent with lipid accumulation, including pale livers and a higher liver/body weight ratio in LKO mice (Figure 2, Q and S). Lipid accumulation was further confirmed by Oil Red O staining and direct quantification of hepatic lipid content (~45%–100%) (Figure 2, R and T–W). Additionally, lipidomics analysis of livers from overnight fasted control and LKO mice corroborated the increases in TG and diacylglycerol levels but not phospholipids and ceramide (Supplemental Figure 2, A–L). Similar phenotypes were also observed in LKO mice fasted for 6 hours (Supplemental Figure 1, E–H).

### SEC16B modulates VLDL lipidation and secretion.

**Figure 3:**
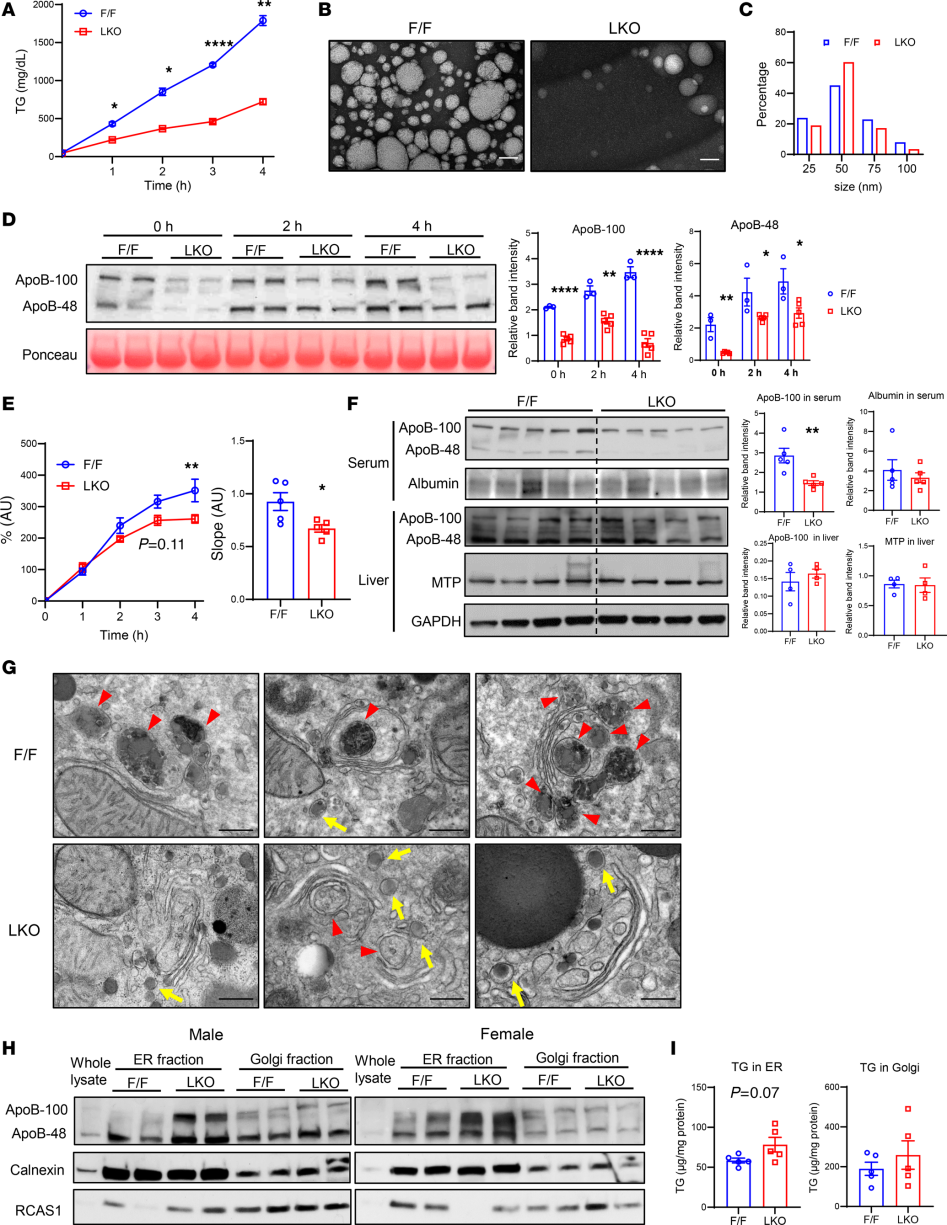
Hepatic *Sec16b* deficiency impairs ApoB lipidation and VLDL secretion. (**A**) VLDL-TG secretion in control (F/F) and LKO female mice. Mice were fasted for 4 hours followed by Tyloxapol injection. Serum TG levels were measured at indicated time points (*n* = 3–5). (**B** and **C**) Negative staining and quantification of serum VLDL particle size in control (F/F) and LKO mice as in A. Scale bars: 100 nm. (**D**) Representative Western blot image and quantification of serum ApoB levels in control (F/F) and LKO mice as in **A**. (**E**) Quantification of ^35^S-labeled ApoB-100 secretion rates in Tyloxapol-injected male and female control (F/F) and LKO mice (*n* = 5/group). (**F**) Western blot analysis and quantification of ApoB, albumin, and MTP in the serum and livers of male control (F/F) and LKO mice after 16 hours of fasting. (**G**) Electron microscopy analysis of liver sections from male control (F/F) and LKO mice after 16 hours of fasting (*n* = 3). Red arrowheads indicate VLDL secretory vesicles. Yellow arrows indicate LDs in the ER. Scale bars: 400 nm. (**H**) Western blot analysis of ApoB in whole lysate, ER, and Golgi fractions of livers from control (F/F) and LKO mice after 16 hours of fasting. (**I**) TG levels in the ER and Golgi fractions of livers from male control (F/F) and LKO mice fasted for 16 hours (*n* = 5). Values are presented as mean ± SEM. Statistical analysis was performed with 2-tailed Student’s *t* test (**D**, **E** [slope], **F**, and **I**) and 2-way ANOVA (**A** and **E**). *P < 0.05, **P < 0.01, ****P < 0.0001.

The marked reduction in serum lipid levels and hepatic lipid accumulation in LKO mice led us to hypothesize that loss of *Sec16b* may hinder VLDL secretion from the liver. To directly assess VLDL secretion, we retro-orbitally injected control and LKO mice with Tyloxapol, which blocks VLDL clearance by inhibiting lipoprotein lipase activity and TG hydrolysis. Remarkably, LKO mice showed an approximately 60% reduction in TG secretion (Figure 3A). This decrease in TG output can result from compromised ApoB lipidation and/or impaired VLDL secretion. Electron microscopy of negatively stained VLDL fractions revealed much smaller VLDL particles in LKO mice (Figure 3, B and C), indicating impaired ApoB lipidation. Western blot analysis revealed a reduction in serum ApoB levels in both Tyloxapol-injected and 16 hour fasted LKO mice compared with controls (Figure 3, D and F). To directly quantify ApoB secretion rates, we performed an ^35^S labeling assay in vivo, which demonstrated that loss of *Sec16b* reduced ApoB secretion by approximately 25% relative to controls (Figure 3E). Together, these data indicate that hepatic ApoB secretion is impaired in the absence of SEC16B. In contrast, serum albumin levels were comparable between control and LKO (Figure 3F). Moreover, Coomassie blue and silver staining of serum proteins revealed that most proteins were comparable between the 2 groups (Supplemental Figure 3A), indicating that SEC16B is unlikely to play a major role in overall protein secretion. Interestingly, despite the reduced secretion of ApoB into serum, ApoB protein and mRNA levels remained unchanged in LKO livers (Figure 3F and Supplemental Figure 3, B and C).

To assess the relevance to humans, we knocked down *SEC16B* in Huh7 human liver cells using shRNA. Consistent with our mouse data, *SEC16B* knockdown reduced ApoB secretion into the medium by approximately 50% (Supplemental Figure 4A), concomitant with ApoB accumulation in cell lysate, suggesting that SEC16B also regulates ApoB secretion in humans.

ApoB undergoes 2 stages of lipidation: an initial lipidation facilitated by MTP and a poorly understood bulk lipidation (9). We next investigated whether *Sec16b* deficiency affects the abundance and function of MTP. Western blot and real-time RT-PCR analysis showed no difference in MTP protein and mRNA levels in control and LKO livers (Figure 3F and Supplemental Figure 3, B and C). Direct measurement of neutral lipid transfer activity from microsomes isolated from mouse livers and Huh7 cells revealed no significant differences between groups (Supplemental Figure 4, B–D). Additionally, *SEC16B* knockdown reduced ApoB secretion to a similar extent in both DMSO and MTP inhibitor Lomitapide-treated Huh7 cells (Supplemental Figure 4A). Interestingly, ApoB accumulated in DMSO-treated but not Lomitapide-treated cells (Supplemental Figure 4A), likely because ApoB and MTP expression was decreased in Lomitapide-treated *SEC16B*-knockdown cells (Supplemental Figure 4, E and F). To further determine whether MTP mediates the ApoB secretion defect caused by *SEC16B* deficiency, we overexpressed MTP in control and *SEC16B-*knockdown cells and assessed ApoB secretion. MTP overexpression enhanced ApoB secretion to a comparable level in both control and knockdown cells (Supplemental Figure 4G). Together, these findings suggest that SEC16B likely regulates ApoB secretion in a manner at least partially distinct from MTP-mediated initial lipidation.

To gain further insight into the mechanism underlying defective VLDL secretion in *Sec16b*-LKO mice, we conducted electron microscopy analysis on control and LKO liver samples. In control hepatocytes, lipoprotein particles were easily visualized in the secretory vesicles and Golgi (red arrowheads, Figure 3G). In contrast, lipoprotein particles were much smaller and less stained in the secretory vesicles of LKO hepatocytes, further corroborating poor ApoB lipidation in the absence of SEC16B. Notably, we did not detect prominent lipoprotein particles in the Golgi of LKO hepatocytes. Instead, an increase in ER luminal LDs was observed in LKO hepatocytes (yellow arrows, Figure 3G). To further investigate if SEC16B regulates the intracellular trafficking of VLDL particles, we isolated ER and Golgi fractions from control and LKO livers and analyzed ApoB and TG levels in them. Interestingly, we found increased levels of ApoB-100 in the ER fractions of LKO livers (Figure 3H), suggesting that loss of *Sec16b* likely blocks the trafficking of ApoB from ER to Golgi. Meanwhile, we observed a trend toward a modest increase in TG content in the ER fractions of LKO livers (Figure 3I). These data demonstrate that SEC16B likely regulates VLDL production by facilitating both ApoB lipidation and trafficking to the Golgi in hepatocytes.

### Knockdown of SEC16B impairs the interaction between SEC13 and SEC31A, thereby disrupting COPII assembly and ApoB secretion.

VLDL intracellular trafficking is mediated by specialized COPII-coated vesicles that are assembled at designated ER exit sites (ERESs) (27). To investigate the molecular mechanisms by which SEC16B regulates VLDL secretion, we measured the protein levels of COPII components in LKO livers and *SEC16B* knockdown Huh7 cells. Notably, most of these proteins were upregulated in LKO livers and *SEC16B*-knockdown Huh7 cells (Supplemental Figure 5, A–C). Consistent with its localization to the ERES (28), SEC16B showed almost complete colocalization with COPII components, including SEC31A, SEC23A, and SEC24B, in Huh7 cells (Supplemental Figure 5, D–F). Interestingly, SEC13 only partially colocalized with SEC16B or other COPII components (Supplemental Figure 5, D–F), likely because SEC13 is also a component of the nuclear pore complex and the GATOR complex (29–31).

**Figure 4:**
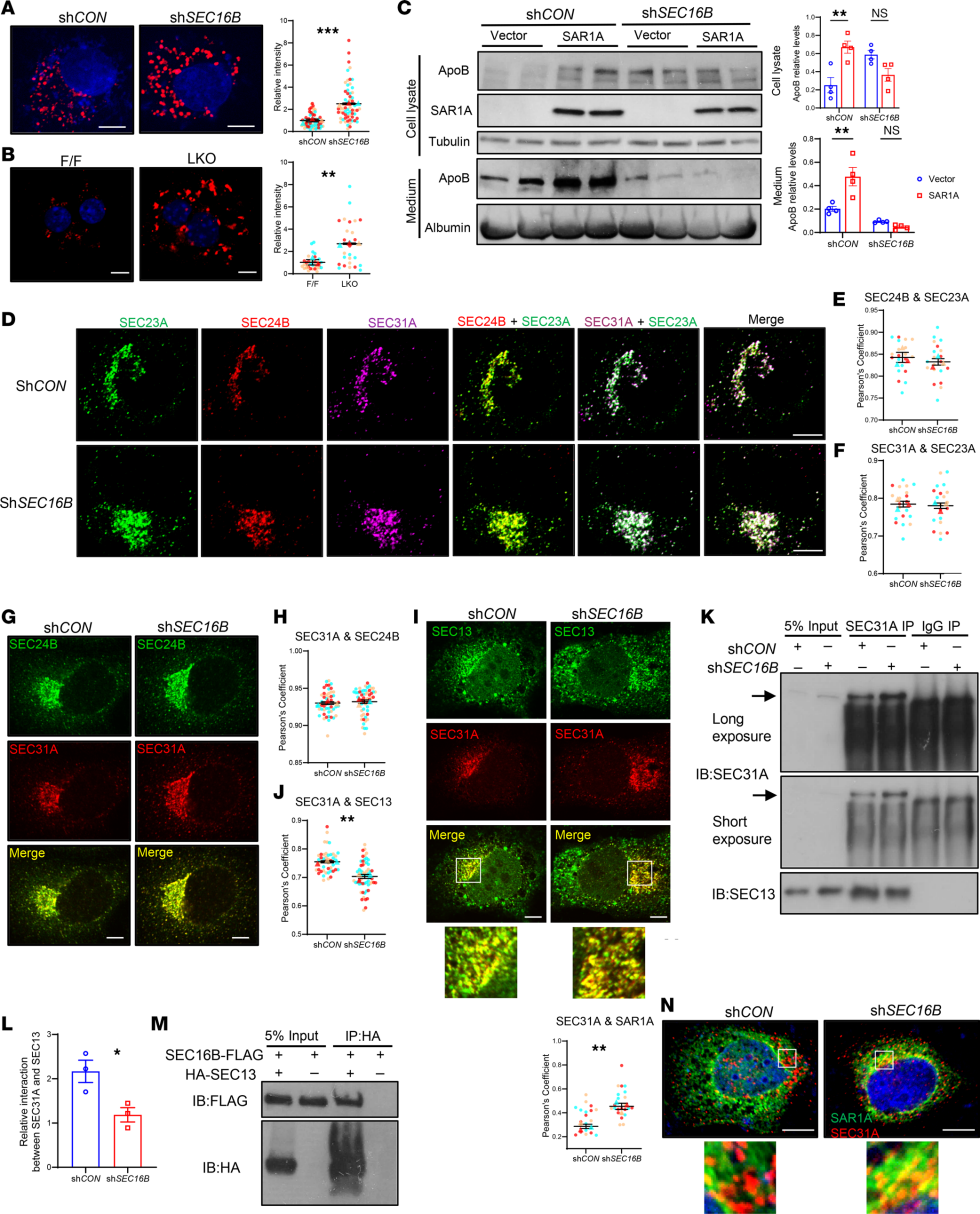
SEC16B modulates the interaction between SEC13 and SEC31A, thereby promoting ApoB secretion. (**A**) Immunostaining and quantification of relative intensity of SAR1-GTP (red) in sh*CON* and sh*SEC16B* Huh7 cells cultured in DMEM+OA for 6 hours. Scale bars: 8 μm. (**B**) Immunostaining and quantification of relative intensity of SAR1-GTP (red) in F/F and LKO primary hepatocytes cultured in maintenance medium. Scale bars: 8 μm. (**C**) Representative Western blot images and quantification of ApoB levels in the cell lysate and medium of sh*CON* and sh*SEC16B* Huh7 cells cultured in DMEM+OA for 16 hours. Cells were transfected with vector control or SAR1A-HA (*n* = 4). (**D**–**F**) Colocalization (**D**) and Pearson’s coefficients (**E** and **F**) of endogenous SEC31A (purple), SEC24B (red), and exogenous SEC23A (green) in sh*CON* and sh*SEC16B* Huh7 cells transfected with eGFP-SEC23A and cultured in DMEM+OA for 6 hours (*n* = 3). Scale bars: 8 μm. (**G** and **H**) Colocalization (**G**) and Pearson’s coefficients (**H**) of endogenous SEC24B (green) and SEC31A (red) in sh*CON* and sh*SEC16B* Huh7 cells cultured in DMEM+OA for 6 hours (*n* = 3). Scale bars: 8 μm. (**I** and **J**) Colocalization (**I**) and Pearson’s coefficients (**J**) of endogenous SEC13 (green) and SEC31A (red) in sh*CON* and sh*SEC16B* Huh7 cells cultured in DMEM+OA for 6 hours (*n* = 3). Scale bars: 8 μm. (**K** and **L**) Co-IP (**K**) and quantification (**L**) of SEC31A interaction with SEC13 in sh*CON* and sh*SEC16B* Huh7 cells cultured in DMEM+OA for 6 hours. The relative interaction levels between SEC31A and SEC13 were determined by normalizing the SEC13 signal to the SEC31A signal within the immunocomplex (*n* = 3). (**M**) SEC16B-FLAG and HA-SEC13 co-IP assay in HEK293T cells (*n* = 3). (**N**) Colocalization and Pearson’s coefficients of exogenous SAR1A (green) and endogenous SEC31A (red) in sh*CON* and sh*SEC16B* Huh7 cells transfected with SAR1A-HA and cultured in DMEM+OA for 6 hours (*n* = 3). Scale bars: 8 μm. Values are presented as mean ± SEM or as violin plots. Statistical analysis was performed with 2-way ANOVA (**C**) or 2-tailed Student’s *t* test (**A**, **B**, **E**, **F**, **H**, **J**, **L**, and **N**). *P < 0.05, ***P < 0.001, ****P < 0.0001.

COPII complex assembly is initiated by the activation of SAR1, which transitions from a GDP-bound to a GTP-bound state (32, 33). Immunofluorescence analysis using an antibody specific for the GTP-bound SAR1 revealed significantly elevated SAR1-GTP levels in *SEC16B*-knockdown Huh7 cells and LKO hepatocytes (Figure 4, A and B), indicating enhanced initiation of COPII vesicle assembly. To further investigate the involvement of SEC16B in SAR1 and COPII-mediated VLDL secretion, we attempted to overexpress SAR1 in Huh7 cells. Of the 2 SAR1 paralogs, SAR1B is well established as a key mediator of lipoprotein secretion (34), whereas SAR1A shares partially overlapping functions in COPII-mediated lipoprotein secretion (34, 35). Interestingly, only SAR1A, but not SAR1B, could be robustly overexpressed in Huh7 cells (data not shown). Nevertheless, SAR1A overexpression significantly increased ApoB production and secretion in control Huh7 cells (Figure 4C). In contrast, *SEC16B* knockdown completely abolished the SAR1A-induced increase in ApoB secretion (Figure 4C), indicating that SEC16B is essential for COPII-mediated VLDL trafficking.

Upon activation, SAR1 recruits SEC23 and SEC24 to form the inner layer of the COPII coat, followed by the assembly of the outer layer composed of SEC13 and SEC31 (33). *SEC16B* knockdown did not affect the association between SEC24B and SEC23A, nor the colocalization between the inner and outer coat components, including SEC31A with SEC24B, and SEC31A with SEC23A (Figure 4, D–H). In contrast, *SEC16B* knockdown impaired the association and interaction between the outer coat components SEC13 and SEC31A (Figure 4, I–L). In yeast, SEC16 interacts with SEC13 through its central conserved domain (36, 37). Given that SEC16B shares a similar central conserved domain with yeast SEC16 (28), we hypothesized that SEC16B may interact with SEC13 in mammals. Indeed, co-IP analysis revealed an interaction between SEC16B and SEC13, but not with SAR1A or SEC23A (Figure 4M and Supplemental Figure 5, G and H). These data suggest that SEC16B selectively modulates the assembly of the COPII outer coat complex, likely by interacting with SEC13.

Once the inner and outer COPII coats are fully assembled, SAR1-GTP is hydrolyzed by SEC23, a process accelerated by the outer layer complex (38). This GTP hydrolysis is a crucial step for COPII vesicle scission and release. Our observations of impaired outer coat assembly and elevated SAR1-GTP levels in *SEC16B*-knockdown cells suggest that COPII vesicle release may be compromised in the absence of SEC16B. Consistent with this, the association between SEC31A and SAR1A was markedly increased in *SEC16B*-knockdown Huh7 cells (Figure 4N), indicating defective vesicle release. To directly visualize COPII dynamics, we performed time-lapse microscopy to track the movement of GFP-SEC13–labeled puncta in control and *SEC16B*-knockdown cells (Supplemental Videos 1–6). Quantitative analysis of the puncta trajectories revealed significant reduction in mean and maximal speed and displacement, as well as a trend toward decreased total travel distances in *SEC16B-*knockdown cells (Supplemental Figure 6, A–D). Furthermore, the proportion of moving puncta was also significantly reduced in knockdown cells (Supplemental Figure 6, E and F). Taken together, these findings suggest that SEC16B promotes VLDL secretion by modulating COPII assembly and its intracellular trafficking, potentially through mediating the interaction between SEC31A and SEC13.

### SEC16B controls hepatic LD expansion.

**Figure 5:**
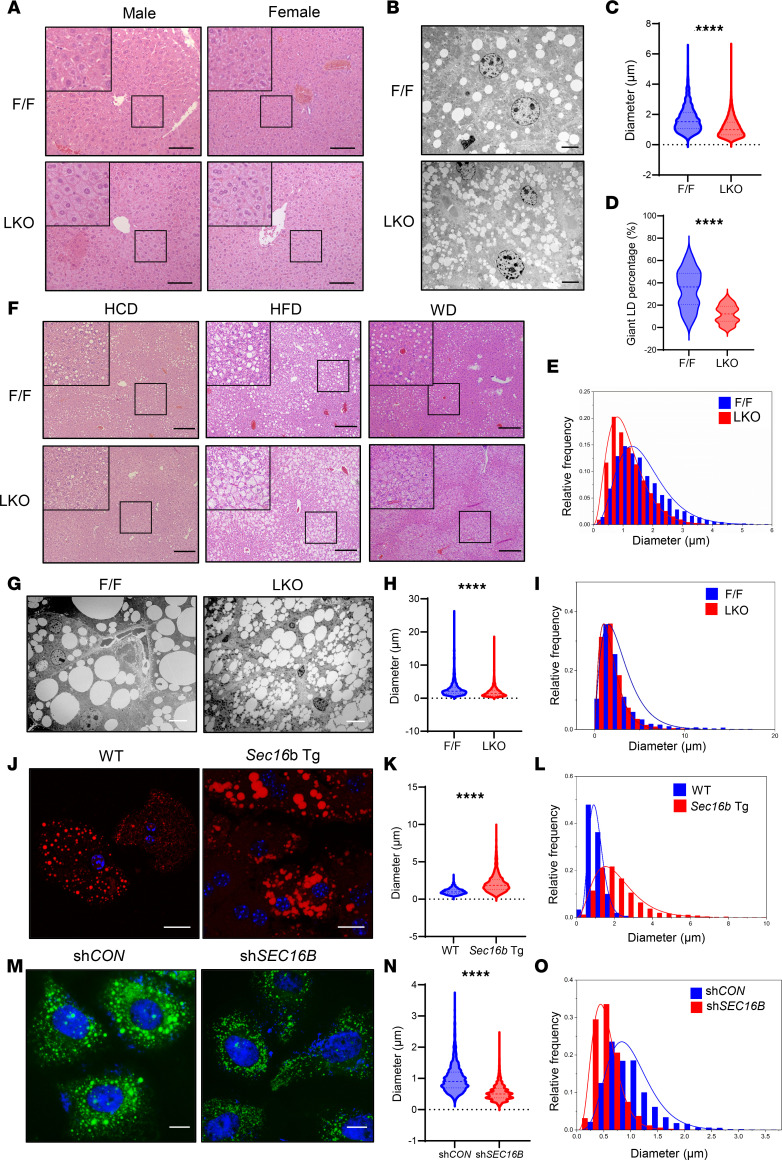
SEC16B controls LD expansion in the liver. (**A**) Representative H&E staining of livers from control (F/F) and LKO mice fasted for 16 hours (*n* = 4). Scale bars: 100 μm. (**B** and **C**) Representative electron microscopy (EM) images and quantification of LD diameter in the livers of male control (F/F) and LKO mice fasted for 16 hours (*n* = 3). Scale bars: 6 μm. (**D** and **E**) Percentage of giant LDs (diameter > 2 μm) and the relative frequency of LD size in the livers of male control (F/F) and LKO mice fasted for 16 hours. (**F**) Representative H&E staining of livers from 8-week-old male control (F/F) and LKO mice fed an HFD, HCD, and WD for 6–12 weeks (*n* = 3). Scale bars: 200 μm. (**G**–**I**) Representative EM images, quantification, and relative frequency of LD size in the livers of WD-fed male control (F/F) and LKO mice (*n* = 3). Scale bars: 8 μm. (**J**–**L**) Representative images, quantification, and relative frequency of LD size in male WT and *Sec16b* transgenic (Tg) primary hepatocytes treated with 200 μM OA (*n* = 3). Scale bars: 20 μm. (**M**–**O**) Representative images, quantification, and relative frequency of LD size in sh*CON* and sh*SEC16B* Huh7 cells treated with 200 μM OA (*n* = 3). Scale bars: 10 μm. Values are presented as mean ± SEM or as violin plots. Statistical analysis was performed with 2-tailed Student’s *t* test (**D**) or Mann-Whitney test (**C**, **H**, **K**, and **N**). ****P < 0.0001.

Impaired VLDL secretion typically leads to LD formation and steatosis in hepatocytes. Histological analysis revealed characteristics of microvesicular steatosis in the livers of both male and female LKO mice after overnight fasting, as manifested by distended hepatocytes with central nuclei and white, foamy-appearing cytoplasm (Figure 5A). Microvesicular steatosis tends to have small LDs, leading us to consider whether *Sec16b* deletion affects LD size. Electron microscopy analysis and quantification of LD size revealed that LKO hepatocytes indeed contained a greater number of smaller LDs than controls (Figure 5, B–E).

Next, we challenged the mice with different diets known to promote lipid accumulation in the liver, including high carbohydrate diet (HCD), HFD, and Western diet (WD). Interestingly, chronic dietary challenges had minimal impacts on serum or hepatic TG or NEFA levels (Supplemental Figure 7, A–C), likely due to their dynamic regulation by different organs, such as liver, intestine, and adipose tissues. In contrast, serum cholesterol levels remained significantly reduced in LKO mice fed these diets (Supplemental Figure 7, A–C). Histological analysis showed that LKO livers exhibited prominent microvesicular steatosis without noticeable LDs (Figure 5F). Electron microscopy analysis of WD-fed mouse livers revealed much smaller LDs in LKO mice compared with control mice (Figure 5, G–I). Conversely, overexpression of *Sec16b* in mouse primary hepatocytes increased LD size (Figure 5, J–L). To further investigate whether SEC16B regulates LD size in humans, we knocked down *SEC16B* expression in Huh7 cells and observed significantly smaller LDs upon oleic acid treatment (Figure 5, M–O). These data indicate that SEC16B is both necessary and sufficient to modulate LD size in the liver.

### SEC16B partially localizes to ER-LD contact sites and controls protein targeting to LDs.

LDs are classified into 3 types based on their subcellular localization: cytoplasmic, nuclear, and ER luminal LDs (13). To examine if SEC16B affects a specific subset of LDs, we stained for PLIN2, a marker of cytoplasmic LDs, in *SEC16B*-knockdown Huh7 cells and LKO hepatocytes. The results showed that most LDs were PLIN2 positive in both control and knockdown or LKO cells, suggesting that the majority of LDs are cytoplasmic and that *Sec16b* deficiency does not alter PLIN2 localization (Supplemental Figure 8, A and B).

The size of cytoplasmic LDs can be regulated by various processes, including lipid biosynthesis/uptake, TG/VLDL secretion, and LD catabolism and growth (13, 39). Considering that *Sec16b* deficiency impairs VLDL secretion, which would theoretically lead to increased lipid accumulation and larger LD formation, the presence of smaller LDs in LKO hepatocytes is unlikely caused by impaired VLDL secretion. Gene expression analysis showed no significant differences in the expression of SREBP-1c and its downstream lipogenic genes or lipid hydrolysis genes after overnight fasting (Supplemental Figure 3, B–D), suggesting that these pathways are unlikely to account for the smaller LDs in the absence of SEC16B. Similarly, the expression of most known genes involved in LD expansion, such as cell death inducing DFFA like effector (*Cide*) family members, diacylglycerol *O*-acyltransferase 2 (*Dgat2*), glycerol 3-phosphate acyltransferase 4 (*Gpat4*), and acyl-CoA synthetase long-chain family member 3 (*Acsl3*), was not significantly altered between control and LKO livers, except for a modest change in *Cideb* and Calsyntenin 3β (*Clstn3b*) mRNA levels in male LKO mice (Supplemental Figure 3, B and C). Western blot analysis of whole liver lysates showed no change in GPAT4 protein levels and a trend toward slightly decreased DGAT2 and CIDEB protein levels in LKO livers (Supplemental Figure 8D). These data suggest that the effect of *Sec16b* deficiency on LD expansion is unlikely to be driven by altered gene expression.

**Figure 6:**
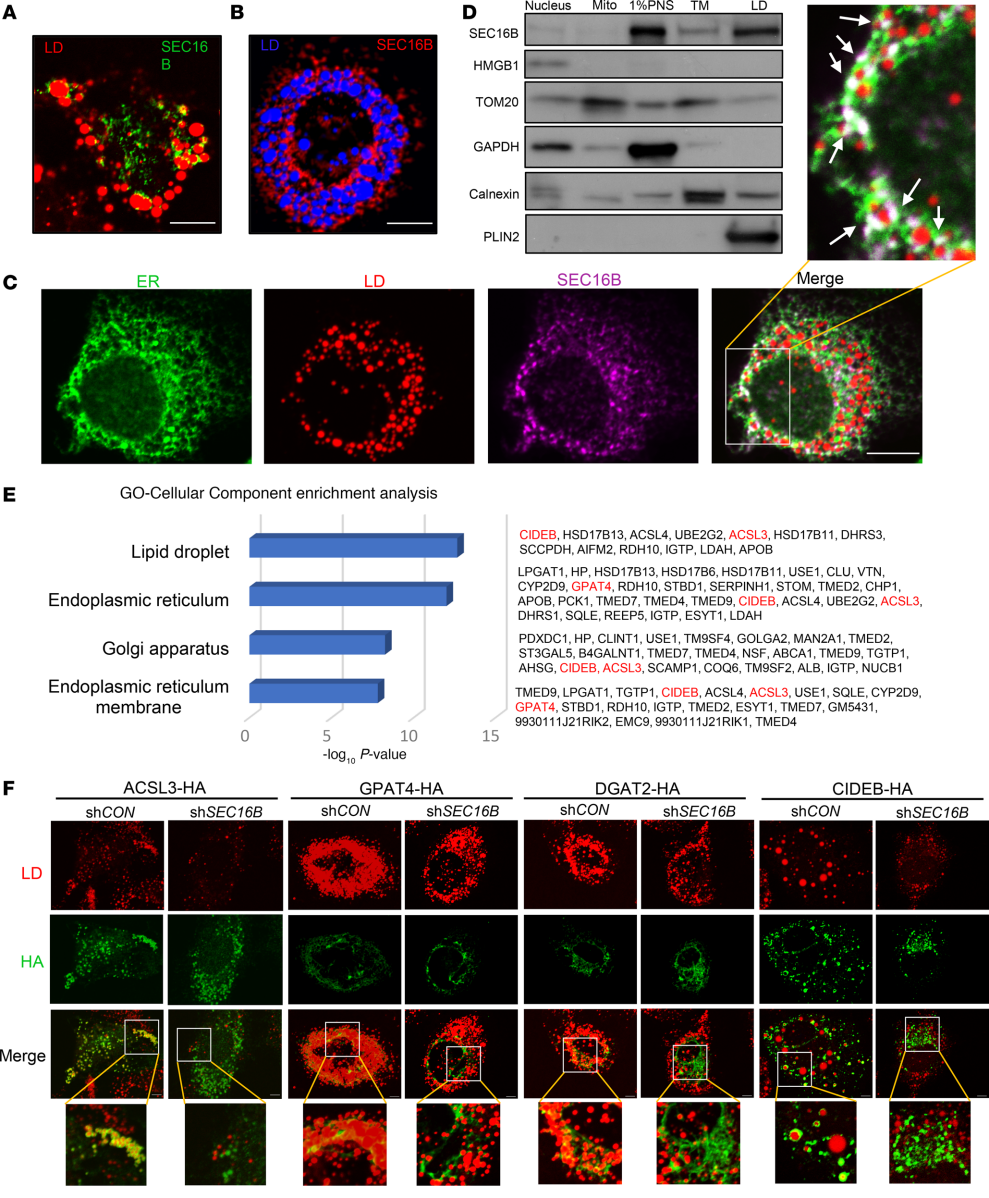
SEC16B localizes to ER-LD contact sites and controls protein targeting to LDs. (**A**) Confocal microscopy image of 6-hour OA-treated Huh7 cells transfected with SEC16B-FLAG (green) and costained with LDs (red) (*n* = 3). (**B**) Confocal microscopy image of 16-hour OA-treated Huh7 cells transfected with SEC16B-FLAG (red) and costained with LDs (blue) (*n* = 3). (**C**) Confocal microscopy images of 6-hour OA-treated Huh7 cells transfected with SEC16B-FLAG (purple) and costained with LDs (red) and KDEL (green) (*n* = 3). Scale bars: 10 μm (**A**–**C**). (**D**) Western blot analysis of subcellular fractions from the livers of *Sec16b-3XFLAG*-transgenic male mice. (**E**) Gene Ontology–Cellular Component enrichment analysis of downregulated proteins on LDs from male LKO mice compared with controls. Proteins labeled in red have been reported to localize to both ER and LDs and are associated with LD size (*n* = 4–6). (**F**) Confocal microscopy analysis of 6-hour OA-treated sh*CON* and sh*SEC16B* Huh7 cells transfected with HA-tagged ACSL3, GPAT4, DGAT2, and CIDEB (green) and costained with LDs (red) (*n* = 3). Original magnification, ×2.5.

To further elucidate the mechanism by which SEC16B controls LD size, we first examined the subcellular localization of SEC16B. Immunofluorescence staining revealed that SEC16B was partially localized to the surface of LDs in Huh7 cells (Figure 6, A and B). Notably, costaining with the ER marker KDEL and LDs showed that some SEC16B signals were localized to the contact sites between the ER and LDs (Figure 6C). Additionally, SEC16B partially overlapped with PLIN2 surrounding LDs (Supplemental Figure 8C). To validate this observation, we fractionated liver samples from FLAG-tagged *Sec16b* transgenic mice. Western blot analysis demonstrated the enrichment of SEC16B in both LD and total membrane fractions with high ER content, as indicated by ER membrane marker CALNEXIN (Figure 6D). Given that ER-LD contact sites mediate protein and lipid transfer between the organelles (13, 40–42), we hypothesized that SEC16B localizes to ER-LD contact sites and facilitates the translocation of LD-associated proteins to LDs, thereby modulating LD expansion. Indeed, silver staining revealed that protein levels normalized to TG content were reduced in LDs purified from LKO livers compared with controls (Supplemental Figure 8E). Interestingly, Western blot analysis showed that CIDEB, but not PLIN2 or CALNEXIN, was reduced in LDs from LKO livers normalized to either TG content or total proteins (Supplemental Figure 8, F and G), suggesting that SEC16B may facilitate the translocation of selected proteins to LDs.

Next, we performed proteomics analysis on LDs isolated from control and LKO mouse livers. As expected, the majority of significantly downregulated proteins were associated with LDs and/or ER (Figure 6E). Remarkably, proteins such as ACSL3, GPAT4, DGAT2, and CIDEB have been previously reported to translocate to LDs and promote their expansion (15, 43, 44). To visualize if SEC16B regulates their translocation, we overexpressed these proteins tagged with HA in control and *SEC16B-*knockdown Huh7 cells and performed immunofluorescence analysis upon oleic acid treatment. Knockdown of *SEC16B* dramatically reduced the colocalization of these proteins with LDs (Figure 6F). Taken together, these findings demonstrated that SEC16B is a regulator of LD expansion through selectively modulating the translocation of LD-associated proteins.

### Constitutive Sec16b knockout ameliorates atherosclerosis.

**Figure 7:**
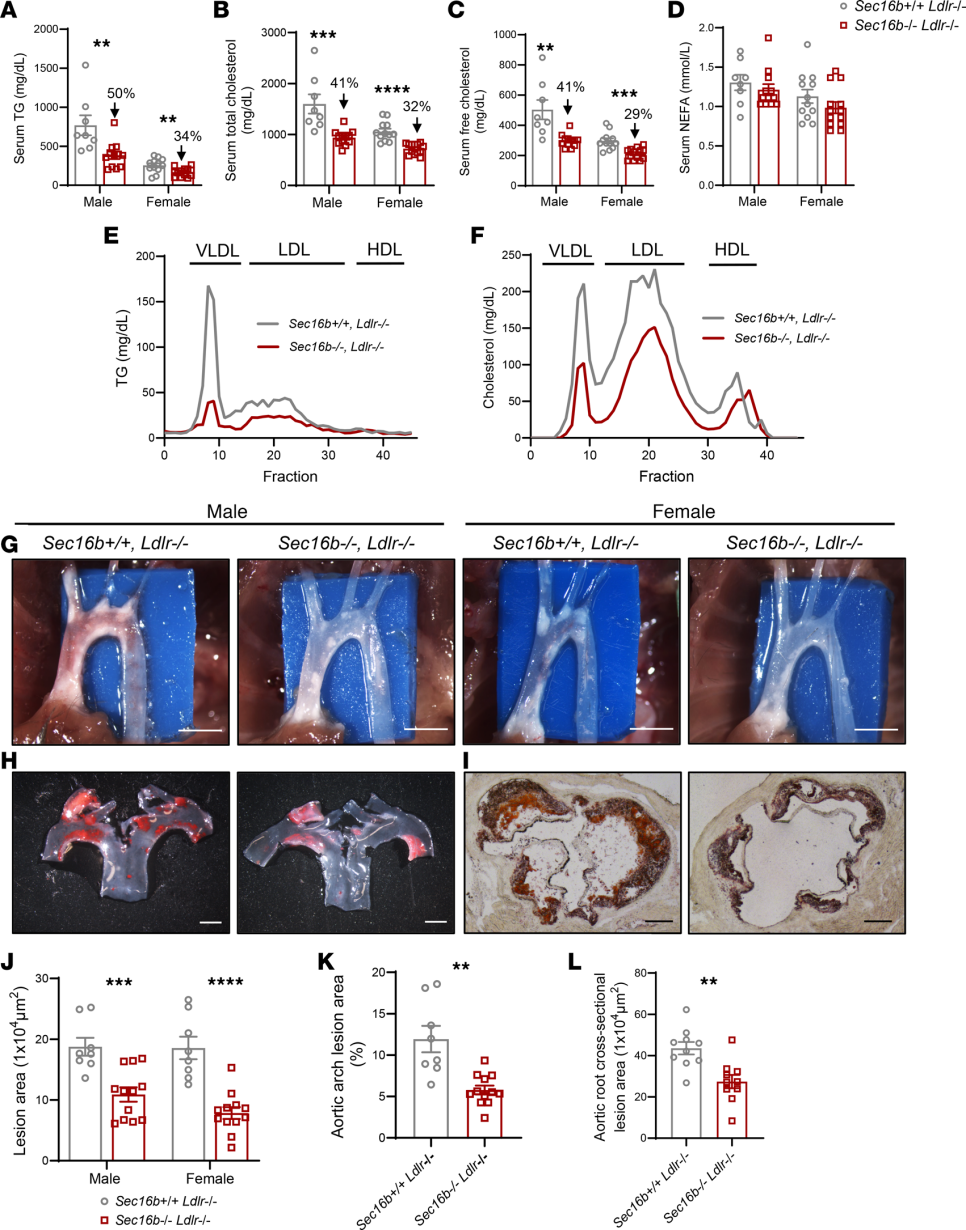
Whole-body *Sec16b* knockout protects against hyperlipidemia and atherosclerosis in *Ldlr* null mice. (**A**–**D**) Serum lipid levels of 8-week-old *Sec16b^+/+^ Ldlr^–/–^* and *Sec16b^–/–^ Ldlr^–/–^* mice fed a WD for 12 weeks (*n* = 8–13). (**E** and **F**) FPLC analysis of lipoprotein profiles of serum from 8-week-old male *Sec16b^+/+^ Ldlr^–/–^* and *Sec16b^–/–^ Ldlr^–/–^* mice fed a WD for 12 weeks. Serum from 7 mice/group was pooled. (**G**–**I**) Representative images of aorta (**G**), en face aortic arch (**H**), and aortic root section (**I**) from 8-week-old *Sec16b^+/+^ Ldlr^–/–^* and *Sec16b^–/–^ Ldlr^–/–^* mice fed a WD for 12 weeks (*n* = 8–12 in **G** and **H**; *n* = 10 in **I**). Scale bars: 1 mm (**G** and **H**), 200 μm (**I**). (**J**–**L**) Quantifications of aortic lesion areas in **G**–**I**. Values are presented as mean ± SEM. Statistical analysis was performed with 2-tailed Student’s *t* test. **P < 0.01, ***P < 0.001, ****P < 0.0001.

Genetic, epidemiologic, and clinical studies have established that hyperlipidemia, particularly elevated LDL-C, is a key risk factor for atherosclerosis (6, 45, 46). Given that *Sec16b* deficiency markedly reduces serum lipid levels, we investigated whether loss of *Sec16b* affects atherogenesis. To induce atherosclerosis in mice, we crossed *Sec16b*^–/–^ mice with *Ldlr*^–/–^ mice to generate *Sec16b*^+/+^ *Ldlr*^–/–^ (control) and *Sec16b*^–/–^ *Ldlr*^–/–^ (DKO) mice and fed them a WD for 12 weeks. Both male and female mice in the control and DKO groups exhibited similar blood glucose levels and liver/body weight ratios (Supplemental Figure 9, B and C). Body weight was comparable in male mice, whereas female DKO mice showed a trend toward increased body weight (*P* = 0.053) (Supplemental Figure 9A). As expected, deletion of *Ldlr* combined with WD feeding led to an approximately 10-fold increase in serum TG, cholesterol, and free cholesterol levels. Notably, serum TG and total and free cholesterol levels were significantly reduced by approximately 30%–50% in both male and female DKO mice compared with control mice, while NEFA levels remained comparable between the groups (Figure 7, A–D). FPLC analysis revealed a dramatic reduction in VLDL-TG, as well as VLDL-C and LDL-C, in DKO serum compared with controls (Figure 7, E and F). These data also suggest that *Sec16b* deficiency–induced serum lipid reduction is independent of LDLR-mediated lipid uptake.

The evaluation of atherosclerotic plaques revealed much smaller aortic lesion area in both male and female DKO mice than controls (Figure 7, G and J). The reduction in plaque area was further confirmed by Oil Red O staining of the en face aortic arch (Figure 7, H and K) and aortic root sections (Figure 7, I and L). A potential concern with reducing hyperlipidemia by blocking VLDL secretion is the risk of lipid accumulation in the liver, which could lead to MASLD. Indeed, DKO mice exhibited an approximately 60% increase in hepatic TG compared with control mice, while other lipid levels were not altered (Supplemental Figure 9, F–I). Despite more microvesicular steatosis in DKO livers from histological analysis (Supplemental Figure 9D), similar levels of inflammation and fibrosis were observed when compared with control mice (Supplemental Figure 9, D and E). Consistently, the mRNA levels of inflammatory genes (*Tnfa*, *F4/80*, and *Il1b*) and fibrogenic genes (*Col1a1*, *Col3a1*, and *Tgfb*) were not altered in DKO mice (Supplemental Figure 9J). Furthermore, no increases in alanine transaminase (ALT) and aspartate transaminase (AST) levels were detected in DKO mice compared with controls (Supplemental Figure 9, K and L). Thus, *Sec16b* deficiency ameliorates atherosclerosis progression without causing noticeable lipotoxicity in the liver.

### Acute deletion of hepatic Sec16b delays atherosclerosis progression.

**Figure 8:**
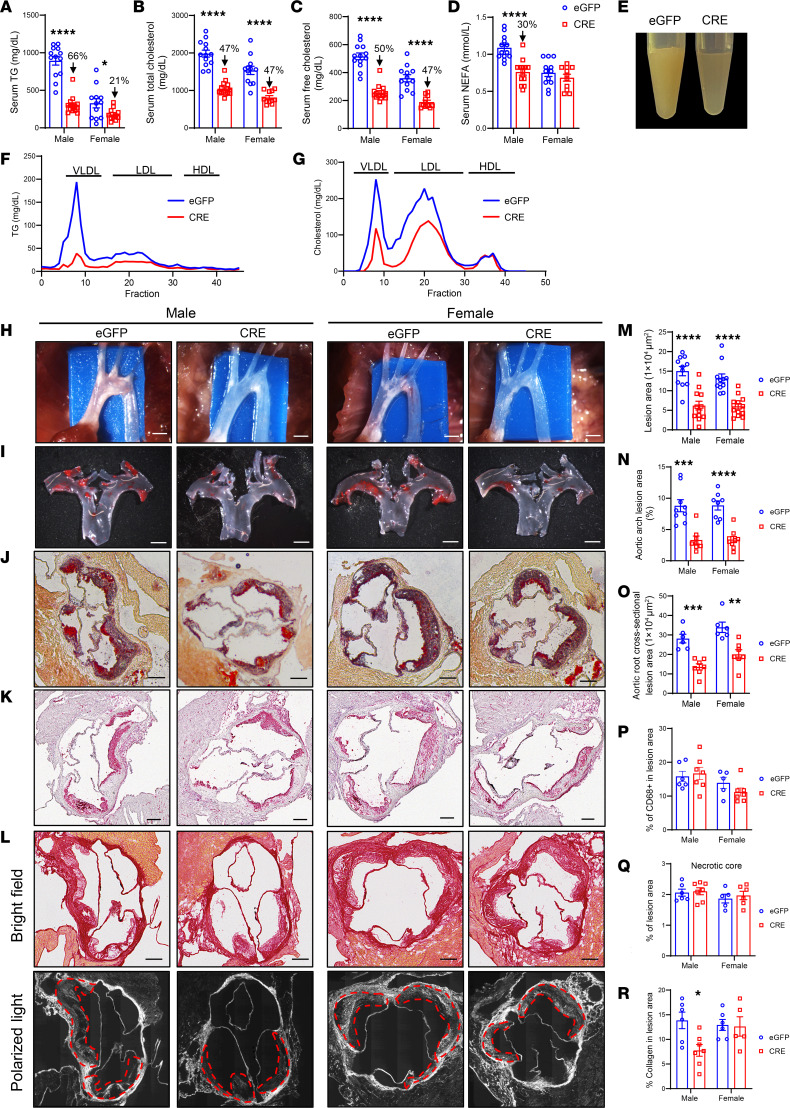
Hepatic *Sec16b* deficiency ameliorates hyperlipidemia and atherosclerosis in *Ldlr-*null mice. (**A**–**D**) Serum lipid levels in 12-week WD-fed *Sec16b*^fl/fl^ (F/F) *Ldlr^–/–^* mice receiving eGFP or CRE AAV (*n* = 11–14). (**E**) Gross image of serum from 12-week WD-fed male *Sec16b*^fl/fl^ (F/F) *Ldlr^–/–^* mice receiving eGFP or CRE AAV. (**F** and **G**) FPLC analysis of lipoprotein profiles in serum from 12-week WD-fed male *Sec16b*^fl/fl^ (F/F) *Ldlr^–/–^* mice receiving eGFP and CRE AAV. Serum from 7 mice/group was pooled. (**H**–**J**) Representative images of aorta (**H**), en face aortic arch (**I**), and aortic root section (**J**) from 12-week WD-fed *Sec16b*^fl/fl^ (F/F) *Ldlr^–/–^* mice receiving eGFP or CRE AAV (*n* = 11–13 in **H**; *n* = 8–9 in **I**; *n* = 6–7 in **J**). Scale bars: 1 mm (**H** and **I**), 200 μm (**J**). (**K** and **L**) Representative images of CD68 immunostaining (**K**) and Sirius Red staining (**L**) of aortic root section from 12-week WD-fed *Sec16b*^F/F^ (F/F) *Ldlr^–/–^* mice receiving eGFP or CRE AAV (*n* = 5–7). Scale bars: 200 μm. (**M**–**R**) Quantification of aortic lesion areas in **H**–**J** and percentage of CD68^+^ macrophages, necrotic core area, and collagen content in aortic root lesions in **K** and **L**. Values are presented as mean ± SEM. Statistical analysis was performed with 2-tailed Student’s *t* test. *P < 0.05, **P < 0.01, ***P < 0.001, ****P < 0.0001.

To investigate whether acute deletion of *Sec16b* can mitigate atherosclerosis development and serve as a therapeutic strategy, we crossed *Sec16b*^fl/fl^ mice with *Ldlr*^–/–^ mice to generate *Sec16b*^fl/fl^ *Ldlr*^–/–^ mice. These mice were fed a WD for 6 weeks to initiate atherosclerosis development, followed by retro-orbital injection with either eGFP or CRE AAV driven by a liver-specific TBG promoter and then continued on the WD for an additional 6 weeks (Supplemental Figure 10A). No significant differences in body weight or blood glucose levels were observed between eGFP AAV– and CRE AAV–injected mice, regardless of sex (Supplemental Figure 10, B and C). Both male and female mice receiving CRE AAV exhibited approximately 20%–60% reductions in serum TG and total and free cholesterol levels compared with those injected with eGFP (Figure 8, A–C). In contrast, NEFA levels were only reduced in male mice (Figure 8D). Consistently, serum from CRE AAV–injected mice appeared relatively clear compared with the more milky serum of eGFP AAV–injected mice (Figure 8E). FPLC analysis revealed markedly lower TG and cholesterol levels in the VLDL and LDL fractions of CRE AAV–treated mice (Figure 8, F and G).

Aortic lesion areas were dramatically reduced in both male and female mice receiving CRE AAV, as evidenced by direct measurement of plaque area (Figure 8, H and M) and Oil Red O staining of the en face aortic arch (Figure 8, I and N) and aortic root sections (Figure 8, J and O). Next, we analyzed plaque composition by assessing macrophage and collagen contents. CD68 immunostaining revealed that the percentage of macrophage area relative to the lesion area was comparable between eGFP and CRE AAV–injected mice (Figure 8, K and P). Sirius Red staining demonstrated reduced collagen content in the lesions of CRE AAV–injected male mice, but not in female mice (Figure 8, L and R). Quantification of the necrotic core area, an indicator of plaque stability, showed no difference between eGFP- and CRE-injected mice, indicating comparable plaque stability between groups (Figure 8Q). Moreover, the necrotic cores comprised a relatively small fraction of the lesion area (~2%), suggesting an early stage of atherosclerosis. These data demonstrated that plaque composition remains largely unchanged between groups, suggesting that hepatic *Sec16b* deficiency reduces plaque burden without modulating the inflammatory status of the atherosclerotic lesion.

Next, we assessed the effect of acute *Sec16b* deletion on the liver. The liver/body weight ratio was slightly increased in male CRE AAV–injected mice but not in females (Supplemental Figure 10D). Lipid analysis showed elevated hepatic TG but no changes in other lipids in both male and female CRE AAV–treated mice (Supplemental Figure 10, E–H). H&E staining revealed more microvesicular steatosis in both male and female CRE AAV–injected mice (Supplemental Figure 10K), while eGFP AAV–injected mice exhibited macrovesicular steatosis. Additionally, male mice receiving CRE AAV showed reduced inflammation (Supplemental Figure 10K), which was further supported by decreased mRNA levels of inflammatory genes (*Tnfa* and *Il1b*) (Supplemental Figure 10I). Interestingly, the mRNA levels of fibrogenic genes (*Col1a1* and *Col3a1*) were downregulated in female CRE AAV–injected mice (Supplemental Figure 10J). Moreover, there was no discernible difference in fibrosis between CRE AAV– and eGFP AAV–injected mice of either sex, as assessed by Sirius Red staining (Supplemental Figure 10L). In agreement with the absence of noticeable liver damage, there was no difference in ALT and AST levels between eGFP AAV– and CRE AAV–injected mice (Supplemental Figure 10, M and N). Consistent with the observations in LKO mice, *Sec16b* knockout also reduced LD size in the livers of *Ldlr*^–/–^ mice (Supplemental Figure 10, O and P). These data suggest that SEC16B may represent a potential therapeutic target for hyperlipidemia and atherosclerosis.

### Hepatic Sec16b deletion does not affect disease progression in mouse models of metabolic dysfunction–associated steatohepatitis.

To further investigate whether *Sec16b* deficiency influences the progression of MASLD or metabolic dysfunction–associated steatohepatitis (MASH), we fed *Sec16b*-LKO mice a MASH-inducing diet that has been shown to closely recapitulate human MASH in mouse livers (47, 48). After 24 weeks of diet feeding, severe steatosis but only mild fibrosis were observed (Supplemental Figure 11, A and B). More pronounced fibrosis was developed in mice subjected to 50 weeks of diet feeding or 24 weeks of MASH diet feeding combined with CCI4 treatment. However, regardless of the feeding regimen or CCI4 treatment, both control and LKO mice exhibited similar levels of steatosis, fibrosis, and inflammation in the liver (Supplemental Figure 11, A and B). Moreover, the mRNA expression of inflammatory genes (*Tnfa*, *F4/80*, and *Il1b*) and fibrogenic genes (*Col1a1*, *Col3a1*, and *Tgfb*) remained unchanged in LKO mice compared with controls (Supplemental Figure 11C). Consistent with other chronic dietary challenges, serum or hepatic TG or NEFA levels were not altered in LKO mice upon MASH diet feeding (Supplemental Figure 11D). Interestingly, serum cholesterol levels were significantly reduced in LKO mice only after 24 weeks of MASH diet feeding (Supplemental Figure 11D), likely due to severe liver injury and impaired hepatic function caused by prolonged MASH diet exposure. Nevertheless, liver enzyme activities (ALT and AST) were found to be comparable between control and LKO mice (Supplemental Figure 11E). These findings demonstrated that SEC16B is unlikely to play a substantial role in the development of MASLD and MASH.

## Discussion

Excess lipids, particularly free fatty acids and cholesterol, are known to cause cellular lipotoxicity. In hepatocytes, free fatty acids and cholesterol can be esterified into TG and cholesterol esters, respectively, which are either stored in LDs or secreted into circulation as VLDLs. These tightly regulated processes help prevent hepatic lipid stress and meet the lipid demands of peripheral tissues. However, the mechanism underlying this regulation remains unclear. Through GWAS on human plasma lipids, we identified SEC16B as a vital regulator of both VLDL and LD metabolism. Our data demonstrate that hepatic *Sec16b* deficiency impairs VLDL lipidation and secretion, as well as LD expansion, suggesting that VLDL and LD metabolism may rely on the same machinery in the liver. Notably, hepatic *Sec16b* expression is induced by lipid accumulation in mice subjected to overnight fasting and chronic HFD feeding, as well as in humans that are obese or have MASLD. These findings demonstrate that SEC16B functions as a lipid-responsive regulator in the liver that promotes VLDL secretion and LD expansion, thereby handling lipid flux and maintaining lipid homeostasis. Interestingly, our results indicate that *SEC16B* is a target of HNF4A, one of the most abundant transcription factors and a pivotal regulator of lipid metabolism in the liver (24). This likely accounts for the predominant expression of *Sec16b* in both human and mouse liver and suggests that SEC16B may partially mediate the roles of HNF4A in regulating hepatic lipid metabolism.

VLDL metabolism involves several key steps, including ApoB translation, lipidation, trafficking from the ER to Golgi, and secretion into circulation. MTP plays a crucial role in transferring neutral lipids onto ApoB, forming nascent VLDL particles. Recent studies have identified several factors that modulate MTP activity and thereby influence ApoB lipidation, such as PLA2G12B (49) and PRAP1 (50). It is known that disruption of MTP function reduces ApoB lipidation, leading to its degradation (51, 52). Our in vivo analyses in mouse liver, along with in vitro experiments in Huh7 cells, suggest that *Sec16b* deficiency does not affect MTP expression or its function. Interestingly, despite the poor lipidation of ApoB in *Sec16b*-knockout livers, there is an increased accumulation of ApoB in the ER, which appears to contradict the prevailing notion that unlipidated ApoB is destined for degradation (53, 54). However, this observation aligns with the idea that MTP primarily participates in the initial lipidation of ApoB rather than in the subsequent bulk lipid addition (9, 55, 56). Given the intact MTP activity in the absence of SEC16B, it is possible that partially lipidated ApoB is stabilized in the ER, thereby avoiding degradation. This suggests that SEC16B is primarily involved in the relatively less understood process of bulk lipidation.

SEC16B is the shorter mammalian ortholog of *Saccharomyces* *cerevisiae* SEC16, initially recognized as a scaffold protein that organizes ERES through interactions with COPII components (57, 58). In mammalian cells, most research has been focused on SEC16A, the longer ortholog, which is ubiquitously expressed and shares the most similarity with yeast SEC16 (28). Although both SEC16A and SEC16B participate in COPII vesicle assembly and ERES organization, several in vitro studies indicate that SEC16B likely has specialized, nonredundant functions (28, 59). While VLDL and protein secretion both depend on COPII vesicles (60), recent studies indicate that VLDL secretion is segregated from general protein secretion upon exiting the ER (61). Indeed, our findings indicate that loss of *Sec16b* in mouse liver does not affect circulating levels of albumin, the most abundant serum protein secreted by the liver, or the levels of most other serum proteins, indicating that SEC16B is not essential for general protein secretion. Thus, SEC16A may play a more prominent role in COPII-mediated protein secretion in the liver, while SEC16B appears to have a specialized function in regulating lipoprotein secretion. Mechanistically, we found that SEC16B interacts with SEC13 and facilitates the interaction between SEC31A and SEC13, thereby promoting the assembly of the outer layer of the COPII coat and facilitating VLDL trafficking from the ER to Golgi.

Strikingly, our data demonstrate that SEC16B also regulates LD morphology at ER-LD contact sites, which are believed to facilitate the transfer of proteins and lipids. Since LDs contribute to VLDL lipidation and secretion (9, 18, 19, 39), it is plausible that SEC16B may influence LD-associated VLDL lipidation. SEC16B likely facilitates the localization of proteins, such as ACSL3, GPAT4, DGAT2, and CIDEB, to LDs, thereby mediating the lipid transfer between LDs and VLDL. Notably, deficiency in *Dgat2* or *Cideb* has been shown to reduce serum lipids and impair VLDL secretion (19, 62, 63). Whether the mislocalization of these or other yet unidentified proteins contributes to the impaired VLDL lipidation in *Sec16b*-deficient mice remains to be determined. Nevertheless, further investigation is warranted to elucidate this SEC16B-mediated VLDL bulk lipidation process involving LDs.

The biogenesis and growth of LDs are dynamically regulated in hepatocytes to maintain lipid homeostasis. Our data demonstrate that *Sec16b* deficiency leads to the accumulation of a greater number of smaller LDs, indicating that SEC16B primarily influences LD expansion rather than biogenesis. LD expansion is mediated by LD-associated proteins that either catalyze local TG synthesis or facilitate the fusion of smaller LDs. Specifically, GPAT4 and DGAT2 catalyze TG synthesis on the LD surface, which is considered a crucial step in LD growth (15). Additionally, smaller LDs can fuse to form large LDs through the action of CIDE family proteins (64). These proteins, originally located in the ER, translocate to LDs to mediate expansion. However, the precise mechanism governing their translocation remains elusive. Our studies indicate that SEC16B plays a critical role in regulating their translocation. Notably, a genome-wide screen in *Drosophila* identified components of the ERES — including SEC16, the only homolog of SEC16 in *Drosophila* — as key players in ER-to-LD protein targeting (65). Thus, the role of SEC16 and ERES in LD protein translocation appears to be conserved.

Another important finding of our study is the identification of SEC16B as a potential therapeutic target for atherosclerosis. Recent studies have established VLDL, the precursor lipoprotein of atherogenic TG-rich lipoprotein remnants and LDL, as a significant risk factor for atherosclerotic cardiovascular disease (ASCVD) (66, 67). Therefore, targeting VLDL metabolism has emerged as a potential strategy to alleviate hyperlipidemia and reduce ASCVD risk. However, a key limitation of suppressing VLDL secretion is the risk of hepatic lipid accumulation, which could progress to MASLD and MASH. Indeed, Lomitapide and mipomersen, an MTP inhibitor and ApoB synthesis inhibitor, respectively, have been approved only for patients with homozygous familial hypercholesterolemia due to their adverse effects of hepatic fat accumulation and liver injury (7). In this study, we found that targeting SEC16B may offer a safe strategy to mitigate hyperlipidemia without causing overt adverse effects. Under chronic western, high-fat, or high-carbohydrate diet feeding conditions, *Sec16b-*LKO mice exhibited no or minimal hepatic lipid accumulation while maintaining significant reduction in serum cholesterol levels. This likely reflects the more complex interplay among different tissues in TG metabolism compared with cholesterol metabolism. Alternatively, *Sec16b*-deficient livers may be less efficient in secreting lipids via VLDL in response to acute influx of massive lipids from adipose tissue during fasting, whereas LKO mice may have developed compensatory mechanisms to handle hepatic TG in response to chronic feeding. Notably, *Ldlr*^–/–^ mice lacking *Sec16b* exhibited increased hepatic lipid accumulation. Interestingly, control *Ldlr*^–/–^ mice (*Sec16b*^+/+^ *Ldlr*^–/–^ mice or AAV-TBG-eGFP–injected *Ldlr*^–/–^ mice) showed much higher TG levels (~200 mg/g liver weight in Supplemental Figure 9F and Supplemental Figure 10E) compared with WT mice after 12 weeks of WD feeding (~100 mg/g liver weight in Supplemental Figure 7C), indicating that *Ldlr*^–/–^ itself causes a markedly greater lipid burden on the liver, which is consistent with previous observations (68). It is therefore plausible that compensatory mechanisms in *Sec16b-*deficient mice are insufficient to cope with this excessive lipid load in the *Ldlr*^–/–^ background. This may explain why *Sec16b*^–/–^ *Ldlr*^–/–^ or AAV-TBG-CRE–injected *Ldlr*^–/–^ mice exhibit higher TG compared with their respective controls. Importantly, despite increased hepatic lipid accumulation in *Ldlr*^–/–^ mice lacking *Sec16b*, we did not observe any apparent liver damage, and the expression of certain inflammatory or fibrogenic genes was even reduced in CRE AAV–injected mice compared with controls. Moreover, loss of *Sec16b* in the liver does not promote MASH progression in mice fed a MASH-inducing diet. Although the role of hepatic LD expansion in MASH progression remains unclear, one of the most distinctive features of MASLD is the formation of supersized LDs, which can exacerbate disease progression (13). Given that *Sec16b* deficiency impairs LD expansion and leads to microvesicular steatosis, it will be interesting to investigate whether this could contribute to the lack of MASLD progression in LKO mice.

In conclusion, these studies uncover a regulatory mechanism that coordinately controls both VLDL secretion and LD metabolism to maintain lipid homeostasis. Importantly, the GWAS suggests an association between *SEC16B* SNPs and plasma cholesterol levels in humans. Our findings provide proof of concept that targeting SEC16B may represent a potentially safe and effective therapeutic strategy for ASCVD, at least in patients without *LDLR* mutation.

## Methods

### Sex as a biological variable.

Most experiments were conducted in both male and female mice, and no sex differences were observed. For experiments performed in only 1 sex, the sex of the animals is indicated in the figure legends.

### Animal studies.

*Sec16b*^fl/fl^ mice have been described previously (69). *Sec16b*-LKO mice were generated by crossing *Sec16b*^fl/fl^ mice with *Albumin*-Cre transgenic mice (The Jackson Laboratory, 003574). *Sec16b*^–/–^ and *Sec16b*^fl/fl^ mice were crossed with *Ldlr*^–/–^ (The Jackson Laboratory, 002207) to produce *Sec16b*^–/–^ *Ldlr*^–/–^ and *Sec16b*^fl/fl^ *Ldlr*^–/–^ mice. *Hnf4α*-floxed mice were obtained from The Jackson Laboratory (strain 004665). *HNF4*α overexpression was achieved by intravenous injection of AAV8-ALB-h*HNF4α*, as previously described (70).

All mice were housed under pathogen-free conditions in a temperature-controlled room with a 12-hour-light/12-hour-dark cycle and had free access to water and a normal chow diet. In most experiments, both male and female mice (8 to 20 weeks old) were used. Experiments conducted in only 1 sex are indicated in the figure legends. For studies with special diets, 8-week-old mice were placed on an HFD (60% calories from fat, Research Diets, D12492), HCD (70% carbohydrate diet, Teklad diets, TD.98090), or WD (RD Western Diet, Research Diets D12079B). For MASH studies, 8-week-old control and *Sec16b*-LKO mice were placed on MASH diet (21.1% fat, 41% sucrose, and 1.25% cholesterol, Teklad diets, TD.120528) supplemented with 23.1 g/L d-fructose and 18.9 g/L d-glucose in the drinking water for indicated weeks or combined with weekly CCI4 i.p. injection as described previously (48). *Hnf4a*-LKO and *HNF4α* overexpression mice were fed an HFCF diet containing 40% fat/0.2% cholesterol (TestDiet, AIN-76A) and 4.2% fructose (in drinking water) for 20 weeks (70). All mice were fasted for 6 hours or overnight prior to euthanasia, as stated in the figure legends.

Additional details on the methods used in this study can be found in the Supplemental Methods.

### Statistics.

Quantifications of immunoblotting and atherosclerosis lesion area were conducted in ImageJ (NIH). No statistical methods were used to predetermine sample size. Values are presented as mean ± SEM, as indicated in the figure legends. Statistical analyses were performed by GraphPad Prism 10. Statistical significance was calculated by 2-tailed Student’s *t* test, Mann-Whitney test, 1-way ANOVA with Tukey’s post hoc, or 2-way ANOVA with Šidák’s multiple-comparison test, as indicated in the figure legends. Results were considered significant when *P* < 0.05. For mouse experiments, *n* corresponds to the number of mice used. For cell culture experiments, *n* corresponds to the number of independent repeats. For cell culture qPCR and Western blotting, each sample within each biological replicate corresponds to 1 well from a tissue culture plate. For mouse liver Western blotting, each band corresponds to the protein extract from 1 mouse.

### Study approval.

Animal housing and all the experimental procedures were approved by the IACUC at the University of Illinois Urbana-Champaign (protocols 24157 and 24019).

### Data availability.

All data are available in the main text, supplemental materials, and Supporting Data Values file. Proteomics data have been deposited to the ProteomeXchange Consortium via the PRIDE partner repository as PRIDE: PXD056229. This paper uses existing, publicly available data from ChIP-Atlas (SRX2636009, SRX10475653, SRX9853116, SRX4497837, SRX681494, and SRX7860740), ENCODE (ENCFF552YJA and ENCFF798FMB), and the Gene Expression Omnibus (GSE3126). Liver *SEC16B* eQTL data are from the GTEx Portal. Data pertaining to *SEC16B* expression in lean and obese hepatocytes are from Liver Cell Atlas. The lipidomics data are available from the corresponding author on request.

## Author contributions

WL and ZZ designed and performed experiments, analyzed data, and wrote the paper. DM, HF, RS, YT, TY, XZ, and RG performed experiments and analyzed data. YZ, KZ, and JA designed experiments and analyzed data. BW conceived the project, designed and performed experiments, analyzed data, supervised the project, and wrote the paper.

## Conflict of interest

The authors have declared that no conflict of interest exists.

## Funding support

This work is the result of NIH funding, in whole or in part, and is subject to the NIH Public Access Policy. Through acceptance of this federal funding, the NIH has been given a right to make the work publicly available in PubMed Central.

- NIH grants K01DK114373, R01DK128167, and R01HL180740 (to BW).
- NIH grant R01HL147252 (to JA).
- NIH grants R01GM132438 and R01MH124827 (to KZ).
- National Science Foundation (NSF) grant 2121003 (to KZ).
- NSF Science and Technology Center for Quantitative Cell Biology grant 2243257 (to KZ).
- Start-up funds from University of Illinois Urbana-Champaign (to BW).
- Cancer Center at Illinois seed grant (to BW).
- Pfizer Global Medical grant 70228131 (to BW).
- Burnsides Laboratory Research Fund (to BW).
- US Department of Agriculture grant W5002 (to JA).

## Supplementary Material

Supplemental data

Unedited blot and gel images

Supplemental video 1

Supplemental video 2

Supplemental video 3

Supplemental video 4

Supplemental video 5

Supplemental video 6

Supporting data values

## Version 1 04/24/2026

In-Press Preview

## Version 2 07/15/2026

Electronic publication
