# Supplementary material for: Hepatic SEC16B regulates lipid homeostasis by coordinating VLDL secretion and lipid droplet expansion
Source: J Clin Invest. 2026 Apr 24;136(14):e204602. doi: 10.1172/JCI204602 (PMC13367965; doi:10.1172/JCI204602)
Supplement: Supplemental data [file jci-136-204602-s021.pdf]

Hepatic SEC16B regulates lipid homeostasis by coordinating VLDL secretion and lipid droplet expansion

Wei Lu[1], Zhiming Zhao[1], Donald Molina[2], Huaxun Fan[3], Ruicheng Shi[1], Ye Tian[1], Raja Gopoju[4], Tiantian Yang[1], Xinyuan Zhang[1], Yanqiao Zhang[4], Kai Zhang[3, 5], Jaume Amengual[2,6], Bo Wang[1,5,6,7*]

Supplemental Materials

1. Supplemental Methods
2. Supplemental Figures and Figure Legends
3. Supplemental Video Legends

SUPPLEMENTAL METHODS

GWAS and eQTL analysis

Genome-wide association data analysis of the association between *SEC16B* and plasma lipids was performed using LocusZoom (http://locuszoom.org/), based on previously published GWAS data (1). Expression quantitative trait locus (eQTL) data examining SNP rs6682862 and S*EC16B* expression in the liver were obtained from the GTEx database.

Cell culture

Huh7 (a gift from Dr. Kalpana Ghoshal), HEK-293 (purchased from ATCC) and Lenti-X 293T (purchased from Takara Bio) cells were cultured in Dulbecco's modified Eagle's medium (DMEM) supplemented with 10% fetal bovine serum (FBS) and 1% penicillin/streptomycin at 37°C under an atmosphere of 5% CO_2. For plasmids transfection, TransIT-2020 and Lipofectamine 3000 were used according to the manufacturer's instructions.

Primary hepatocytes were isolated as previously described (2). Briefly, the inferior vena cava of anesthetized mice was cannulated, and the liver was perfused at with perfusion medium (0.5 mM EGTA and 10 mM HEPES in Hanks' solution) for 5 minutes, followed by perfusion with 40 μg/mL Liberase TM in 10 mM HEPES-buffered William's Medium E for 10 minutes. The perfused liver was excised, placed in William's Medium E, and gently dispersed. The cell suspension was filtered through a 100-μm strainer and washed three times with William's Medium E. Primary hepatocytes were seeded in collagen–coated plates and collagen-coated glass coverslips in 12-well plates with the plating medium (2 mM glutamine, 10 mM HEPES, 1% penicillin-streptomycin (P/S), and 5% FBS in William's Medium E). After 4 hours, cells were washed twice with PBS and cultured in the plating medium with OA for lipid droplet formation or maintenance medium (2 mM glutamine, 10 mM HEPES, 1% P/S, and 0.2% fatty acid-free BSA in William's Medium E) for other purposes.

Gene expression

Briefly, the liver was homogenized with TissueLyser II (Qiagen, Hilden, Germany), and total RNA was extracted with TRIzol (Invitrogen, Waltham, MA) according to the manufacturer's protocol. cDNA was synthesized, and gene expression was quantified by CFX384 Touch Real-

Time PCR Detection System (Bio-Rad, Hercules, CA) with SYBR Green. 36B4 was used as a reference gene.

Serum and hepatic lipid measurement

Blood was collected by retro-orbital bleeding or from tail tips, and the serum was separated by centrifugation at 8000 rpm, for 5 min at 4°C. Serum lipids were measured with Infinity Triglyceride Reagent (Thermo Fisher) and Infinity Cholesterol Reagent (Thermo Fisher), Wako Free Cholesterol E kit and Wako HR series NEFA-HR (2) kit (FUJIFILM, Richmond, VA) according to the manufacturer's instructions.

For hepatic lipid measurement, snap-frozen liver tissues were cut, weighed, and homogenized in water. Lipids were extracted by adding chloroform/methanol (2:1 v/v), mixing thoroughly by vortex, and separating the aqueous phase and organic phase by centrifuging at 3000 rpm for 5 min at 4°C. The lower organic phase was carefully collected and air-dried. The hepatic lipids were reconstituted in ethanol and diluted in PBS for lipid measurements.

Hepatic VLDL secretion assay

Mice were fasted for 4 hours and then injected retro-orbitally at the dose of 500 mg/kg body weight of Tyloxapol. Subsequently, blood samples were collected at indicated time points and the serum was separated by centrifugation for triglyceride measurement.

ApoB secretion assay

ApoB secretion rates were measured as described previously (3). Following an overnight fast, mice were intraperitoneally injected with 200 µCi of ^{35}S protein labeling mix (Revvity, EasyTag EXPRESS ^{35}S Protein Labeling Mix, NEG772002MC). After 30 min, baseline (T0) blood samples were collected from the tail vein, followed by retro-orbital injection of Tyloxapol. Additional blood samples were collected at 1, 2, 3, and 4 h after Tyloxapol injection. ApoB secretion rates were determined by resolving 1 µl of serum on 8% SDS-PAGE gels. Gels were then fixed, dried, and imaged using a Typhoon Trio laser scanner (GE Healthcare). Secretion rate data are presented as the mean at each time point and as the average slope.

FPLC analysis

Pooled serum samples of the same genotype were fractionated using fast protein liquid chromatography (FPLC) with two Superose 6 10/300 GL columns (G.E. Healthcare, Boston, MA) on a Shimadzu HPLC system (Columbia, MD). Lipid levels in each fraction are measured as described above.

Histology

For Hematoxylin and Eosin (H&E) and Sirius Red staining, liver samples were fixed overnight in 10% formalin, followed by paraffin embedding. Processed samples were trimmed and sectioned into 5 µm slices and stained according to the manufacturer's protocol. For Oil Red O staining, liver samples were embedded in Tissue-Tek OCT compound, frozen on dry ice and stored at -80°C. 8 µm sections were stained with Oil Red O solution.

Untargeted lipidomic analysis

Untargeted lipid profiling analysis was performed by the Metabolomics Core Facility of the Roy J. Carver Biotechnology Center, University of Illinois Urbana-Champaign. Extracted lipids were analyzed using the Thermo Q-Exactive mass spectrometer (MS) system (Bremen, Germany). The Dionex UltiMate 3000 series HPLC system (Thermo, Germering, Germany) was used. Lipids separation was performed on a Thermo Accucore C18 column (2.1 × 150 mm, 2.6 µm) with mobile phase A (60% acetonitrile: 40% water with 10 mM ammonium formate and 0.1% formic acid) and mobile phase B (90% isopropanol: 10% acetonitrile with 10 mM ammonium formate and 0.1% formic acid) with a flow rate of 0.4 ml/min. The linear gradient was as follows: 0 min, 70% A; 4 min, 55% A; 12 min, 35% A; 18 min, 15% A; 20–25 min, 0% A; 26–33 min, 70% A. The autosampler was set to 15°C and the column at 45°C. The injection volume was 10 µl. Mass spectra were acquired under both positive (sheath gas flow rate: 50; aux gas flow rate: 13; sweep gas flow rate: 3; spray voltage: 3.5 kV; capillary temperature: 263°C; aux gas heater temperature: 425°C) and negative electrospray ionization (sheath gas flow rate: 50; aux gas flow rate: 13; sweep gas flow rate: 3; spray voltage: −2.5 kV; capillary temperature: 263°C; aux gas heater temperature: 425°C). Full scan mass spectrum resolution was set to 70,000 with a scan range of m/z 230 to 1,600. The AGC target was 1E6 with a maximum injection time of 200 ms. For MS/MS scan, the mass spectrum resolution was set to 17,500.

All the LC-MS raw data files were performed using MS-DIAL ver.4.90 software for data collection, peak detection, alignment, adduct, and identification. The detailed parameter setting was as follows: MS1 tolerance, 0.01 Da; MS2 tolerance, 0.05 Da; minimum peak height, 10,000 amplitude; mass slice width, 0.05 Da; smoothing method, linear weighted moving average; smoothing level, 3 scans; minimum peak width, 5 scans. [M + H] + , [M + NH4] + , [M + Na] + , [M + K] + , [2M + H] + , [2M + NH4] + , [2M + Na] + , [2M + K] + , [M+H+H2O] + and [M-H]-, [M-2H]2-, [M+HCOO]-, [M + Cl]-, [M + FA-H]- , [M-H-H2O]- were included in adduct ion setting for positive and negative mode, respectively. Compounds were annotated by m/z and MS/MS spectra against the LipidBlast mass spectra database. Internal standards were monitored for retention time and intensity and PCA was used for multivariate statistics and visualization, specifically for outlier detection.

From the MS-DIAL results file, all detected features/metabolites were removed if (sample max)/(blank average) <10. Known (positively identified/annotated) feature/metabolite sample peak heights were normalized to both the sample weight and to internal standards representative of each lipid class. For those without a corresponding labeled internal standard for their lipid class, the average internal standard response of all internal standards was used. Known (positively identified/annotated) features/metabolites were manually evaluated when flagged by specific parameters that required further investigation of identifications. Remaining replicate features were filtered based on manual evaluation of spectra, MS-DIAL total score, dot product, and quality control pooled sample relative standard deviation. Following removal based on the previously mentioned sample max/blank average, all features not positively identified (unknown compounds) that generated both m/z and MS2 data were retained and are reported separately. Unknown compound sample peak heights were normalized to both the sample weight and the average internal standard response of all internal standards was used.

LD proteomics

Delipidated protein pellets were resuspended in 6 M GuHCl in 100 mM TEAB. An aliquot was removed from each for a BCA assay (Pierce) to check protein concentrations, and then 10 mM TCEP and 40 mM CAA (TCI) were added to the remaining samples. The samples were heated to 95°C for 10 minutes to promote reduction and alkylation. After cooling, samples were diluted with 100 mM TEAB and digested with LysC (Fujifilm Wako Chemicals) 1:100 w/w (enzyme:

substrate) for 3 hours. The samples were then digested overnight with trypsin (Pierce) 1:50 w/w at 37°C overnight.

The digested peptides were then acidified with 10% TFA, and 8-20 µg from each were desalted with StageTips. After drying, the samples were suspended in 100 mM TEAB, and the peptide amounts were checked again with a colorimetric peptide BCA assay (Pierce). Each sample was labeled with 0.8mg of a TMT10plex reagent (Thermo Fisher Scientific) for 30 minutes at room temperature. After the reactions were quenched with 5% hydroxylamine for 20 minutes, 5 µg of labeled peptides were taken from each sample and combined into one. This combined sample was desalted with StageTips and dried.

The TMT-labeled peptide sample was dissolved in 5% ACN with 0.1% FA to a concentration of 1.5 µg/µL, and 1 µL was injected into an UltiMate 3000 RSLCnano system coupled to a Q Exactive HF-X mass spectrometer (Thermo). The LC was operated at a flow rate of 300 nL/min with mobile phases of 0.1% FA (A) and 0.1% FA in 80% ACN (B). The peptides were separated with a 25 cm Acclaim PepMap 100 C18 column (2 µm particle size, 75 µm ID) maintained at 50°C over the course of the run. The gradient was 5 to 7% B over 2 minutes, 7 to 32% B over 180 minutes, and then 32 to 55% B over 10 minutes, followed by column washing and equilibration. The mass spectrometer was operated in positive polarity with MS1 scans from 350-1500 *m/z* at 120k resolution (50 ms max IT; 3e6AGC) followed by HCD fragmentation (34 NCE) of the top 20 most abundant ions. MS2 scans starting at 110.0 *m/z* were collected at 45k resolution with an isolation window of 0.7 *m/z*, a maximum IT of 100 ms, and an AGC target of 1e5. Unassigned and singly charged ions were excluded from selection for MS2, and the dynamic exclusion window was 25 s.

The raw LC-MS data was processed with MaxQuant v2.4.0.0 to both identify and quantify the proteins. Settings for the MaxQuant search included peptide mass tolerances of 20 ppm and 6 ppm for the first and main searches, respectively. The fragment mass tolerance was set to 0.02 Da, and the reporter mass tolerance was set to 0.003 Da. A tryptic digest with a maximum of 2 missed cleavages and a minimum peptide length of 6 was specified along with a fixed modification for cysteine carbamidomethylation and variable modifications of protein N-terminal acetylation, methionine oxidation, and N-terminal/lysine TMT10plex labeling. Searches were done against the Uniprot *Mus musculus* reference proteome (54,987 entries; downloaded

July 2023). Using a reverse decoy database strategy, the false discovery rate (FDR) at the PSM and protein levels was set to 1%. Reverse database hits and potential contaminants were removed from the final dataset. Proteomics data have been deposited to the ProteomeXchange Consortium via the PRIDE partner repository as PRIDE: PXD056229.

Plasmids and viruses

Full-length cDNAs used in this study were obtained from Addgene (HA-Sec13 pRK donated by David Sabatini, #46332; pEGFP-Sec23A donated by David Stephens, #66609; hMTP-FLAG donated by Mahmood Hussain, #138335) or purchased from Origen (pcDNA3.1-SEC16B-FLAG) and Genescript (pcDNA3.1-ACSL3-HA, GPAT4-HA, DGAT2-HA, CIDEB-HA, SAR1A-HA). pcDNA-SEC13-GFP was a gift from Dr. Benjamin Glick. AAV-TBG-CRE and AAV-TBG-eGFP (serotype 8) were purchased from Vector Biolabs. Short hairpin RNA (shRNA) targeting *SEC16B* (Forward:CCGGCAGGTGTATAAGCTCCTTTATCTCGAGATAAAGGAGCTTATA and Reverse:AATTCAAAAACAGGTGTATAAGCTCCTTTATCTCGAGATAAAGGAG) was cloned into pLKO.1 vector.

Lentivirus packaging and stable cell line construction

Lentivirus was produced using Lenti-X 293T cells. Briefly, the target plasmid, pCMV-Delta 8.2, and pCMV-VSVG were transfected into cells using Lipofectamine 3000 (Thermo Fisher) reagent according to the manufacturer's protocol. After 18 hours of transfection, the medium was replaced. The medium was harvested after 48 hours of transfection. The medium containing lentivirus was used to infect Huh7 cells for 48 hours followed by antibiotic selection. The selection was done when no cell died in the medium with the antibiotic.

Luciferase assay

HEK-293 cells were plated in 24-well plate and transfected with 0.5 μg firefly luciferase plasmids and renilla luciferase plasmids (1:1 ratio). firefly and renilla luciferase activities were measured at 48 h post-transfection using the Dual-Luciferase Reporter Assay System (Promega) according to the manufacturer's protocol.

Electron microscopy

For VLDL negative staining, 400 µl pooled serum from mice injected with Tyloxapol was overlaid with 600 µl of 1.006 g/ml KBr solution and centrifuged at 100,000 rpm for 2 hours at 16°C in a TLA 100.3 rotor. The top layer was collected as the VLDL fraction. For electron microscopy analysis, 5 µl of the VLDL fraction was applied to carbon-coated copper grids and stained with 2.0% uranyl acetate for 15 min. Grids were visualized with a JEOL 100CX transmission electron microscope. Particle size (diameter) was quantified using ImageJ.

For electron microscopy of liver sections, fresh livers were cut into thin slices and immediately fixed in Karnovsky's fixative. Samples were rinsed, incubated in OsO4 with potassium ferrocyanide, and en-bloc stained with uranyl acetate. After dehydration in ethanol series, samples were infiltrated with 1:1 and 1:4 acetonitrile: epoxy mixture and embedded. Processed samples were trimmed and sectioned into ~100 nm slices and stained with uranyl acetate and lead citrate. Imaging was performed at Electron Microscopy Core at the University of Illinois Chicago. LD size (diameter) was quantified with ImageJ (NIH).

Immunoblotting (IB) and Immunoprecipitation (IP)

Liver samples were homogenized by TissueLyser II in RIPA buffer (50 mM Tris-HCl, pH 7.4, 150 mM NaCl, 1% NP-40, 0.5% sodium deoxycholate, 0.1% SDS) supplemented with protease, phosphatase inhibitors and PMSF. Lysates were sonicated and cleared by centrifugation. Protein lysates, serum and VLDL fractions were mixed with 4x laemmli buffer and size-fractionated on 4%-15% TGX Gels. After separation, the proteins were transferred to hybond PVDF membrane, and incubated with primary antibodies overnight at 4°C: anti-APOB (1:500, Abcam, ab20737), anti-APOB (1:1000, Proteintech, 20578-1-AP), anti-Albumin (1:5000, Proteintech, 66051), anti-GAPDH (1:3000, Sigma-Aldrich, MAB374), anti-MTP (1:500, Santa Cruz Biotechnology, sc-135994), anti-SEC24B (1:1000, Cell Signaling Technology, 12042), anti-SEC23A (1:500, Cell Signaling Technology, 8162), anti-SAR1B (1:500, Santa Cruz Biotechnology, sc-517425), anti-SEC13 (1:500, Santa Cruz Biotechnology, sc-514308), anti-SEC13 (1:1000, R&D Systems, MAB9055), anti-α-TUBULIN (1:500, Santa Cruz Biotechnology, sc-8035), anti-CALNEXIN (1:1000, Abcam, ab10286), anti-RCAS1 (1:1000, Cell Signaling Technology, 12290), anti-HMGB1 (1:5000, Proteintech, 10829-1-AP), anti-FLAG (1:1000, Sigma-Aldrich, F1804), anti-TOM20 (1:1000, Cell Signaling Technology, 72610), anti-PLIN2 (1:500, Santa Cruz Biotechnology, sc-377429), anti-HA (1:1000, Cell Signaling Technology, 3724), anti-CIDEB

(1:1000, Antibodies.com, A92576), anti-SEC31A (1:1000, Proteintech,17913-1-AP), anti-DGAT2 (1:1000, antibodies.com, A89902), anti-GPAT4 (1:1000, antibodies.com, A305880) and anti-SEC31A (1:1000, BD Biosciences, 612350). After incubation with secondary antibodies (Anti-mouse HRP-conjugated (1:3000, Thermo Fisher, 31430) and anti-rabbit HRP-conjugated (1:3000, Thermo Fisher, 65-6120)), the protein bands were visualized with enhanced chemiluminescence (ECL).

Cell lysates were extracted in IP buffer (50 mM Tris, pH 7.5, 150 mM NaCl, 1% NP40, 1 mM EDTA, 10% glycerol) supplemented with protease, phosphatase inhibitors and PMSF. Cell lysates were incubated for 30 mins and then centrifuged. The supernatants were collected for IP, with 5% of the total volume as input. For HA and GFP IP, the supernatants were incubated with 20 µL of anti-HA (Thermo Fisher, 88836) or GFP magnetic beads (Proteintech, gtma) at 4°C overnight. For SEC31A IP, the supernatants were incubated with 1 µg SEC31A antibody (BD Biosciences, 612350) at 4°C overnight and then mixed with Protein A/G Magnetic Beads (Thermo Scientific, 88802) at room temperature for 1 hour. The collected beads were washed three times with IP buffer. The interacting complexes bound to the beads were dissolved in 4x laemmli buffer for 10 minutes with mixing. The samples were then subjected to SDS-PAGE for protein separation, followed by IB.

Immunofluorescence

Huh7 and primary hepatocyte were plated in a 12-well plate with collagen-coated glass coverslips. For LD staining, cells were incubated with oleic acid (200 µM) for the indicated time. Cells were rinsed three times with PBS, fixed with 4% paraformaldehyde in PBS for 10–15 min at RT and rinsed three times with PBS. Cells were permeabilized with 0.1% Triton X-100 in PBS for 10 min at RT and then washed three times with PBS. Cells were blocked with 10% normal goat serum in PBS + 0.1% Tween 20 (PBST) for 1 hour at RT and then incubated with primary antibodies diluted in 1% BSA in PBST overnight at 4 °C: anti-SEC24B (1:500, Cell Signaling Technology, 12042), anti-SEC13 (1:500, R&D Systems, MAB9055), anti-SEC31A (1:500, BD Biosciences, 612350), anti-SAR1-GTP (1:500, NewEast Biosciences, 26916, which specifically recognizes the GTP-bound conformation of SAR1), anti-HA (1:500, Cell Signaling Technology, 3724), anti-FLAG (1:500, Novus Biologicals, NBP1-06712SS), anti-FLAG (1:500, Sigma-Aldrich, F1804), Alexa Fluor® 488 anti-KDEL (1:500, Abcam, ab184819) and anti-PLIN2

(1:300, Santa Cruz Biotechnology, sc-377429). Cells were washed three times with PBS and then incubated with secondary antibodies (anti-Mouse Alexa Fluor™ Plus 488 (1:500, Thermo Fisher, A32723), anti-Mouse Alexa Fluor™ 594 (1:500, Thermo Fisher, A-11005), anti-Mouse Alexa Fluor™ Plus 647 (1:500, Thermo Fisher, A32787TR), anti-Rabbit Alexa Fluor™ 488 (1:500, Thermo Fisher, A11008), anti-Rabbit Alexa Fluor™ Plus 594 (1:500, Thermo Fisher, A32740) and anti-Rat Alexa Fluor™ 647 (1:500, Thermo Fisher, A-21247)) diluted in 1% BSA in PBST for 1 hour at RT and then washed three times with PBS. LDs were stained BODIPY 510 (1:1000, Thermo Fisher, D3823), LipidTOX Red (1:1000, Thermo Fisher, H34476) and MDH (1:1000, Abcepta, SM1000a) for 30 mins. Coverslips were mounted onto microscope slides. The images were taken by confocal microscope. Quantification of colocalization (Pearson's coefficient) was performed by Just Another Colocalization Plugin (JACop) in Image J as described previously (4). Line scan (plot profile) colocalization analysis was performed using ImageJ. Briefly, a straight line was drawn across the region of interest, and fluorescence intensities for each channel were extracted along this line using the plot profile tool in ImageJ. The resulting intensity profiles were plotted as a function of distance from the line origin.

Fractionation of mouse livers

The ER and Golgi fractions were isolated from livers as described (5, 6). Liver samples were homogenized in 1 ml cold homogenization buffer (37.5 mM Tris-maleate; 0.5 M sucrose; 1% dextran; and 5 mM MgCl2, pH 6.4) with a homogenizer at 1,000 rpm for 30 seconds. Homogenized samples were then centrifuged at 5,000 g for 15 min at 4 °C. After initial centrifugation, the supernatant was harvested for ER isolation, and the upper one-third of the pellets were collected and resuspended for Golgi isolation. The collected upper pellets were placed over 1.4 ml 1.2 M sucrose and centrifuged at 100,000 g for 30 min at 4 °C. The top layer and homogenate-sucrose interface were collected and mixed as purified Golgi. Golgi apparatus was diluted by PBS and pelleted after centrifugation at 5,500 rpm for 20 min at 4 °C. As for ER isolation, supernatant from the initial centrifugation was diluted with 0.2 ml homogenization medium plus 5 mM MgCl2 and then centrifuged at 8,500 g for 5 min at 4 °C to exclude mitochondria. The supernatant then was layered onto a sucrose gradient consisting of 0.75 ml 2.0 M sucrose, 1 ml 1.5 M sucrose, and 1 ml 1.3 M sucrose (bottom to top) and ultracentrifuged at 90,000 g for 90 min at 4 °C. ER fraction was collected at 1.3-1.5 M sucrose and 1.5-2.0 M

sucrose interface. The collected ER fraction then was diluted with 3 ml PBS and pelleted at 90,000 g for 60 min at 4 °C. TG level was measured in the ER and Golgi fractions. ApoB protein levels were assessed by western blot analysis.

Nucleus and mitochondria fractions were isolated as previously described (7). Briefly, liver samples were homogenized in cold isolation buffer (IB) (10 mM Tris-MOPS, 1 mM EGTA/Tris pH 7.4, and 200 mM sucrose) and then centrifuged at 700 g for 10 min at 4°C. The pellet is considered as nucleus fraction. The supernatant was then centrifuged at 7,000 g for 10 min at 4°C to collect mitochondria fraction.

Post-nuclear supernatant (PNS), total membrane (TM) and LD fractions were isolated as previously described (8). In brief, liver samples were homogenized in cold buffer A (20 mM tricine, 250 mM sucrose, pH 7.8) containing 0.2 mM PMSF and protease inhibitor and then centrifuged at 3000 g for 10 min at 4°C. The supernatant was PNS. PNS was transferred into an ultracentrifuge tube. The Buffer B (20 mM HEPES, 100 mM KCl, 2 mM MgCl2, pH 7.4) was loaded on the top and centrifuged at 13,000 g for 1 hour at 4 °C. The white top layer was collected as LD fraction. The pellet was TM fraction. LD proteins were extracted with acetone for further analysis. LD protein analysis was performed after normalizing experimental groups on the basis of triglyceride content.

Neutral lipid transfer activity assay

Neutral lipid transfer activity in microsomal fractions was measured using a commercial kit (Sigma-Aldrich, MAK110) as previously (9, 10). Briefly, livers and Huh7 cells were homogenized in the buffer K (10 mM Tris-HCl, 1 mM MgCl2, and 1 mM EGTA, pH 7.4) using a Dounce homogenizer. Microsomes were isolated by centrifugation at 12,000 rpm for 10 min at 4°C. Neutral lipid transfer activity was assayed according to the manufacturer's manual.

Analysis of atherosclerotic lesion

Pictures of aortas were taken after perfusion with saline containing 10% sucrose. The aortic arch and comparable portions of the ascending and descending aorta were carefully cleaned and dissected. Aortic arches were fixed in 10% formalin, stained with Oil Red O solution for 60 min, washed sequentially with 60% isopropanol and H_2O, opened longitudinally to expose atherosclerotic lesions, flattened onto black foam board, and imaged. For aortic root analysis, the

heart and attached aorta were carefully dissected and flushed with PBS. Half of the heart from the apex was removed with blades, and the remaining tissue containing aortic root was embedded in OCT compound, frozen on dry ice and stored at –80°C. For cross-sectional analysis, 6-8 serial 6-μm thick cross sections were collected beginning at the level where all three valve leaflets were clearly visible. For total plaque area quantification, sections were stained with Oil Red O solution. For plaque composition analyses, sections were fixed and permeabilized with ice-cold acetone, blocked, and stained with a rat anti-mouse CD68 primary antibody (clone FA-11; Bio-Rad) followed by biotinylated rabbit anti-rat IgG secondary antibody (Vector Laboratories, Burlingame, CA). $CD68^+$ area was visualized using a Vectastain ABC kit (Vector Laboratories). Sections were then counterstained with hematoxylin, dehydrated in an ethanol gradient, xylene, and mounted with Permount medium (Thermo Fisher Scientific). These sections were also used to quantify the relative necrotic core, which were identified as acellular/anuclerar areas with the lesions, as previously described (11). For collagen content, frozen sections were fixed and stained with picrosirius red (Polysciences) and scanned using both bright field and polarized light using Axioscan.Z1 microscope (Carl Zeiss, Jena, Germany) as described before (12). Total lesion area, $CD68^+$, necrotic core, and collagen-positive areas were quantified using ImageJ software (NIH).

Live cell imaging analysis and quantification

Control and *SEC16B* knockdown Huh7 cells were seeded onto collagen-coated glass-bottom 35 mm dish and transfected with pcDNA-SEC13-GFP. Twenty-four hours post transfection, cells were washed with PBS three times and incubated in phenol red-free DMEM (Gibco, Grand Island, New York) supplemented with glucose, L-glutamine, sodium pyruvate, and 200 µM BSA-oleic acid for one hour before imaging. Live-cell imaging was performed using an inverted epifluorescence microscope (Leica DMi8) equipped with a 100× objective lens, an LED light source (SOLA SE II 365), and an environmental control chamber (InVivoScientific). GFP fluorescence was captured using a GFP filter cube (Leica; excitation: 450/90 nm, dichroic: 495 nm, emission: 550/550 nm) at a frame rate of 1 frame per second. Videos were processed in ImageJ, and 90-s segments were analyzed. Particle trajectories were extracted using the TrackMate plugin (13). Tracks with a duration longer than 5 s were retained for analysis.

REFERENCE

1. Graham SE, Clarke SL, Wu KH, Kanoni S, Zajac GJM, Ramdas S, et al. The power of genetic diversity in genome-wide association studies of lipids. *Nature.* 2021;600(7890):675-9.
2. Rong X, Wang B, Palladino EN, de Aguiar Vallim TQ, Ford DA, and Tontonoz P. ER phospholipid composition modulates lipogenesis during feeding and in obesity. *J Clin Invest.* 2017;127(10):3640-51.
3. Zhou F, Wu X, Pinos I, Abraham BM, Barrett TJ, von Lintig J, et al. beta-Carotene conversion to vitamin A delays atherosclerosis progression by decreasing hepatic lipid secretion in mice. *J Lipid Res.* 2020;61(11):1491-503.
4. Bolte S, and Cordelieres FP. A guided tour into subcellular colocalization analysis in light microscopy. *J Microsc.* 2006;224(Pt 3):213-32.
5. Tian Y, Mehta K, Jellinek MJ, Sun H, Lu W, Shi R, et al. Hepatic Phospholipid Remodeling Modulates Insulin Sensitivity and Systemic Metabolism. *Adv Sci (Weinh).* 2023;10(18):e2300416.
6. Croze EM, and Morre DJ. Isolation of plasma membrane, golgi apparatus, and endoplasmic reticulum fractions from single homogenates of mouse liver. *J Cell Physiol.* 1984;119(1):46-57.
7. Frezza C, Cipolat S, and Scorrano L. Organelle isolation: functional mitochondria from mouse liver, muscle and cultured fibroblasts. *Nat Protoc.* 2007;2(2):287-95.
8. Ding Y, Zhang S, Yang L, Na H, Zhang P, Zhang H, et al. Isolating lipid droplets from multiple species. *Nat Protoc.* 2013;8(1):43-51.
9. Athar H, Iqbal J, Jiang XC, and Hussain MM. A simple, rapid, and sensitive fluorescence assay for microsomal triglyceride transfer protein. *J Lipid Res.* 2004;45(4):764-72.
10. Anaganti N, Rajan S, and Hussain MM. An improved assay to measure the phospholipid transfer activity of microsomal triglyceride transport protein. *J Lipid Res.* 2021;62:100136.
11. Pinos I, Blanco A, Kelschenbach J, Veenstra M, Hu E, He H, et al. EcoHIV Infection Promotes Atherosclerosis Progression in LDLR-Deficient Mice. *Arterioscler Thromb Vasc Biol.* 2025;45(10):e470-e82.

12. Pinos I, Coronel J, Albakri A, Blanco A, McQueen P, Molina D, et al. beta-Carotene accelerates the resolution of atherosclerosis in mice. *Elife.* 2024;12.

13. Tinevez JY, Perry N, Schindelin J, Hoopes GM, Reynolds GD, Laplantine E, et al. TrackMate: An open and extensible platform for single-particle tracking. *Methods.* 2017;115:80-90.

SUPPLEMENTAL FIGURES

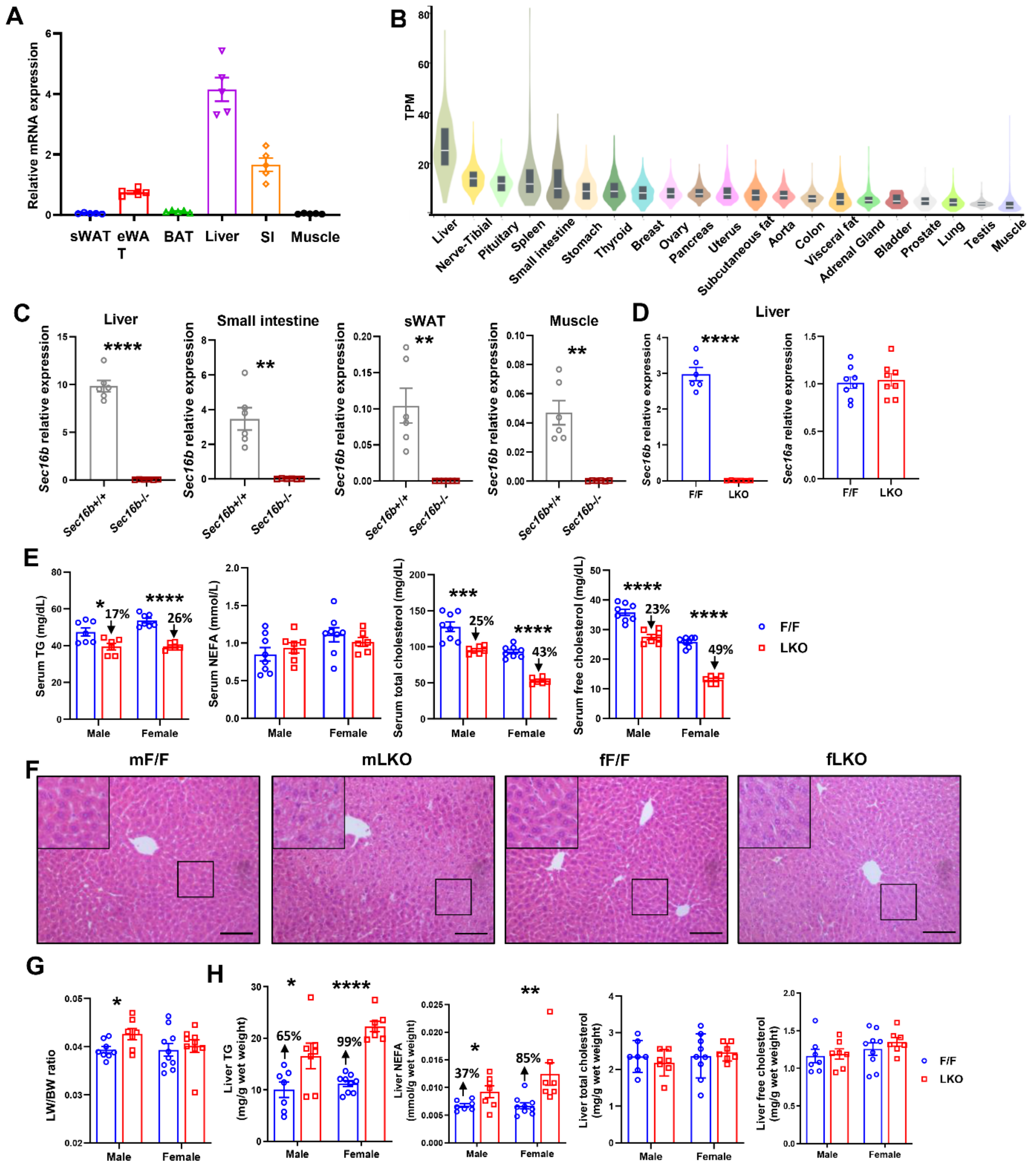

Supplemental Figure 1: Characterization of LKO mice fasted for 6 h.

(A) *Sec16b* mRNA levels in different metabolic tissues of male mice (n=5), subcutaneous white adipose tissues (sWAT), epididymal white adipose tissue (eWAT), brown adipose tissue (BAT) and small intestine (SI).

(B) *SEC16B* mRNA levels in different tissues of humans. Data were derived from GTEx project.
(C) *Sec16b* mRNA levels in selected tissues of male *Sec16b+/+* and *Sec16b-/-* mice fasted for 6 h (n=6).

(D) *Sec16b* and *Sec16a* mRNA levels in the livers of male control (*Sec16b*$^{F/F}$, F/F) and LKO (*Sec16b*$^{F/F}$, *Albumin*-Cre) mice fasted for 6 h (n=6-8).
(E) Serum lipid levels of control (F/F) and LKO mice fasted for 6 h (n=6-8).
(F) Representative histology of livers from control (F/F) and LKO mice fasted for 6 h. Scale bar = 100 μm.
(G-H) Liver/body weight ratio and hepatic lipid levels of control (F/F) and LKO mice fasted for 6 h (n=6-10).
Values are means ± SEM. Statistical analysis was performed with Student's t test. *P < 0.05, **P < 0.01, ***P < 0.001, ****P < 0.0001.

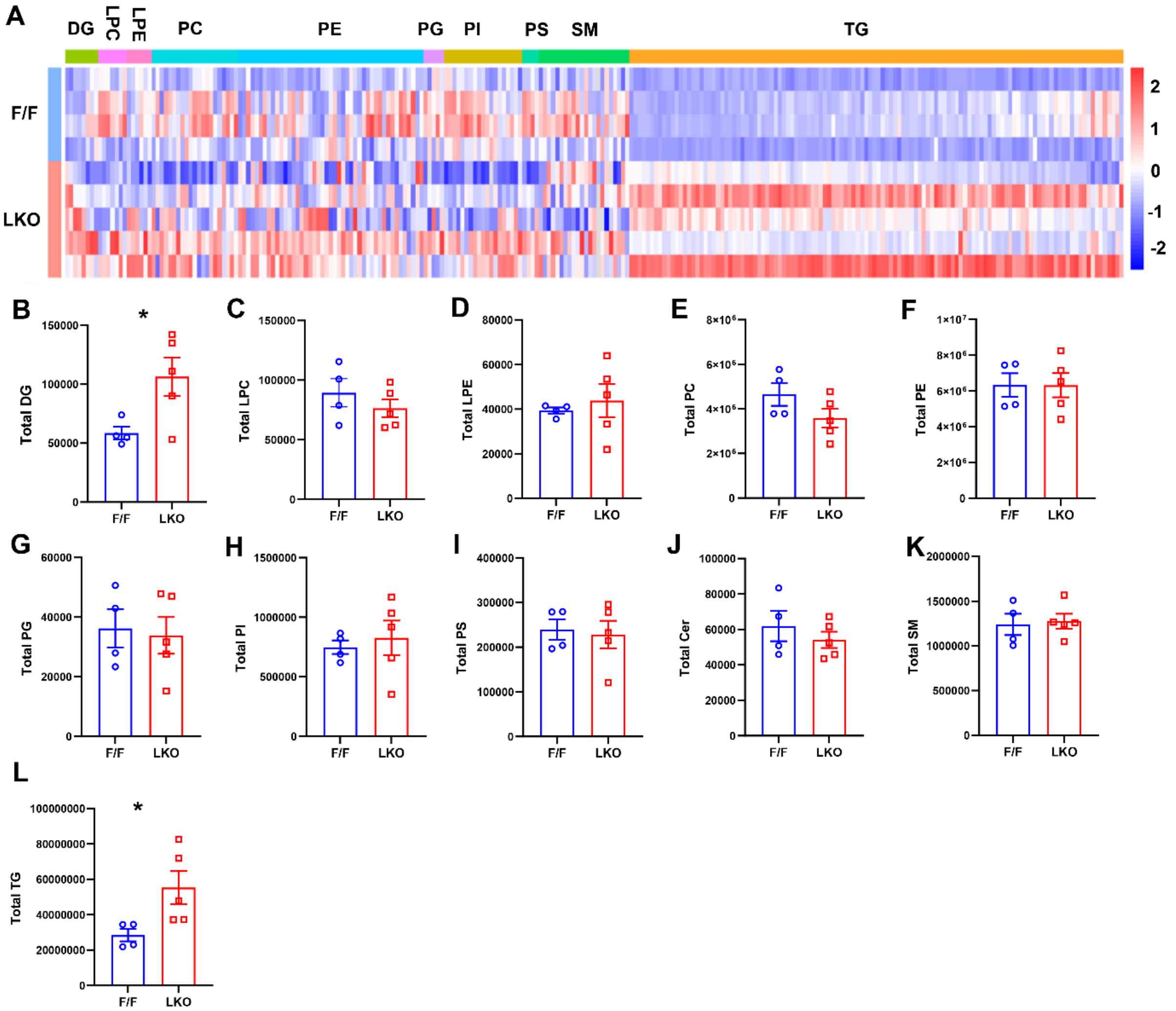

Supplemental Figure 2: Lipidomic analysis of livers from male control (F/F) and LKO mice after 16 h fasting.

(A) Heatmap of different lipid species (n=4-5).

(B-L) Total levels of DG, LPC, LPE, PC, PE, PG, PI, PS, Cer, SM and TG (n=4-5).

Values are means ± SEM. Statistical analysis was performed with Student's t test. *$P < 0.05$.

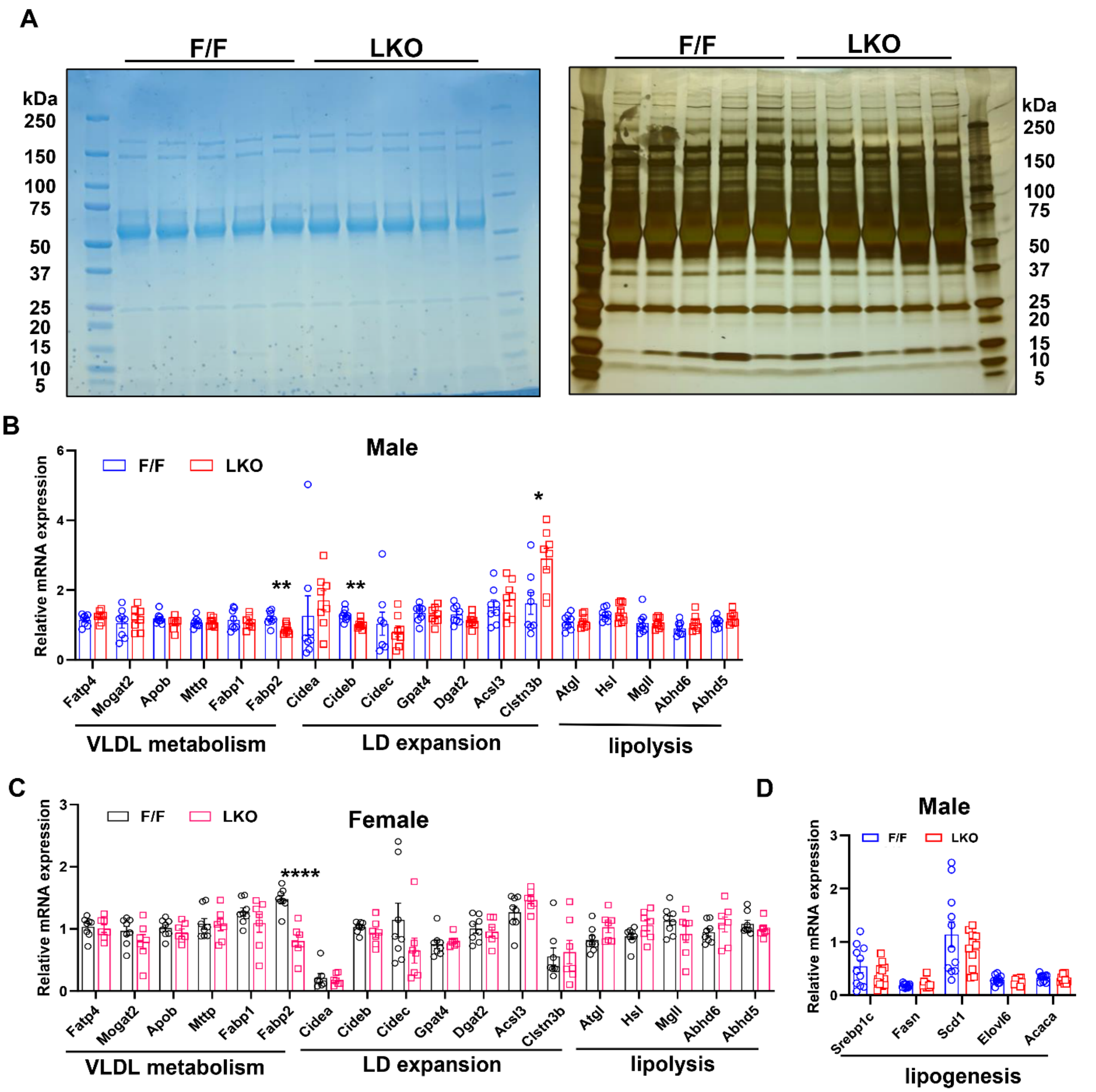

Supplemental Figure 3: The effect of hepatic *Sec16b* deletion on serum proteins and the expression of selected genes in the livers of control (F/F) and LKO mice after 16 h fasting.

(A) Coomassie blue and silver staining of serum proteins from male control (F/F) and LKO mice after 16 h fasting (n=5).

(B-D) mRNA levels of indicated genes in the livers from control (F/F) and LKO mice after 16 h fasting (B: n=8, C: n=7-8, D: n=11).

Values are means ± SEM. Statistical analysis was performed with Student's t test. *P < 0.05, **P < 0.01, ****P < 0.0001.

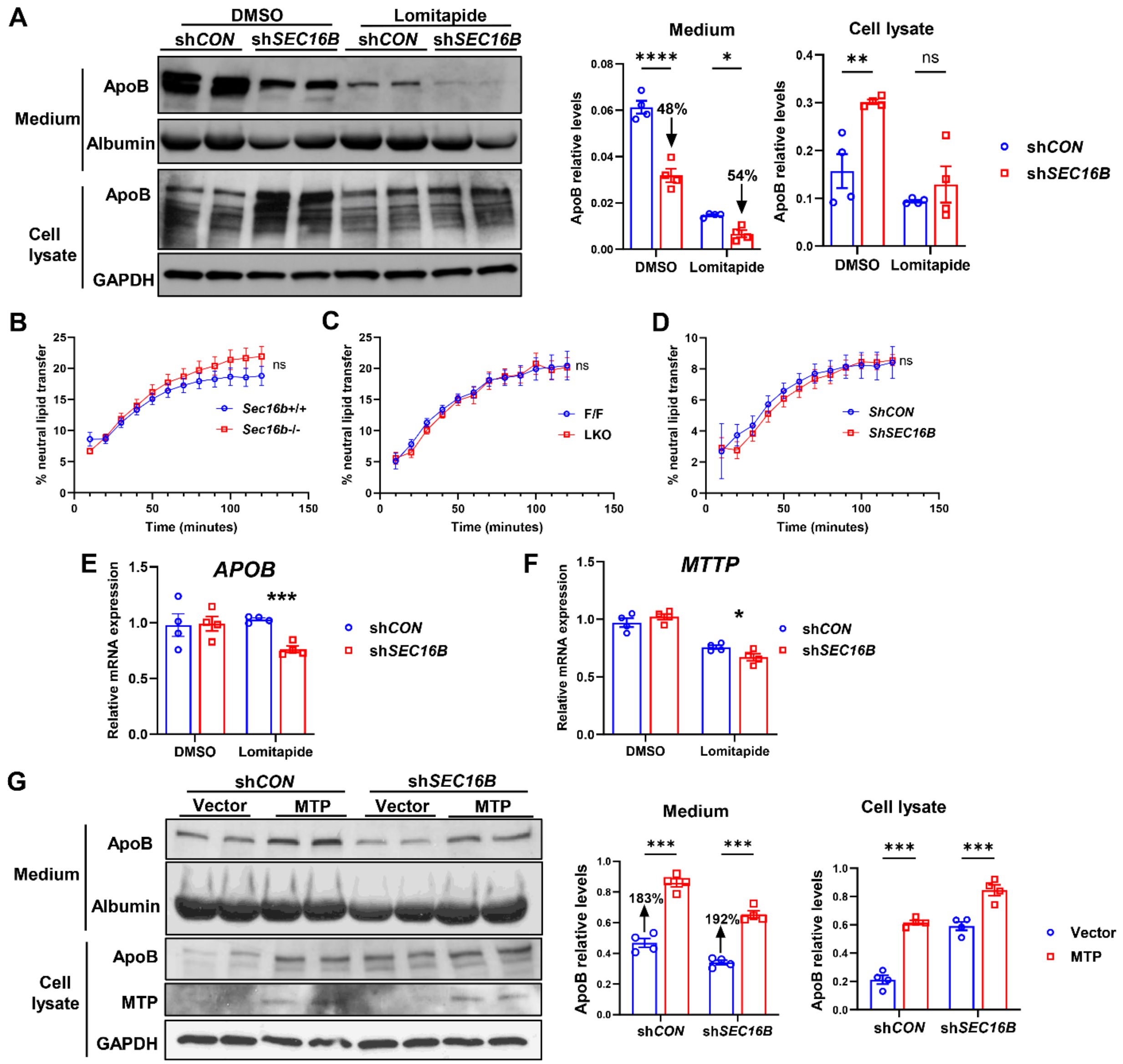

Supplemental Figure 4: SEC16B controls VLDL secretion through mechanism that are, at least partially, independent of MTP activity.

(A) Representative western blot images and quantification of ApoB levels in the medium and cell lysate of sh*CON* and sh*SEC16B* Huh7 cells cultured in DMEM+OA and treated with DMSO or 1 μM Lomitapide for 16 h (n=4). Albumin and GAPDH were used as a loading control.

(B) Neutral lipid transfer activity of MTP in the hepatic microsomal fractions of *Sec16b+/+* and *Sec16b-/-* mice fasted for 6 h (n=5).

(C) Neutral lipid transfer activity of MTP in the hepatic microsomal fraction of control (F/F) and LKO mice fasted for 16 h (n=6).

(D) Neutral lipid transfer activity of MTP in the microsomal fraction of sh*CON* and sh*SEC16B* Huh7 cells cultured in DMEM and OA for 16 h (n=4).
(E-F) *APOB* and *MTTP* mRNA levels in sh*CON* and sh*SEC16B* Huh7 cells cultured in DMEM+OA and treated with DMSO or 1 μM Lomitapide for 16 h (n=4).
(G) Representative western blot images and quantification of ApoB levels in the medium and cell lysate of sh*CON* and sh*SEC16B* Huh7 cells overexpressing MTP-FLAG and cultured in DMEM+OA for 16 h (n=4). Albumin and GAPDH were used as a loading control.
Values are means ± SEM. Statistical analysis was performed with two-way ANOVA (A-D, G) or Student's t test (E-F). ns: not significant. *P < 0.05, **P < 0.01, ***P < 0.001, ***P < 0.001.

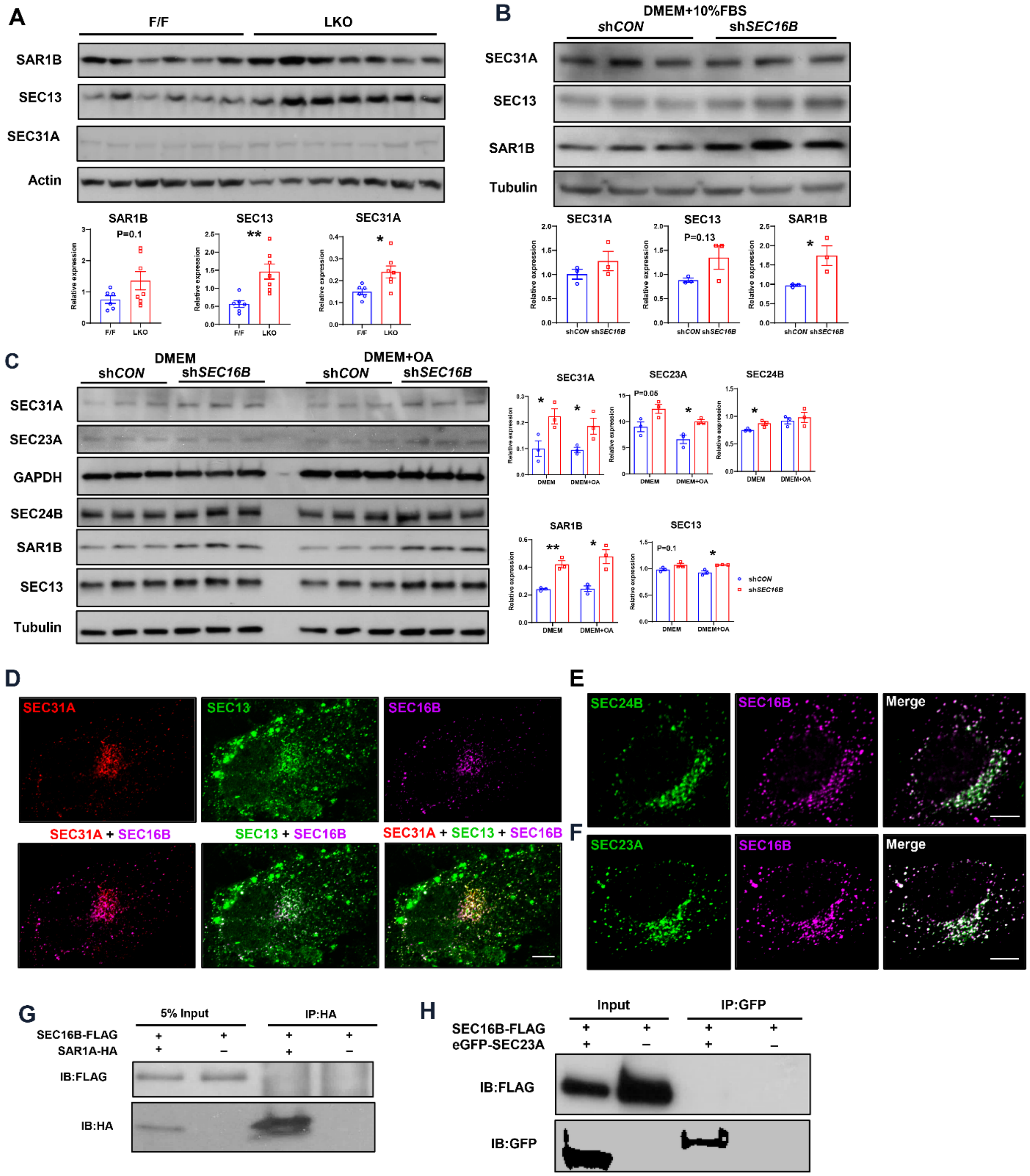

Supplemental Figure 5: *SEC16B* knockdown affects COPII assembly.

(A) Western blot analysis and quantification of SEC13, SAR1B and SEC31A in the livers of control (F/F) and LKO mice after 16 h fasting.

(B) Western blot analysis and quantification of SEC31A, SEC13 and SAR1B in sh*CON* and sh*SEC16B* Huh7 cells cultured with DMEM+10% FBS.
(C) Western blot analysis and quantification of SEC31A, SEC13, SEC23A, SEC24B and SAR1B in sh*CON* and sh*SEC16B* Huh7 cells cultured in DMEM or DMEM+OA for 6 h.
(D) Confocal microscope image of Huh7 cells transfected with SEC16B-FLAG (purple) and co-stained with endogenous SEC13 (green) and SEC31A (red) (n=3). Scale bar = 8 μm.
(E) Confocal microscope image of Huh7 cells transfected with SEC16B-FLAG (purple) and co-stained with endogenous SEC24B (green) (n=3). Scale bar = 8 μm.
(F) Confocal microscope image of Huh7 cells transfected with SEC16B-FLAG (purple) and eGFP-SEC23A (green) (n=3). Scale bar = 8 μm.
(**G**) Co-IP assay in SEC16B-FLAG and SAR1A-HA transfected HEK293T cells (n=3).
(H) Co-IP assay in SEC16B-FLAG and eGFP-SEC23A transfected HEK293T cells (n=3).
Values are means ± SEM. Statistical analysis was performed with Student's t test. *$P < 0.05$, **$P < 0.01$.

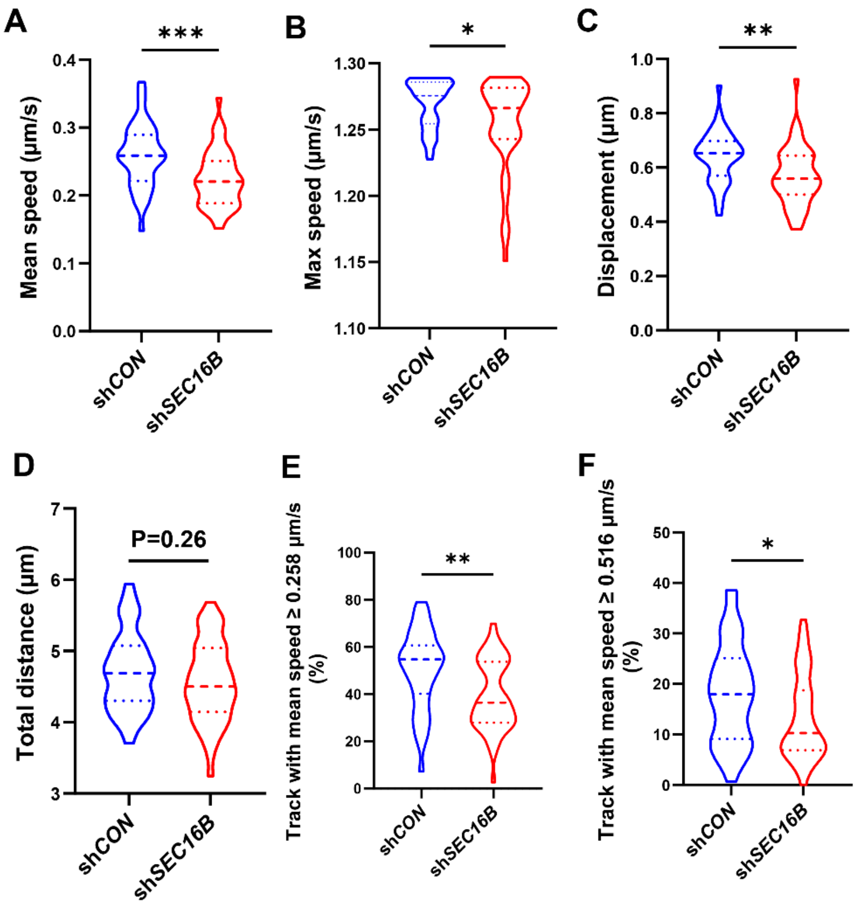

Supplemental Figure 6. Quantification of live cell imaging data.

(A-B) Mean and maximum speed of puncta movement in control (n=39 cells) and *SEC16B* knockdown (n=48 cells) cells.

(C-D) Average displacement and total distance puncta travel in each cell.

(E-F) Percentage of puncta with mean speed ≥ 0.258 μm/s (E) and ≥ 0.516 μm/s (F). One pixel is approximately 0.258 μm.

Values are presented as violin plot. Statistical analysis was performed with Student's t test. *P < 0.05, **P < 0.01, ***P < 0.001.

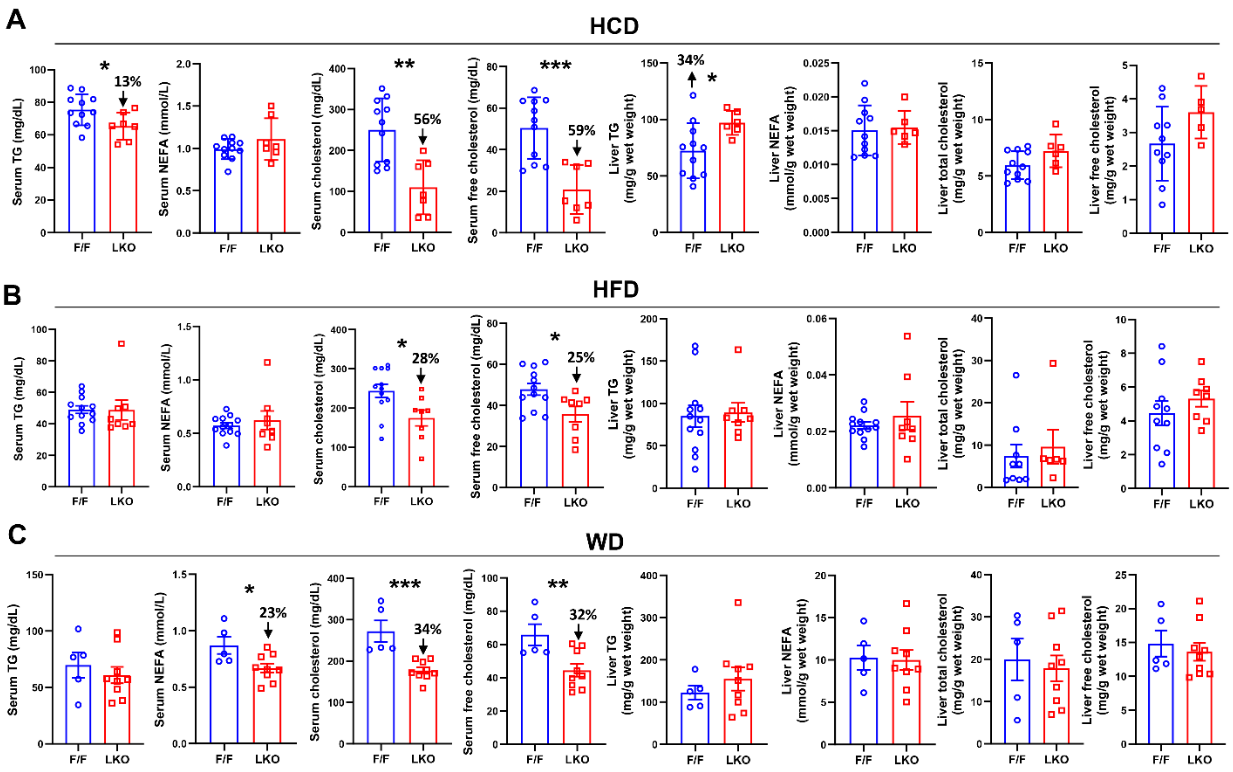

Supplemental Figure 7: Serum and hepatic lipid levels of male control (F/F) and LKO mice fed HCD, HFD and WD.

(A) Serum and hepatic lipid levels of 8 weeks old male control (F/F) and LKO mice fed HCD for 6 weeks (n=5-11).

(B) Serum and hepatic lipid levels of 8 weeks old male control (F/F) and LKO mice fed HFD for 12 weeks (n=6-12).

(C) Serum and hepatic lipid levels of 8 weeks old male control (F/F) and LKO mice fed WD for 12 weeks (n=5-9).

Values are means ± SEM. Statistical analysis was performed with Student's t test. $^*P < 0.05$, $^{**}P < 0.01$, $^{***}P < 0.001$.

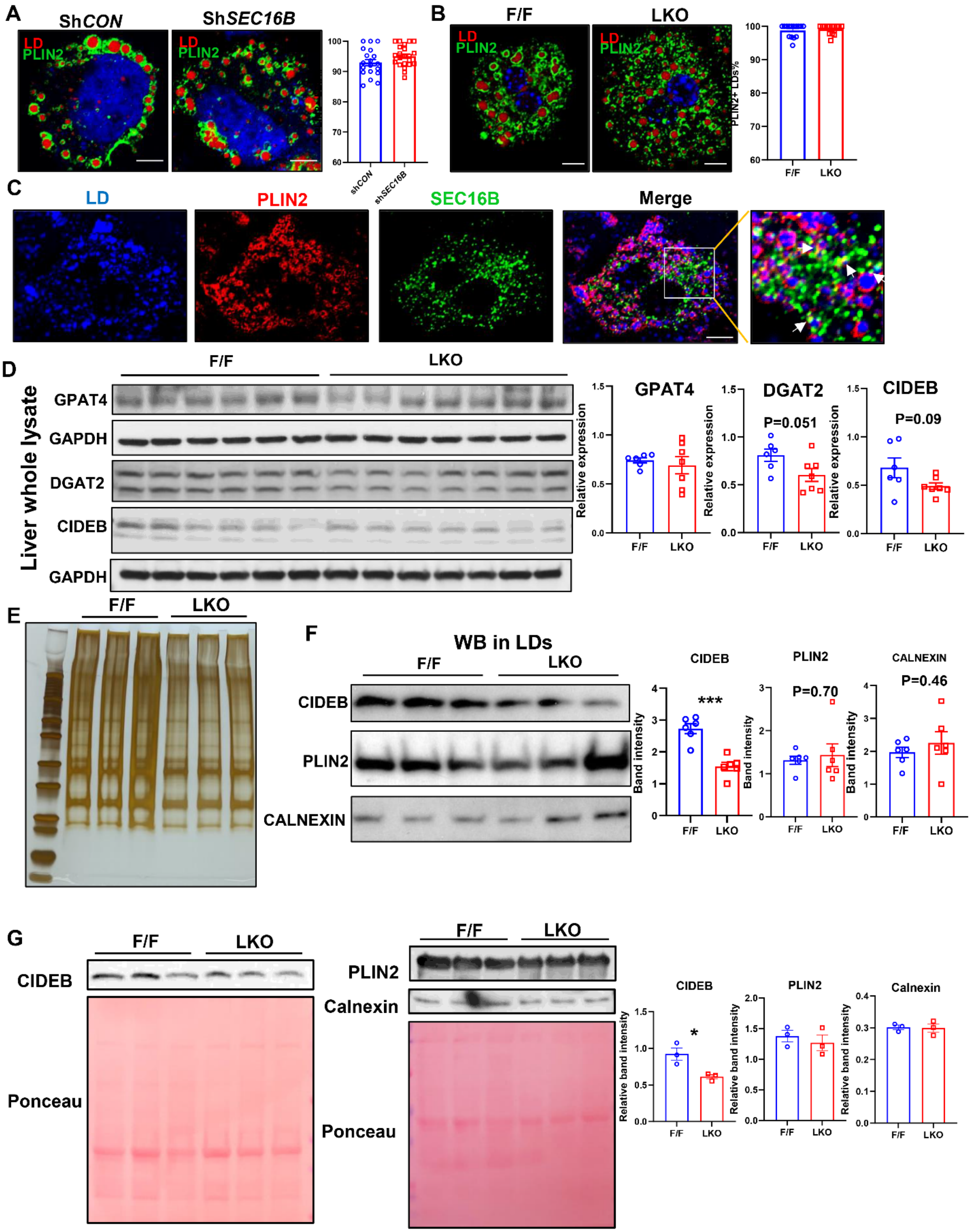

Supplemental Figure 8: SEC16B partially localizes to cytoplasmic LDs and its disruption does not affect the percentage of cytoplasmic LDs.

(A) Confocal microscope image of 6 h OA-treated sh*CON* and sh*SEC16B* Huh7 cells stained PLIN2 (green) and LD (red). Scale bar = 8 μm.
(B) Confocal microscope image of 6 h OA-treated male F/F and LKO primary hepatocytes stained with PLIN2 (green) and LD (red) (n=3). Scale bar = 8 μm.
(C) Confocal microscope image of 6 h OA-treated Huh7 cells transfected with SEC16B-FLAG (green) and co-stained with LDs (Blue) and PLIN2 (red) (n=3). Scale bar = 8 μm.
(D) Western blot analysis and quantification of GPAT4, DGAT2 and CIDEB in whole lysates from livers of male control (F/F) and LKO mice after 16 h fasting (n=6-7).
(E) Silver staining of LDs isolated from control (F/F) and LKO female mouse livers after 16 h fasting. Equal amount of TG from each sample was loaded onto the gels (n=3).
(F) Representative Western blot images and quantification of CIDEB, PLIN2 and CALNEXIN in LD fractions from livers of female control (F/F) and LKO mice after 16 h fasting (n=6). Equal amount of TG from each sample was loaded onto the gels.
(G) Western blot analysis and quantification of CIDEB, PLIN2 and CALNEXIN in LD fractions from livers of female control (F/F) and LKO mice after 16 h fasting (n=6). Equal amount of total proteins from each sample was loaded onto the gels.
Values are means ± SEM. Statistical analysis was performed with Student's t test (A, D, F) or Mann-Whitney test (B).

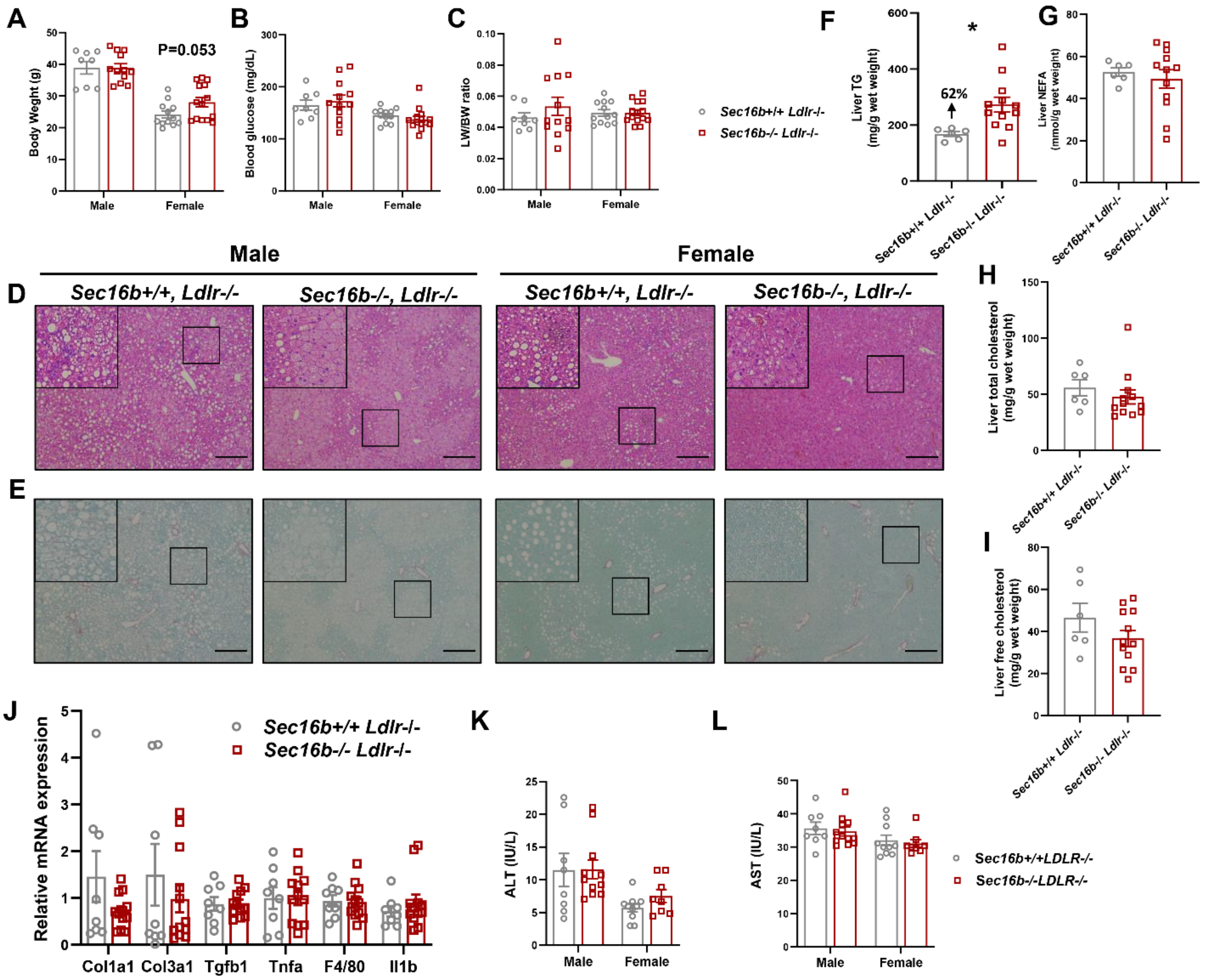

Supplemental Figure 9: The effect of whole body *Sec16b* knockout on the livers of *Ldlr* null mice.

(A-C) Body weight, blood glucose and liver/body weight ratio of 8 weeks old *Sec16b+/+ Ldlr-/-* and *Sec16b-/- Ldlr-/-* mice fed WD for 12 weeks (n=8-14).

(D-E) H&E (D) and Sirius red staining(E) of livers from 8 weeks old *Sec16b+/+ Ldlr-/-* and *Sec16b-/- Ldlr-/-* mice fed WD for 12 weeks (n=3). Scale bar = 200 μm.

(F-I) Hepatic lipid levels of 8 weeks old male *Sec16b+/+ Ldlr-/-* and *Sec16b-/- Ldlr-/-* mice fed WD for 12 weeks (n=6-12).

(J) The expression of genes involved in fibrogenesis and inflammation in the livers of male *Sec16b+/+ Ldlr-/-* and *Sec16b-/- Ldlr-/-* mice fed WD for 12 weeks (n=8-12).

(K-L) Serum ALT and AST levels in 8 weeks old *Sec16b+/+ Ldlr-/-* and *Sec16b-/- Ldlr-/-* mice fed WD for 12 weeks (n=8-12).

Values are means ± SEM. Statistical analysis was performed with Student's t test. $*P < 0.05$.

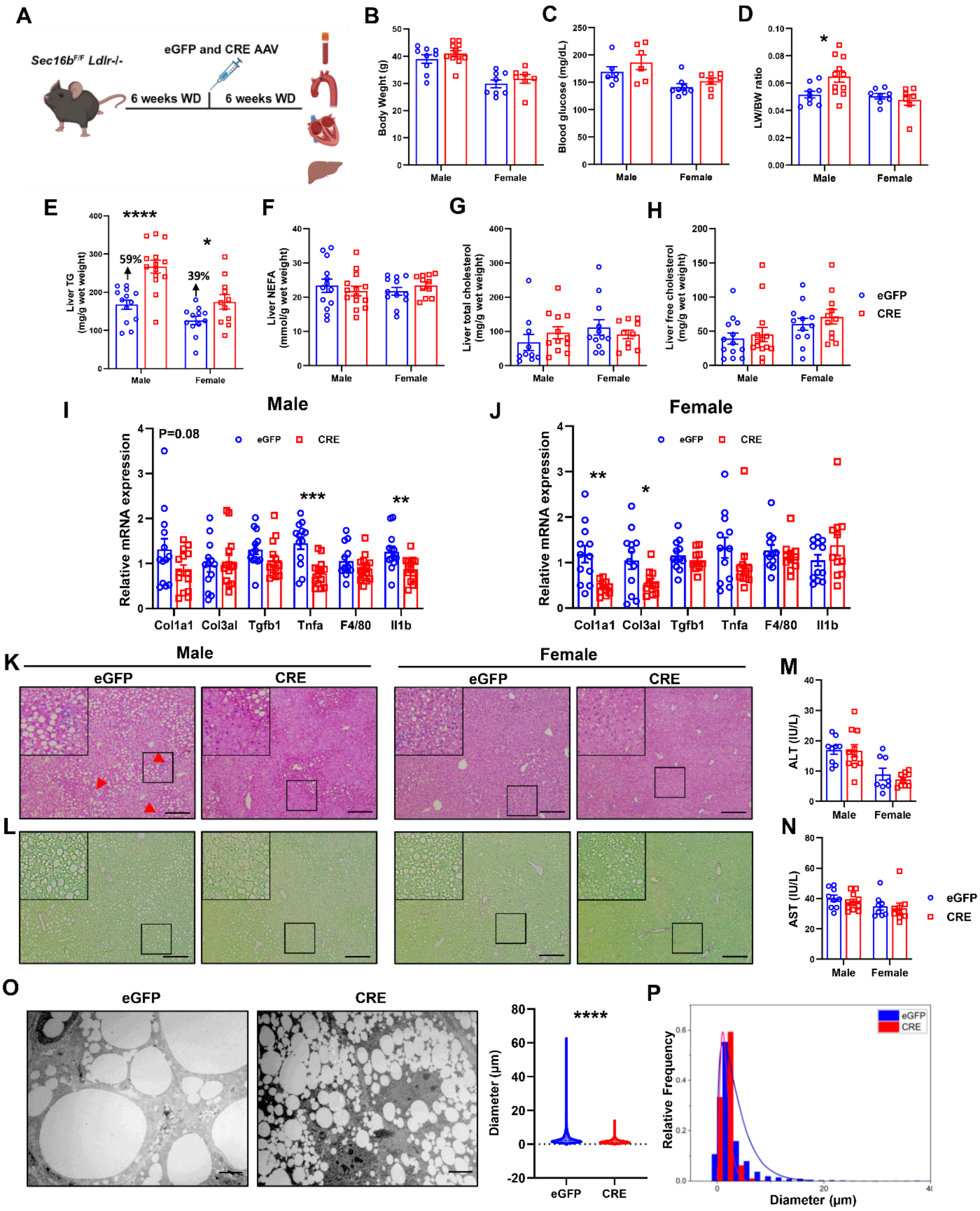

Supplemental Figure 10: Characterization of WD-fed *Sec16b*$^{F/F}$(F/F) *Ldlr*-/- mice injected with eGFP or CRE AAV.

(A) Diagram of experimental strategy of acute hepatic *Sec16b* deletion after WD feeding in atherosclerosis model.

(B-D) Body weight, Blood glucose and liver/body weight ratio of 12-week WD-fed *Sec16b*$^{F/F}$(F/F) *Ldlr*-/- mice receiving eGFP or CRE AAV (B: n=7-11, C: n=6-8, D: n=7-11).

(E-H) Hepatic lipid levels of 12-week WD-fed *Sec16b*$^{F/F}$(F/F) *Ldlr*-/- mice receiving eGFP or CRE AAV (n=11-14).

(I-J) The expression of genes involved in fibrogenesis and inflammation in the livers of 12-week WD-fed *Sec16b*$^{F/F}$(F/F) *Ldlr*-/- mice receiving eGFP or CRE AAV (I: n=13-14, J: n=11-12).

(K-L) H&E (K) and Sirius red staining (L) of livers from 12-week WD-fed *Sec16b*$^{F/F}$(F/F) *Ldlr*-/- mice receiving eGFP or CRE AAV (n=3). Red arrows indicate immune cell infiltration. Scale bar = 200 μm.

(M-N) Serum ALT and AST levels in 12-week WD-fed *Sec16b*$^{F/F}$(F/F) *Ldlr*-/-mice receiving eGFP or CRE AAV (n=8-11).

(O-P) Representative images and quantification of LD size in the livers of 12-week WD-fed male *Sec16b*$^{F/F}$(F/F) *Ldlr*-/- mice receiving eGFP or CRE AAV (n=3). Scale bar = 8 μm.

Values are means ± SEM or violin plot. Statistical analysis was performed with Student's t test (B-J, M-N) or Mann-Whitney test (O). *P < 0.05, **P < 0.01, ***P < 0.001, ****P < 0.0001.

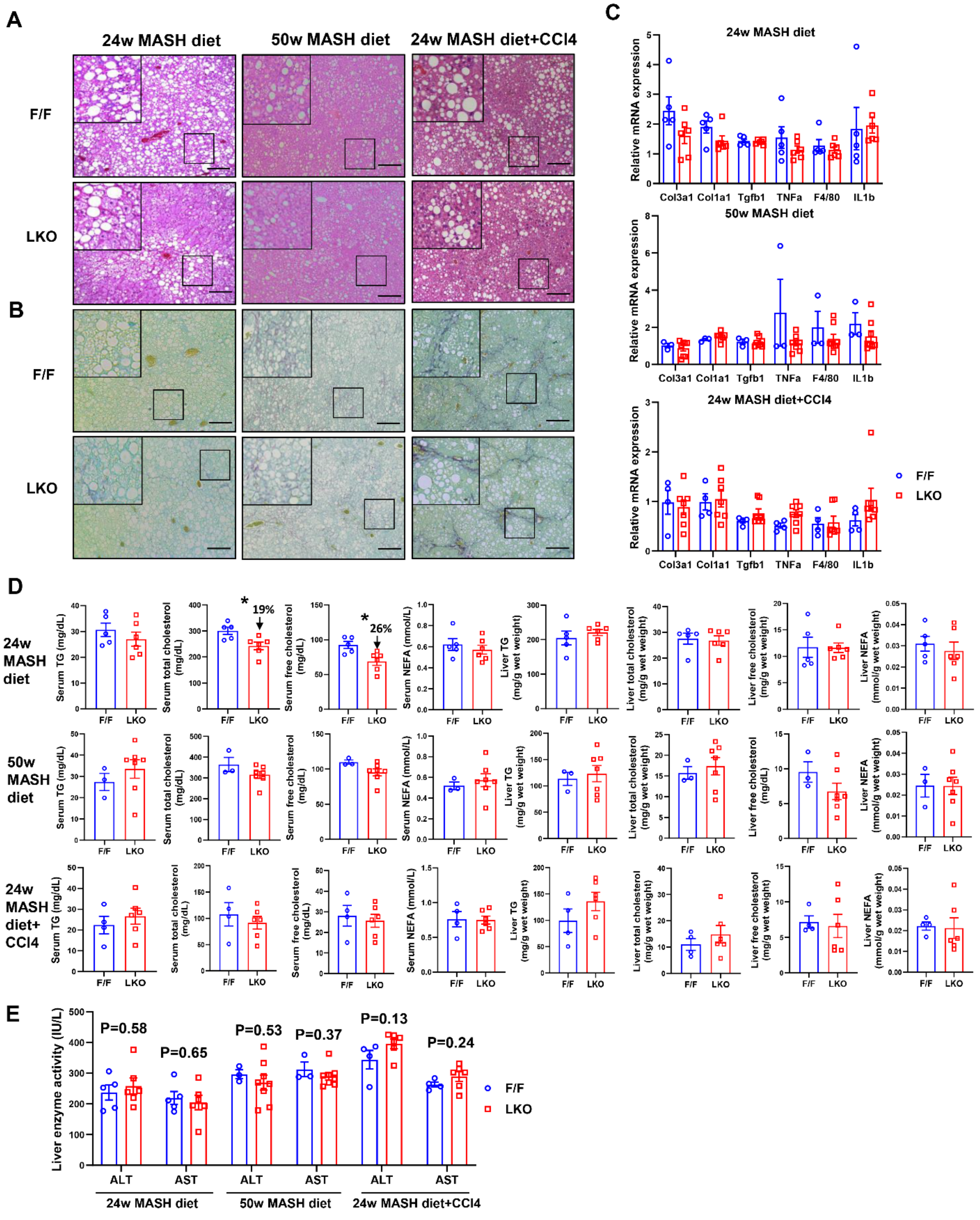

Supplemental Figure 11: *Sec16b* deletion in the liver does not affect MASLD progression. **(A-B)** Representative H&E staining (A) and Sirius red staining (B) of livers from 8 weeks old male control (F/F) and LKO mice fed MASH diet and a high sugar solution for 24 weeks, 50

weeks or MASH diet and a high sugar solution plus CCl_4 injection for 24 weeks (n=3-6). Scale bar = 200 μm.

(C) The expression of genes involved in fibrogenesis and inflammation in the livers of male control (F/F) and LKO mice as in A (n=3-8).

(D) Serum and hepatic lipid levels in male control (F/F) and LKO mice fed MASH diets as in A (n=3-7).

(E) Serum ALT and AST levels in male control (F/F) and LKO mice fed MASH diets as in A (n=3-8).

Values are means ± SEM. Statistical analysis was performed with Student's t test. $*P < 0.05$.

SUPPLEMENTAL VIDEOS

Supplemental Video 1-6. Representative live-cell imaging videos of SEC13-GFP in shCON (1-3) and sh*SEC16B* (4-6) Huh7 cells. Cells were transiently transfected with pcDNA-SEC13-GFP and treated with 200 μM BSA–oleic acid for 1 hour prior to imaging.
