# Supplementary material for: Hepatic SEC16B regulates lipid homeostasis by coordinating VLDL secretion and lipid droplet expansion
Source: J Clin Invest. 2026 Apr 24;136(14):e204602. doi: 10.1172/JCI204602 (PMC13367965; doi:10.1172/JCI204602)

Fig. 3D

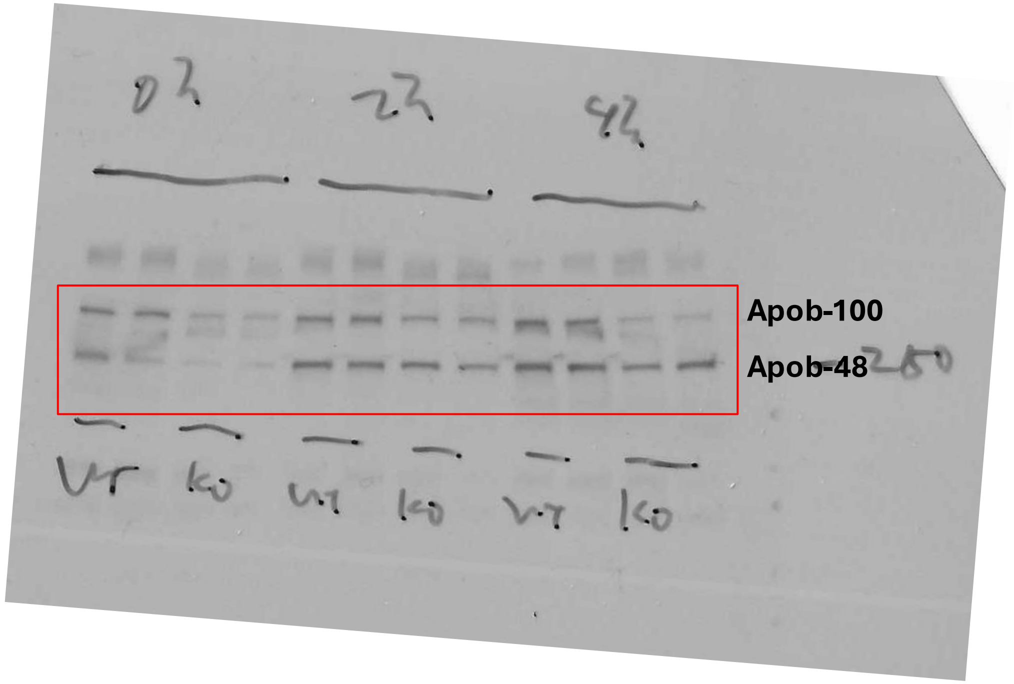

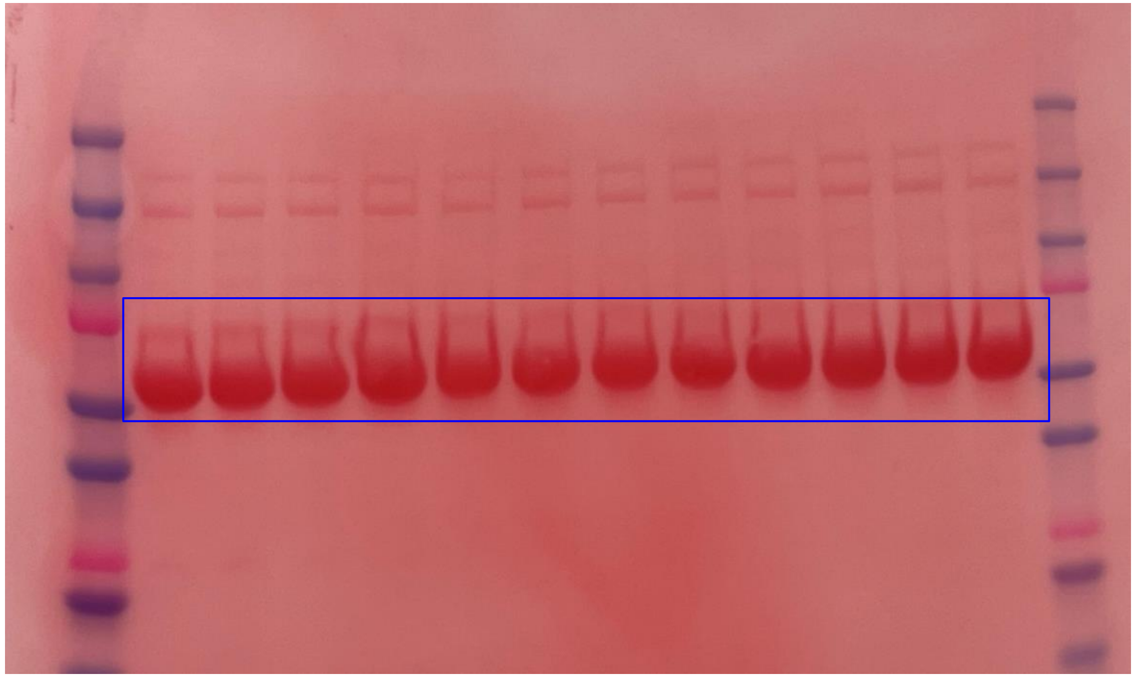

Fig. 3E

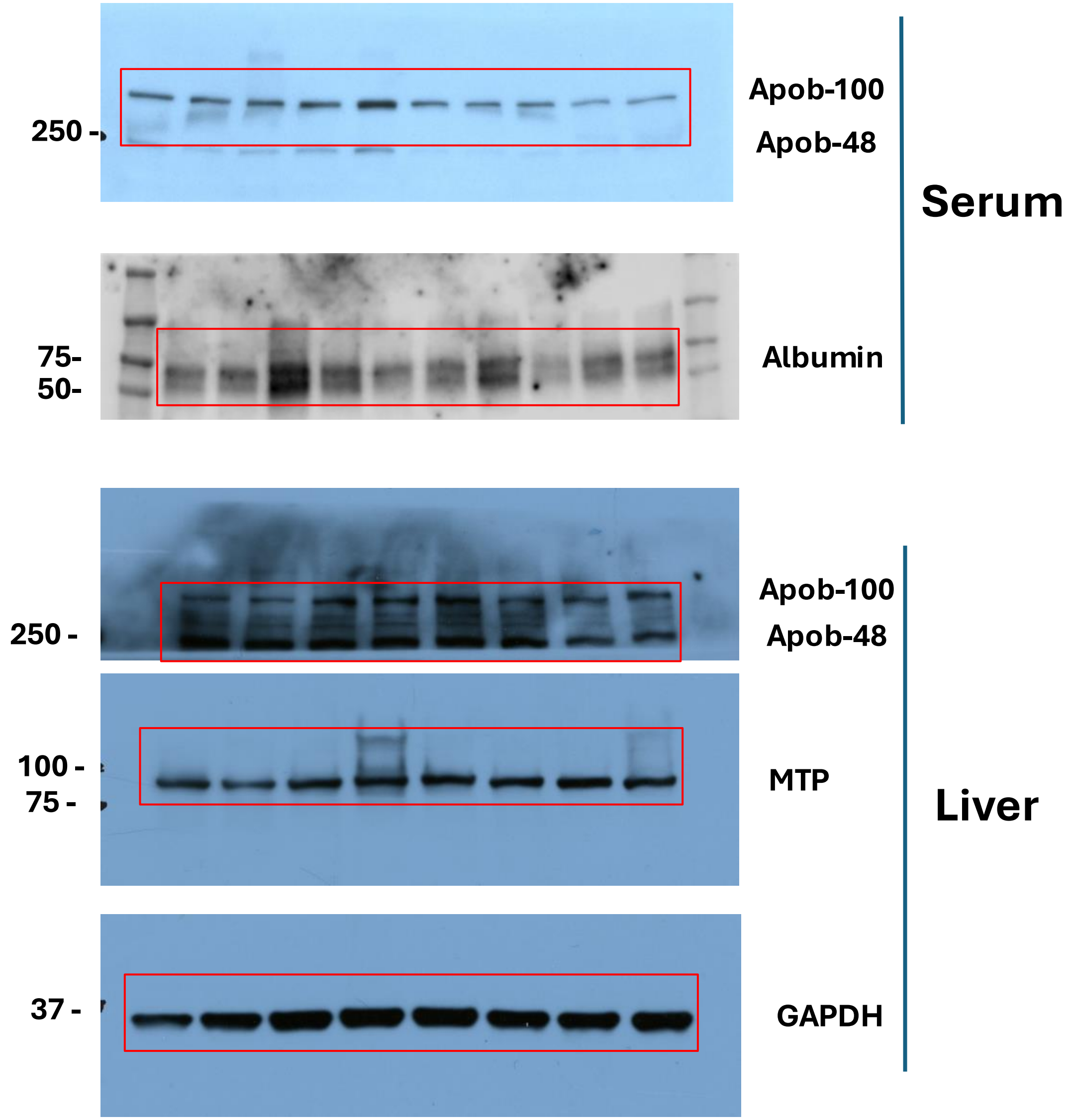
250 -
Apob-100
Apob-48
75-
50-
Albumin
Serum
250 -
Apob-100
Apob-48
100 -
75 -
MTP
37 -
GAPDH
Liver

Fig. 3G

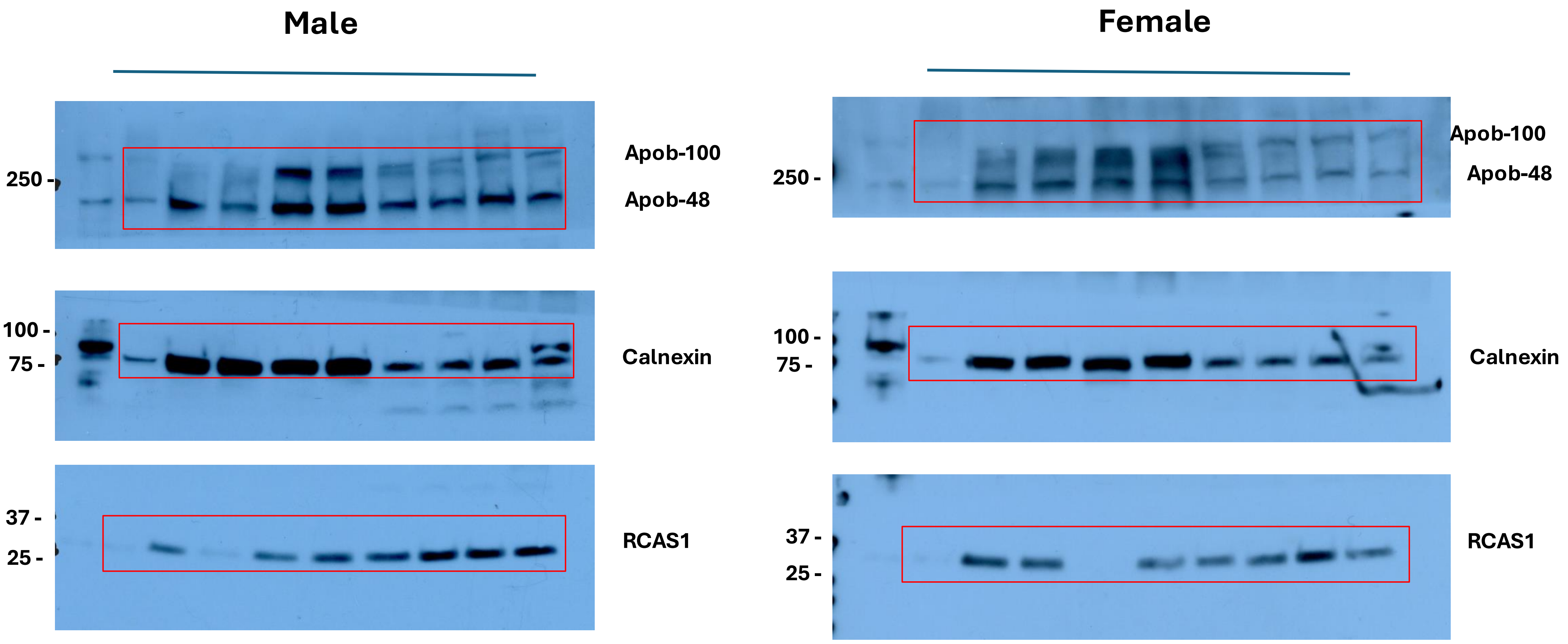

Fig. 4C

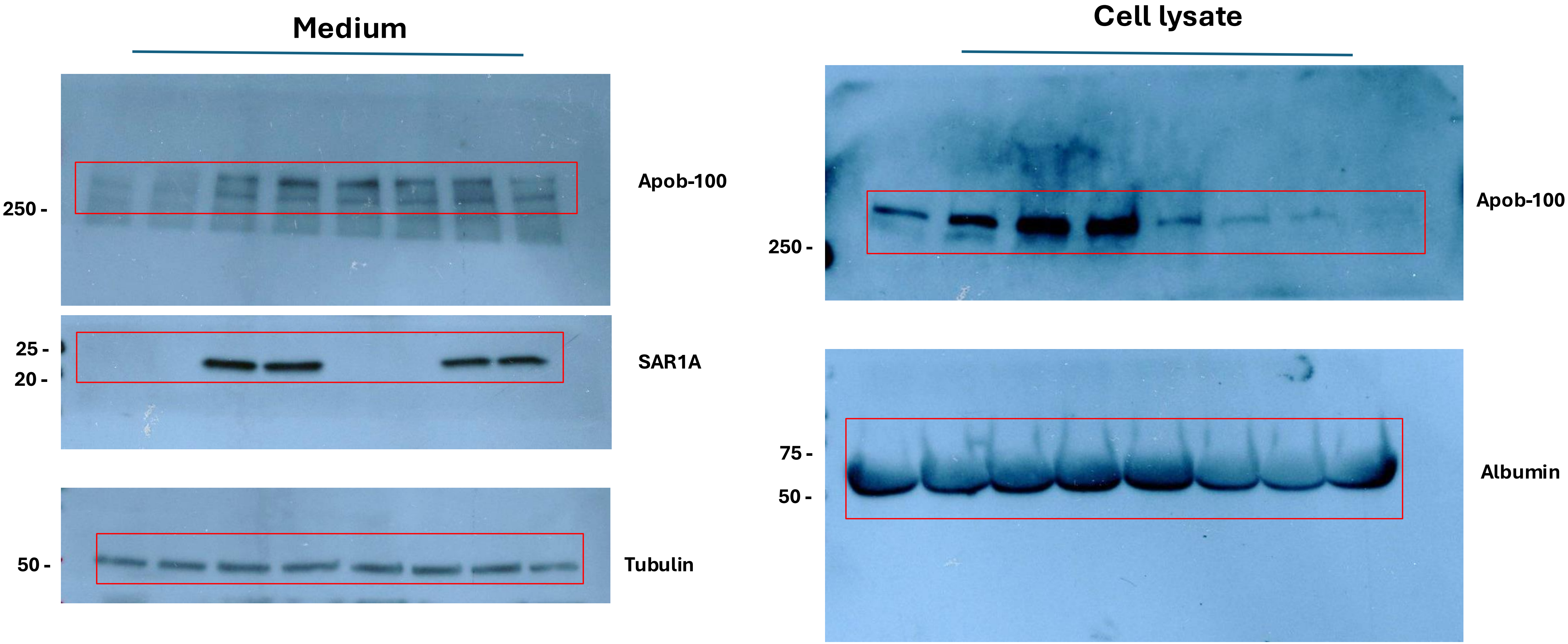
Medium
Cell lysate
Apob-100
250 -
25 -
20 -
SAR1A
50 -
Tubulin
Apob-100
250 -
75 -
50 -
Albumin

Fig. 4K

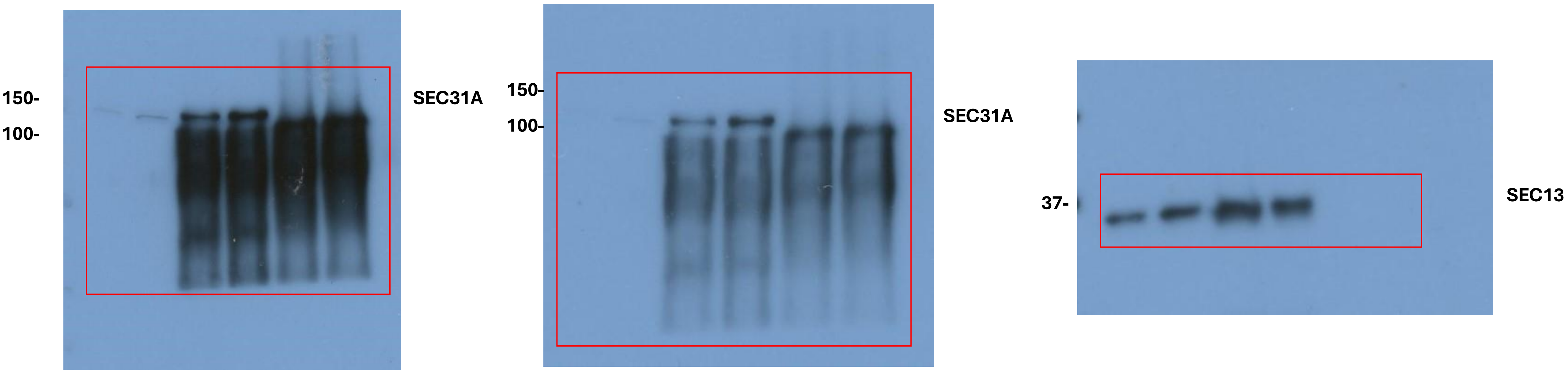

Fig. 4M

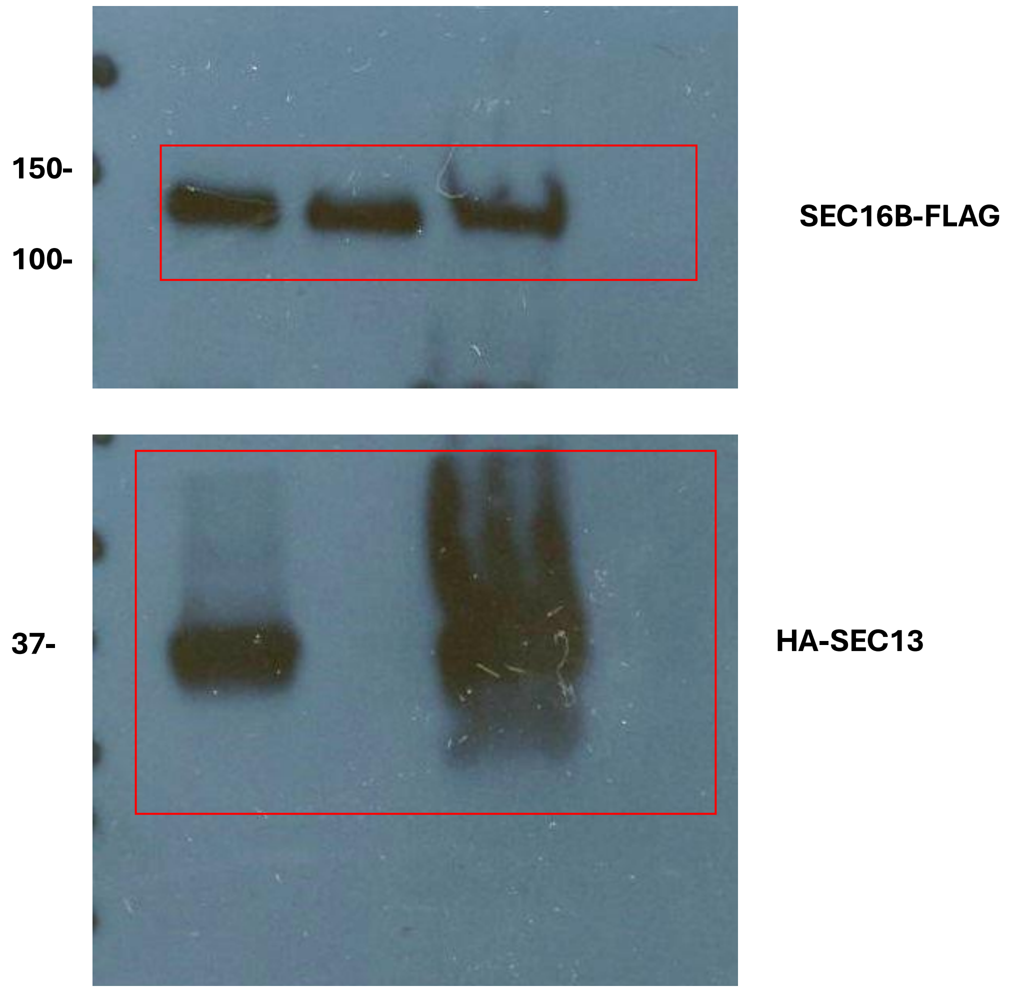

Fig. 6D

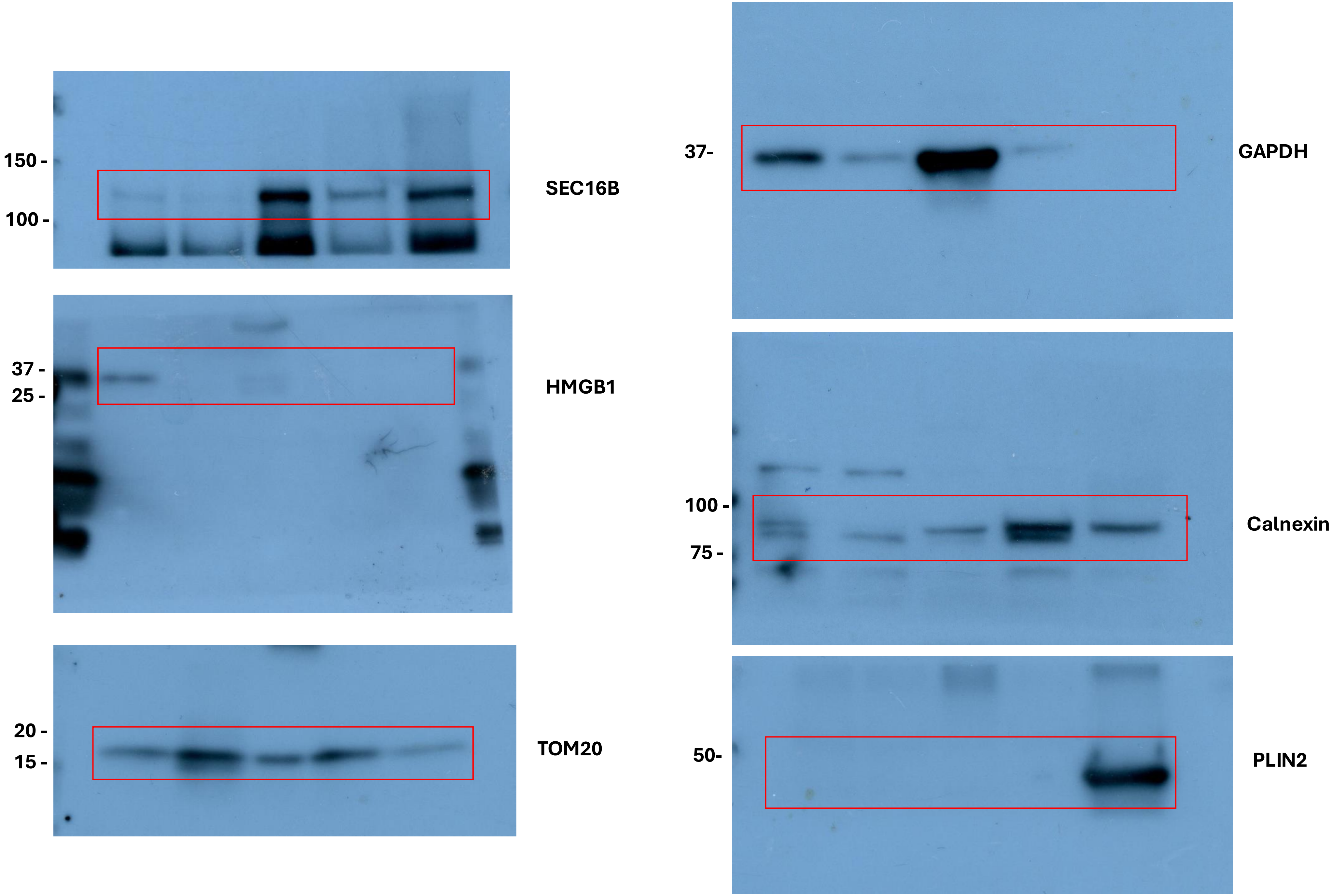
150 -
100 -
SEC16B
37 -
25 -
HMGB1
20 -
15 -
TOM20
37-
GAPDH
100 -
75 -
Calnexin
50-
PLIN2

Supplemental Fig. 4A

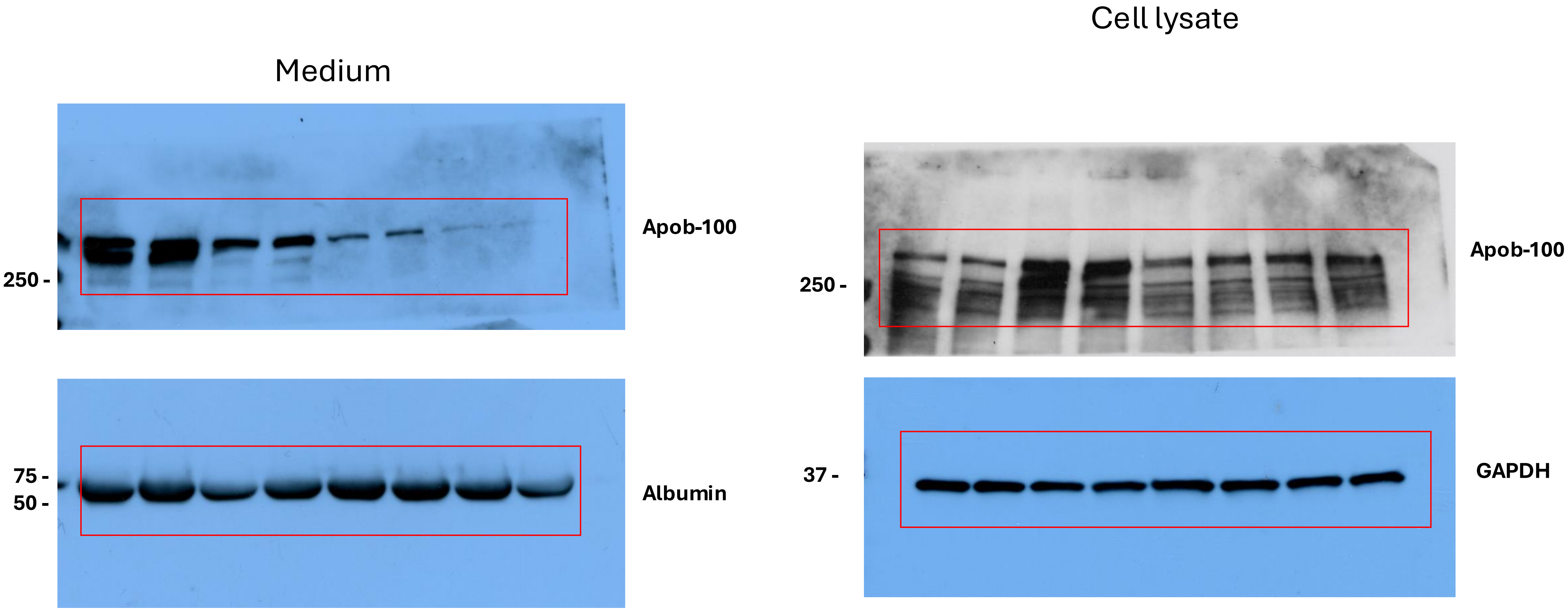

Supplemental Fig. 4G

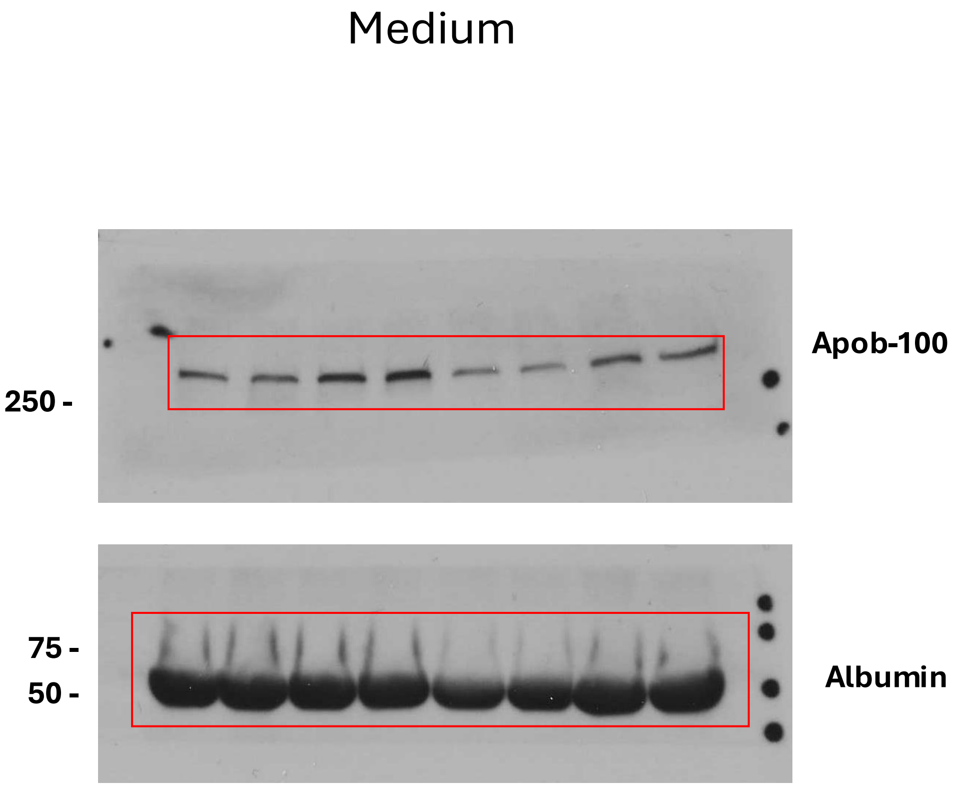

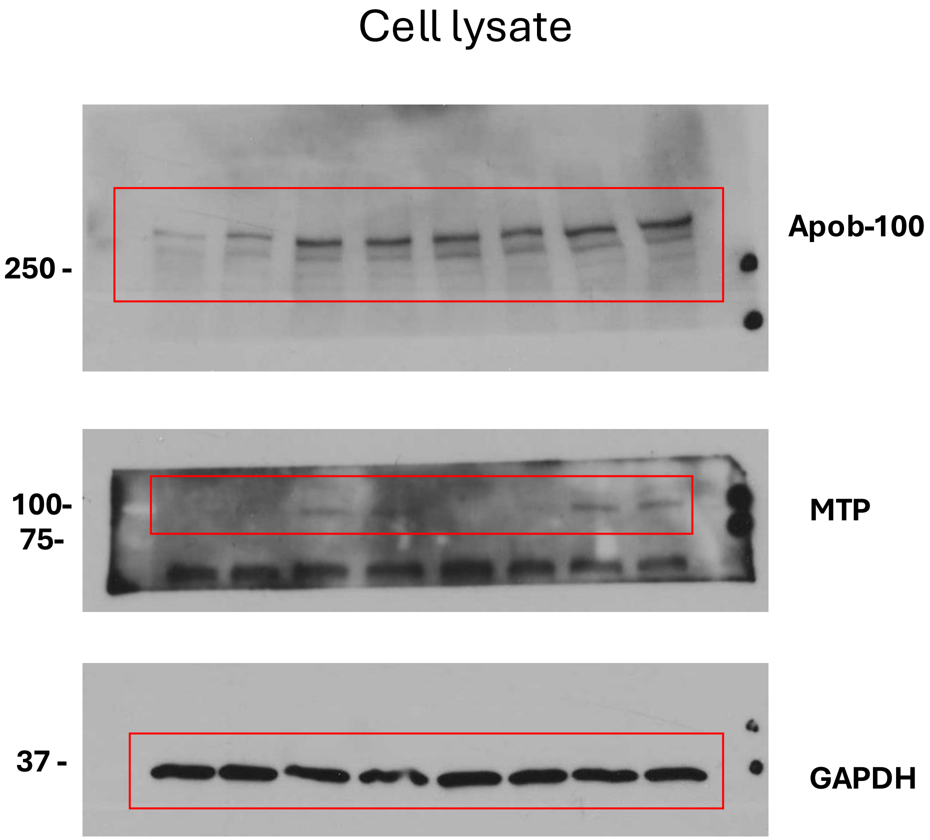

Supplemental Fig. 5A

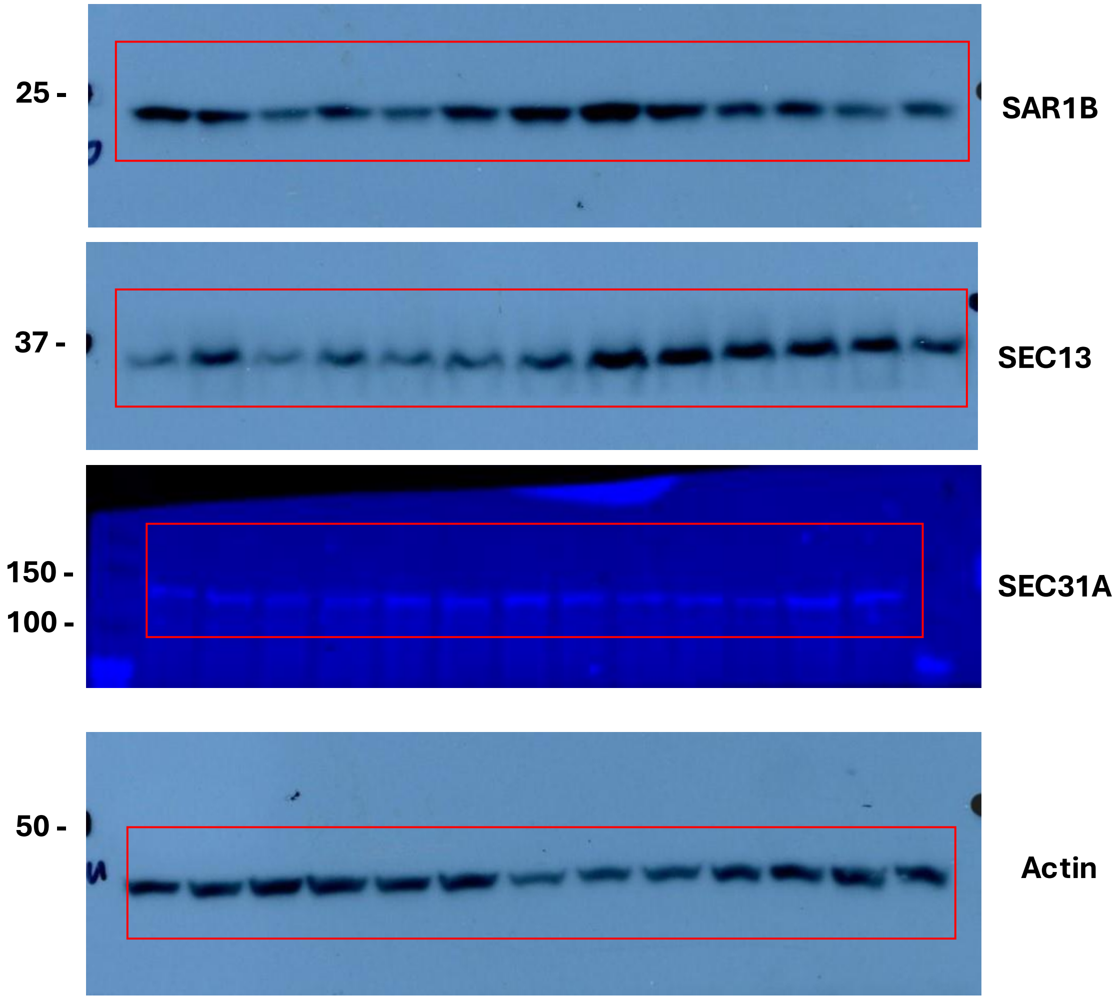

Supplemental Fig. 5B

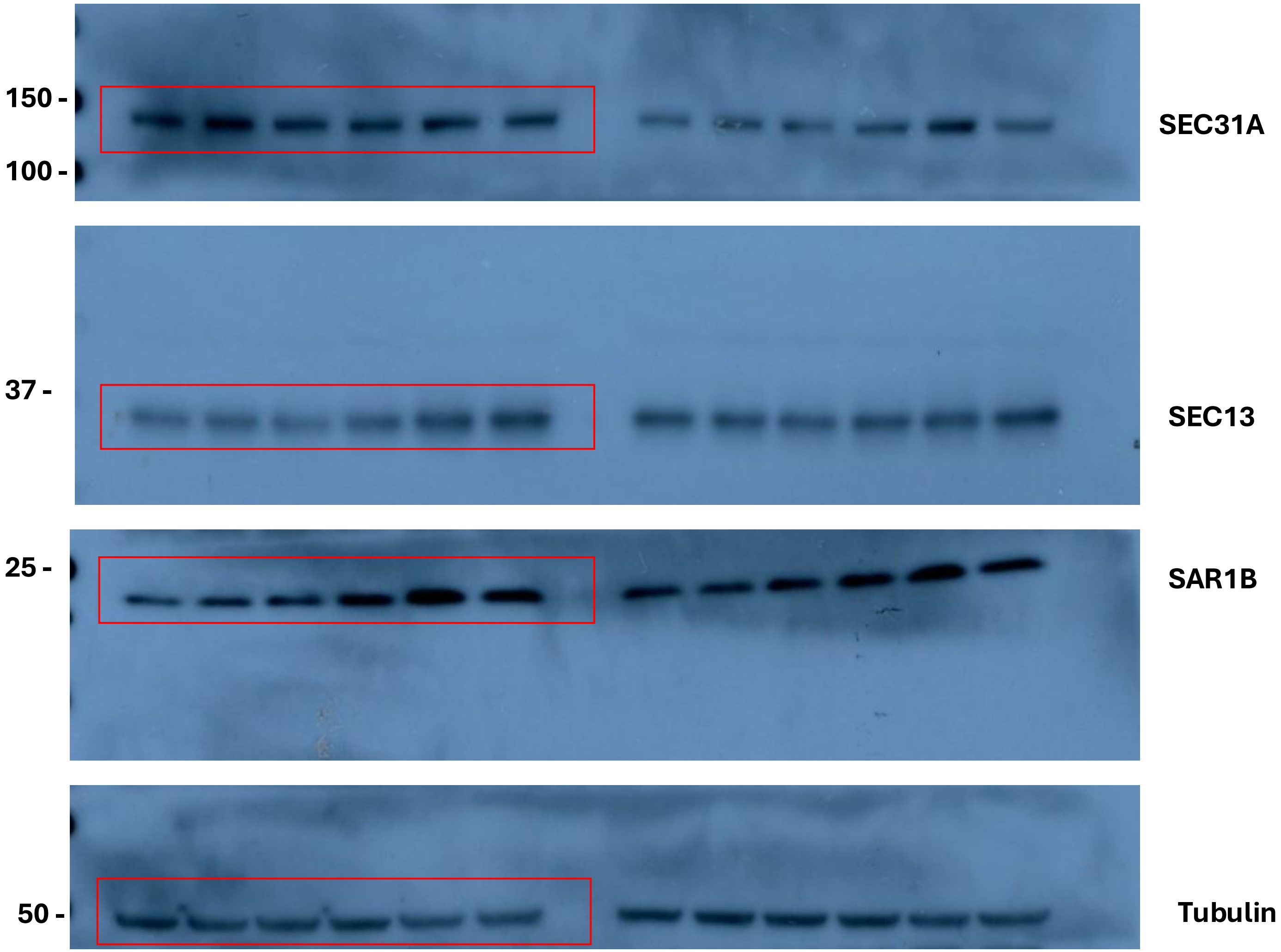

Supplemental Fig. 5C

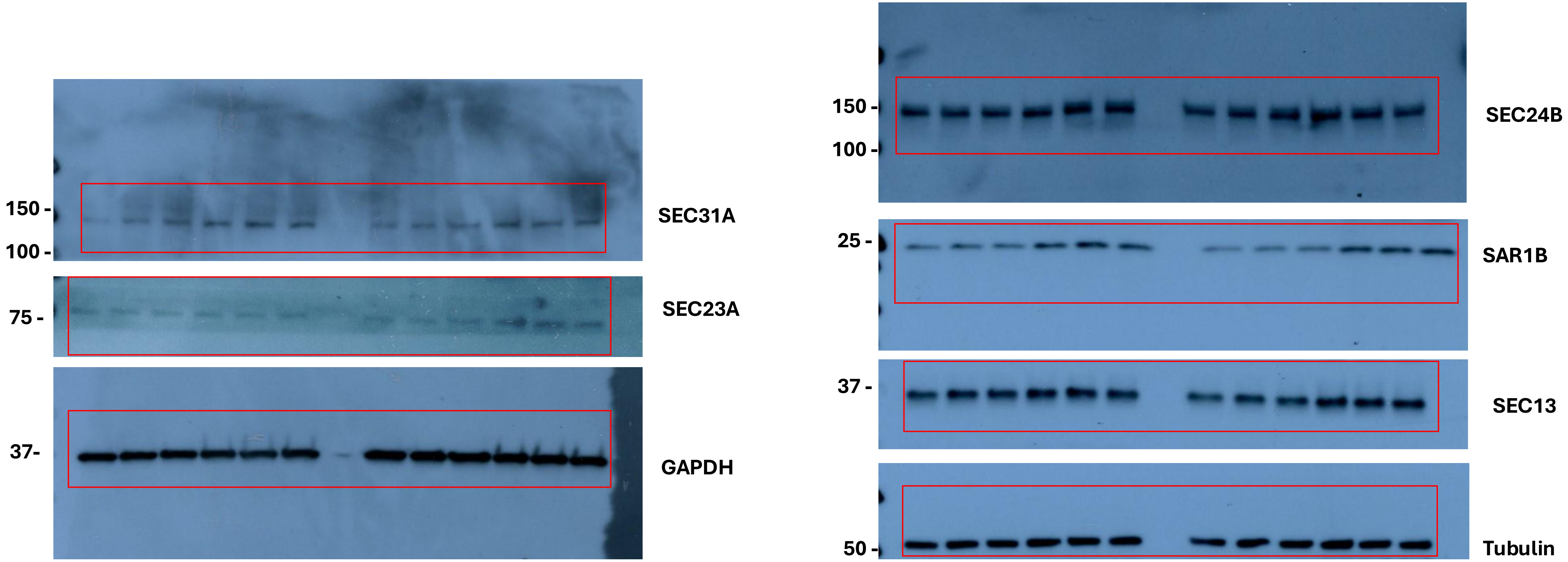

Supplemental Fig. 5G

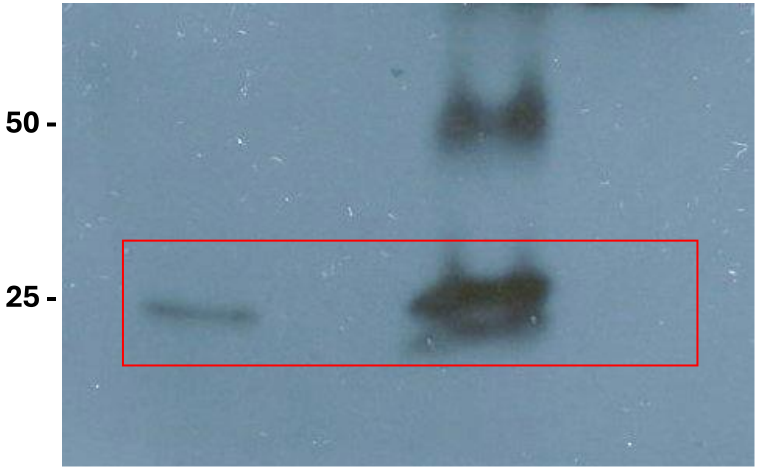

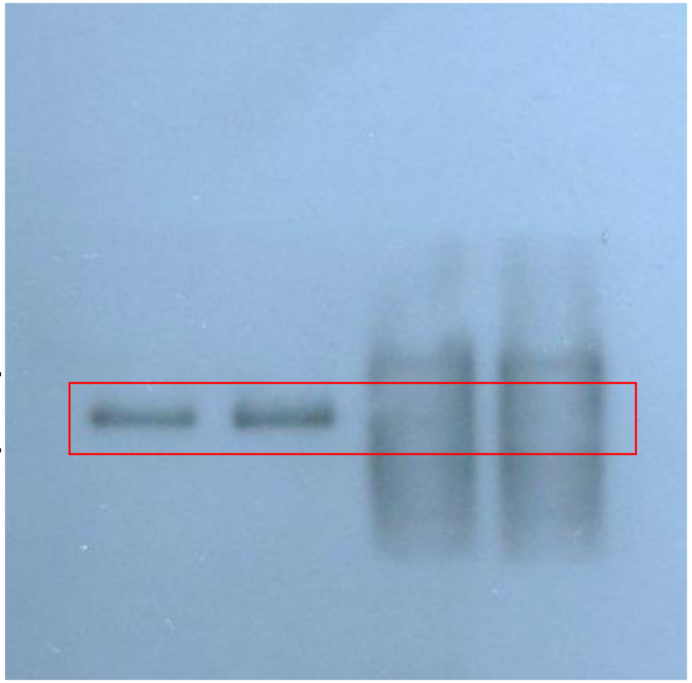

Supplemental Fig. 5H

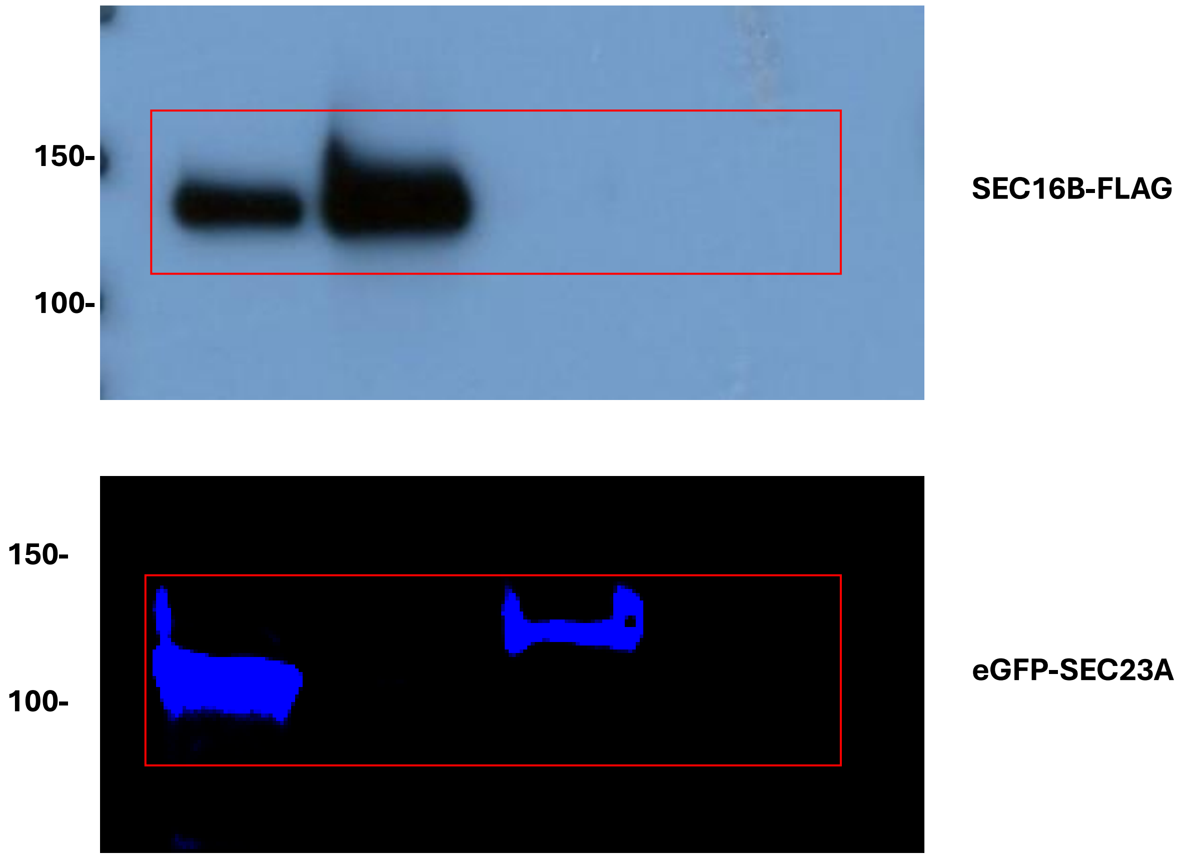

Supplemental Fig. 8D

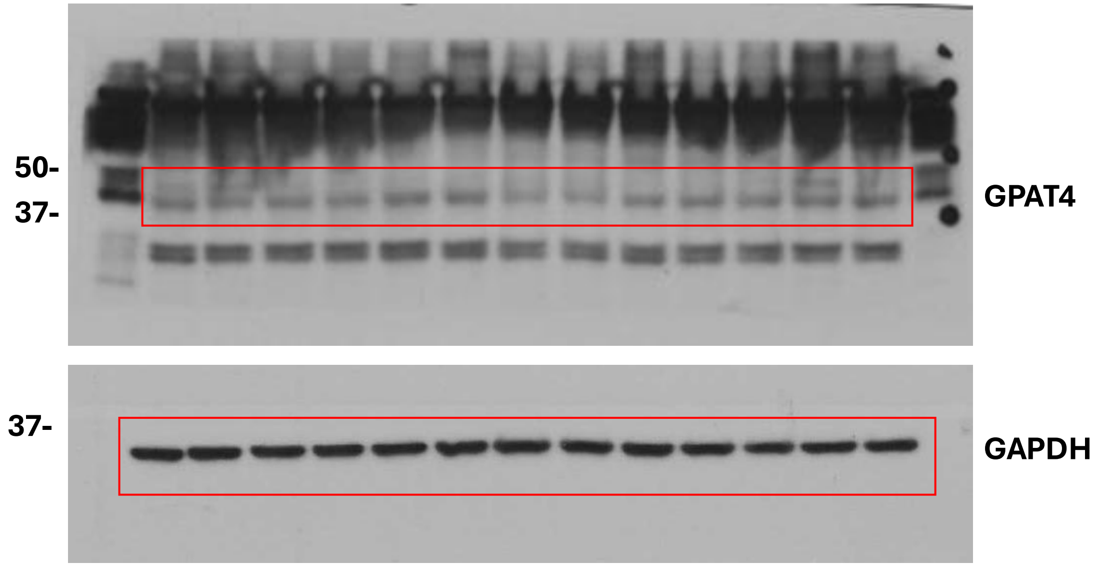

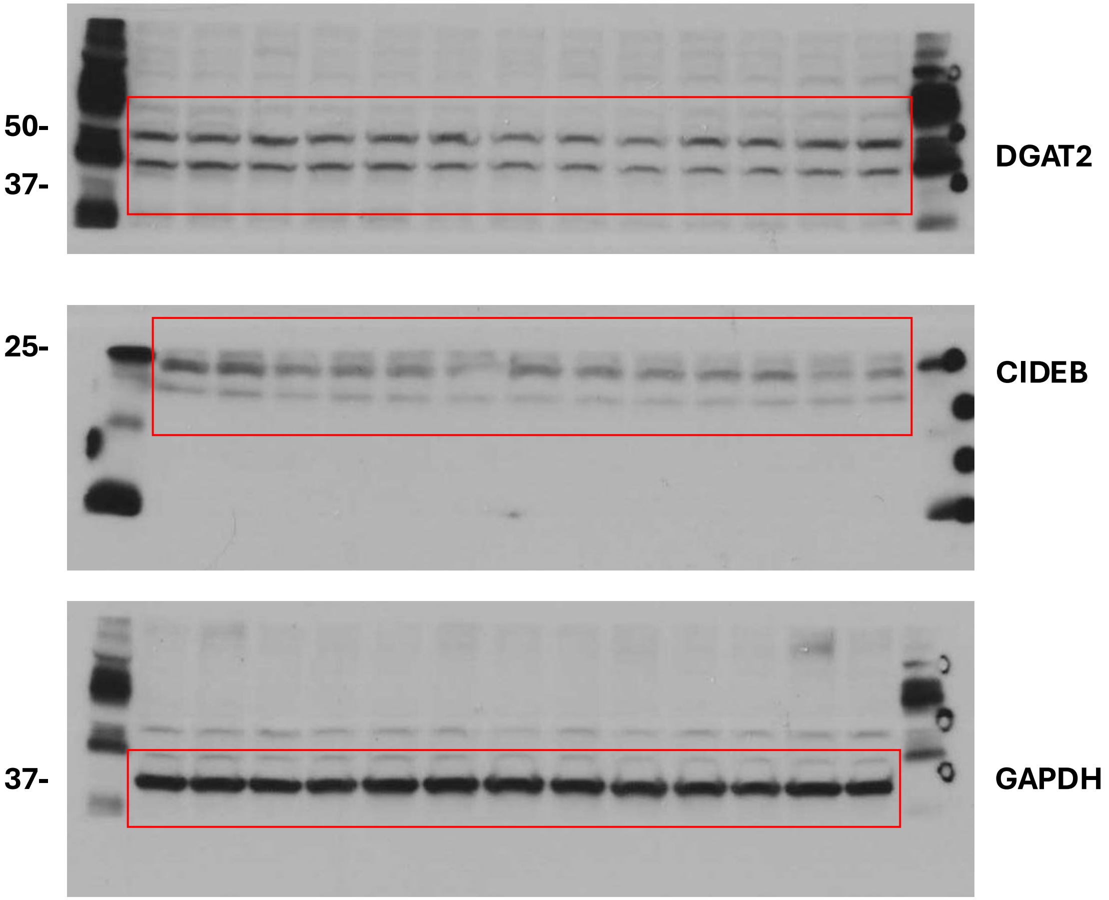

Supplemental Fig. 8F

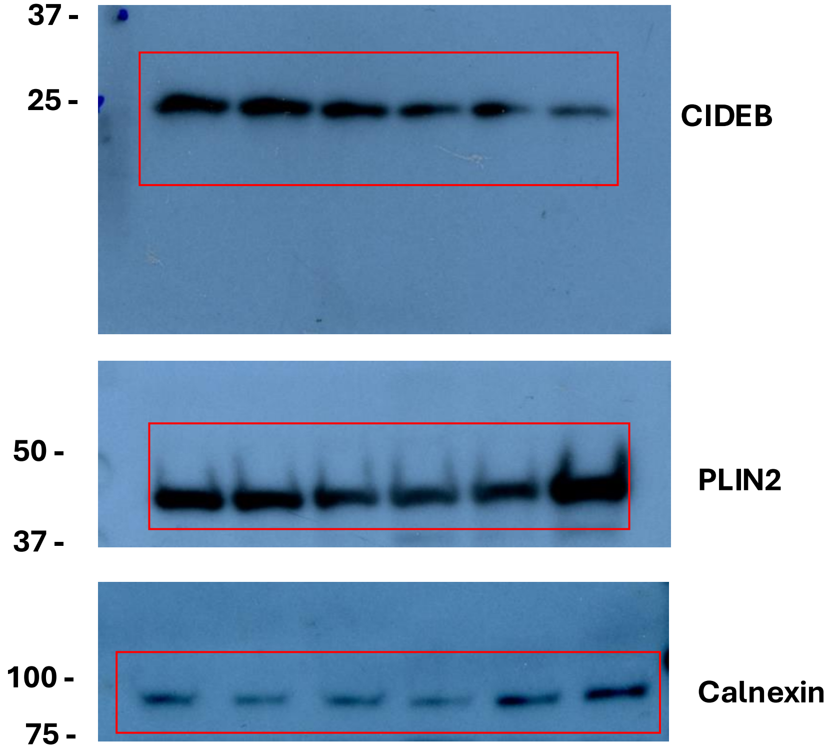

Supplemental Fig. 8G

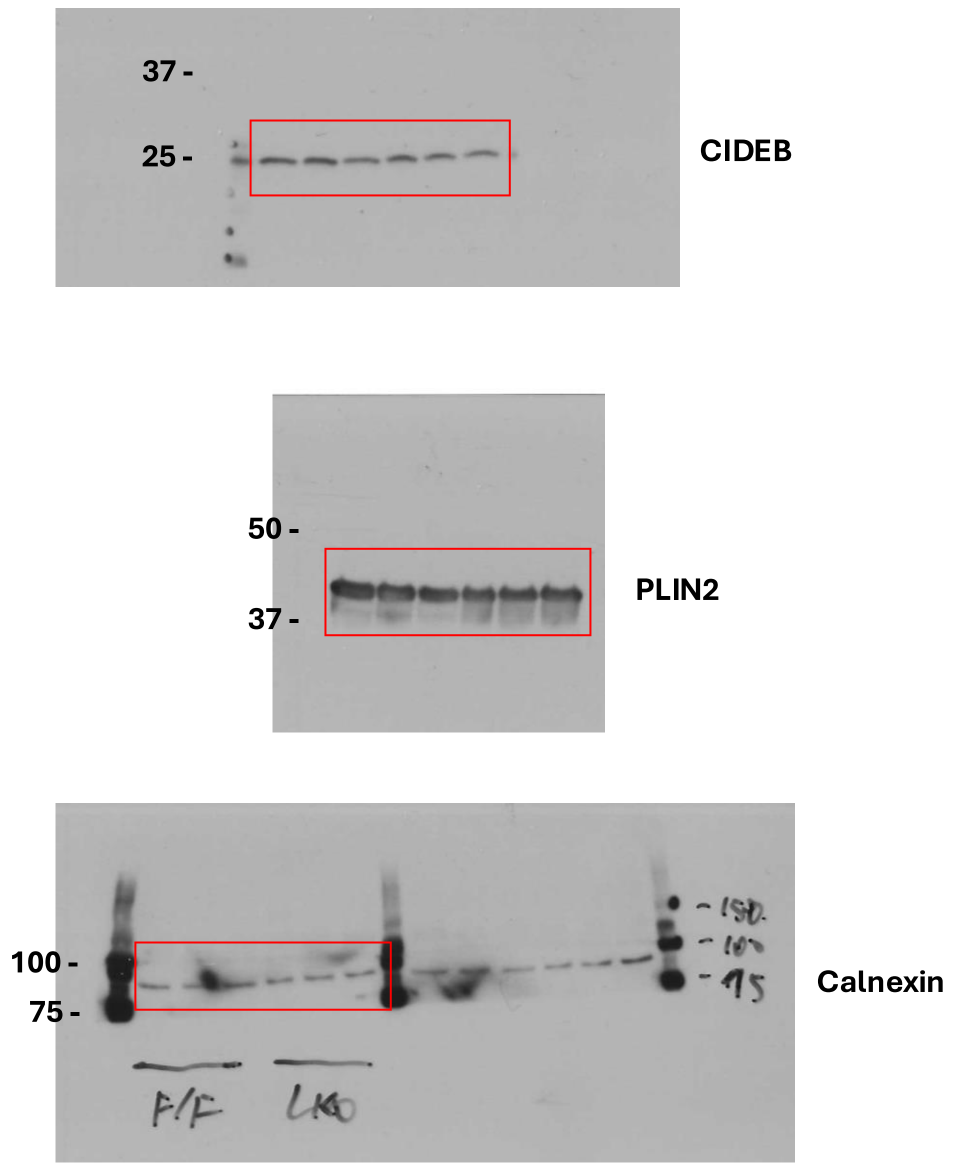

Supplement: Unedited blot and gel images [file jci-136-204602-s022.pdf]
